# Ultraprocessed or minimally processed diets following healthy dietary guidelines on weight and cardiometabolic health: a randomized, crossover trial

**DOI:** 10.1038/s41591-025-03842-0

**Published:** 2025-08-04

**Authors:** Samuel J. Dicken, Friedrich C. Jassil, Adrian Brown, Monika Kalis, Chloe Stanley, Chaniqua Ranson, Tapiwa Ruwona, Sulmaaz Qamar, Caroline Buck, Ritwika Mallik, Nausheen Hamid, Jonathan M. Bird, Alanna Brown, Benjamin Norton, Claudia A. M. Gandini Wheeler-Kingshott, Mark Hamer, Chris van Tulleken, Kevin D. Hall, Abigail Fisher, Janine Makaronidis, Rachel L. Batterham

**Affiliations:** 1 Centre for Obesity Research, Division of Medicine, University College London (UCL), London, UK; 2 Department of Behavioural Science and Health, University College London, London, UK; 3 University College London Hospital (UCLH) Biomedical Research Centre, National Institute for Health Research, London, UK; 4 Bariatric Centre for Weight Management and Metabolic Surgery, University College London Hospital (UCLH), London, UK; 5 Service of Endocrinology, Diabetology, Nutrition and Therapeutic Education, Department of Medicine, Geneva University Hospitals, Geneva, Switzerland; 6 Pain Management Centre, The National Hospital for Neurology and Neurosurgery, London, UK; 7 Institute of Sport Exercise and Health, Division of Surgery and Interventional Science UCL, London, UK; 8 Digestive Disease and Surgery Institute, Cleveland Clinic London, London, UK; 9 NMR Research Unit, Queen Square MS Centre, Department of Neuroinflammation, UCL Institute of Neurology, Faculty of Brain Sciences, University College London, London, UK; 10 Department of Brain and Behavioural Sciences, University of Pavia, Pavia, Italy; 11 Digital Neuroscience Center, IRCCS Mondino Foundation, Pavia, Italy; 12 Division of Infection, University College London, London, UK; 13 Independent Researcher, Kensington, MD USA; 14 Department of Diabetes and Metabolism, Royal London Hospital, Barts Health NHS Trust, London, UK

**Keywords:** Obesity, Obesity, Randomized controlled trials, Lifestyle modification, Obesity

## Abstract

Ultraprocessed food (UPF) consumption is associated with noncommunicable disease risk, yet no trial has assessed its health impact within the context of national dietary guidelines. In a 2 × 2 crossover randomized controlled feeding trial, 55 adults in England (body mass index ≥25 to <40 kg m^−2^, habitual UPF intake ≥50% kcal day^−1^) were provided with two 8-week ad libitum diets following the UK Eatwell Guide: (1) minimally processed food (MPF) and (2) UPF, in a random order. Twenty-eight people were randomized to MPF then UPF, and 27 to UPF then MPF; 50 participants comprised the intention-to-treat sample. The primary outcome was the within-participant difference in percent weight change (%WC) between diets, from baseline to week 8. Participants were blinded to the primary outcome. MPF (%WC, −2.06 (95% confidence interval (CI), −2.99, −1.13) and UPF (%WC, −1.05 (95% CI, −1.98, −0.13)) resulted in weight loss, with significantly greater %WC on MPF (Δ%WC, −1.01 (95% CI, −1.87, −0.14), *P* = 0.024; Cohen’s *d*, −0.48 (95% CI, −0.91, −0.06)). Mild gastrointestinal adverse events were common on both diets. Findings indicate greater weight loss on MPF than UPF diets and needing dietary guidance on food processing in addition to existing recommendations. Clinicaltrials.gov registration: NCT05627570.

## Abstract (web summary)

A free-living trial in people with overweight or obesity found that minimally processed diets led to greater weight loss and cardiometabolic improvements than ultraprocessed diets following UK healthy eating guidelines at 8 weeks.

## Main

Three billion people worldwide live with overweight or obesity^1^, driving increased risks of noncommunicable disease and early death^2^. A proposed cause has been from recent major changes in the food environment^3^. In particular, the increased accessibility and consumption of ultraprocessed food (UPF)^3,4^. Most commonly defined using the Nova classification^5^, UPF are industrial formulations combining extracts of original foods with additives and industrial ingredients^5^. Examples include breakfast cereals, sweets, and mass-produced bread^5^. Over 50% of UK energy intake is reported to come from UPF^6^, with similarly high intakes in the USA and Europe^7^. Higher UPF intakes are associated with increased risks of obesity^8^, cardiometabolic disease, and all-cause mortality^9,10^. As a result, countries including Brazil^11^ and organizations including the World Health Organization^12^ recommend reducing UPF intake in their dietary guidance. In the UK, where nearly two-thirds of adults live with overweight or obesity^13^, calls have been made for policy action on reducing UPF, yet this is still debated^14^.

The Eatwell Guide (EWG) provides the UK public with guidance on a healthy diet^15,16^, following recommendations by the Scientific Advisory Committee on Nutrition (SACN). The EWG focuses on macronutrients (for example, fat, protein, carbohydrate) and food groups (for example, fruits and vegetables, dairy, starchy food), but not UPF^16^. Currently fewer than 0.1% of UK adults follow EWG recommendations, and nearly 70% follow less than half of the recommendations^17^. SACN recently reported that there was insufficient evidence to include UPF within dietary guidelines^18^, with the 2025 US Dietary Guidelines for Americans Committee (DGAC) reaching similar conclusions^19^.

Both SACN and the US DGAC^19^ recommended that randomized controlled trials (RCTs) are needed due to the lack of high-quality interventional evidence^18^. SACN particularly recommended trials comparing UPF with minimally processed food (MPF) in the context of existing UK dietary recommendations^18^. Current evidence suggests that the associations between UPF and adverse health outcomes are not explained by macronutrient or food group guidance within dietary recommendations^10^. To date, two RCTs demonstrate unfavorable weight changes on UPF compared with MPF diets matched for presented energy and nutrients^20,21^, but no RCTs have assessed the health impact of food processing in the context of dietary guidance.

**Fig. 1:**
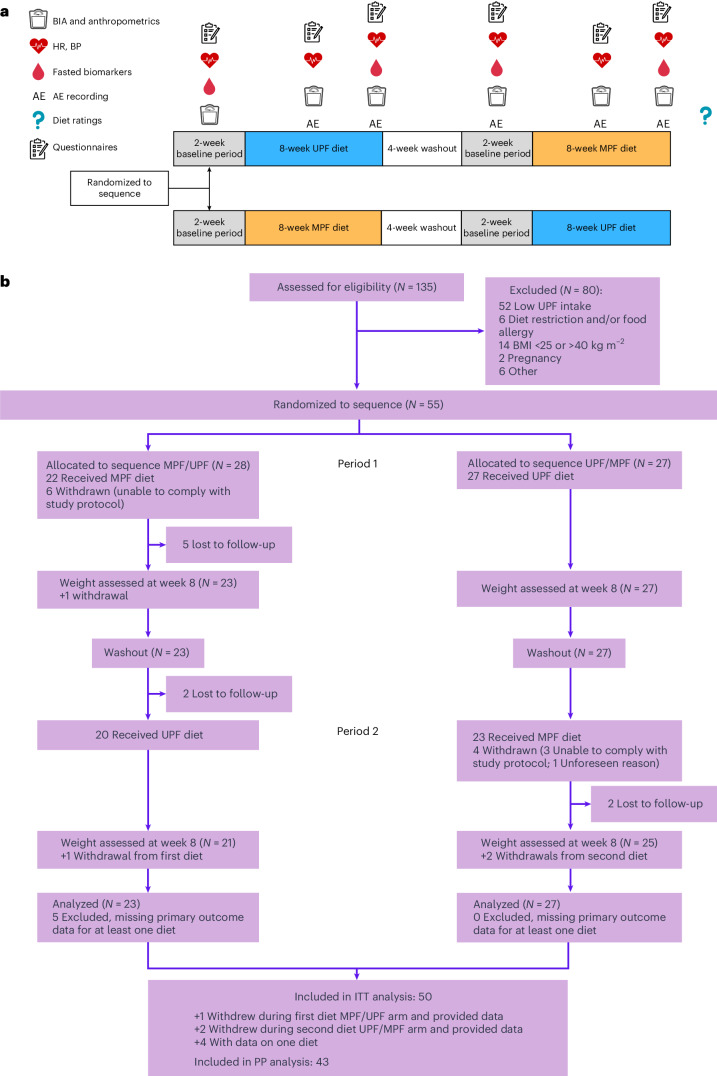
UPDATE study design, measurement timepoints and CONSORT diagram. **a**, UPDATE study design and measurement timepoints. **b**, Consolidated Standards of Reporting Trials (CONSORT) diagram.

Therefore, ‘Ultra processed versus minimally processed diets following UK dietary guidance on health outcomes’ (UPDATE)—a single-center, community-based, 2 × 2 crossover RCT—aimed to compare the health effects between 8-week MPF and UPF diets following EWG recommendations (Fig. 1a)^22^. The primary objective was to compare percentage weight change (%WC) between diets. Secondary objectives were to compare changes in anthropometrics, body composition, cardiometabolic and appetite-related outcomes between diets.

## Results

### Participant disposition

Between April 2023 and May 2024, 135 adults underwent screening, of whom 55 (40.1%) were eligible and allocated randomly to either MPF then UPF (*n* = 28), or UPF then MPF (*n* = 27) diets. The first and last participants were enrolled on 3 April 2023 and 7 May 2024, respectively. Figure 1b presents participant flow. Six participants withdrew during the first-period MPF diet (sequence MPF/UPF), two during washout (sequence MPF/UPF) and four during the second-period MPF diet (sequence UPF/MPF). In total, 50 participants provided primary outcome data for at least one diet (intention-to-treat (ITT)) and 43 provided primary outcome data for both diets without withdrawal (per protocol (PP)). Baseline characteristics are given in Table 1. Mean age was 43.2 years (s.e., 1.5), 36 (65.5%) were of white ethnicity, 50 (90.9%) were female and nine (16.4%) were nightshift workers. Mean weight was 89.4 kg (s.e., 1.7), and body mass index (BMI) 32.7 kg m^−2^ (s.e., 0.5). Mean habitual UPF intake was 67.4% kcal day^−1^ (s.e., 1.1), with mean macronutrient and food group intakes not adherent to EWG recommendations, except red meat intake. Baseline characteristics for ITT and PP samples are in Supplementary Tables 1–3.

**Table 1:** Baseline demographic and clinical characteristics overall and by randomization arm

*N* = 55	Overall	By randomization arm	
		MPF/UPF (28)	UPF/MPF (27)
Age in years at screening	43.2 (1.54)	43.5 (2.42)	42.9 (1.9)
Female sex	50 (90.9%)	25 (89.3%)	25 (92.6%)
White ethnicity	36 (65.5%)	21 (75.0%)	15 (55.6%)
Occupation			
Doctor	1 (1.8%)	1 (3.6%)	0 (0.0%)
Nurse	24 (43.6%)	9 (32.1%)	15 (55.6%)
AHP	9 (16.4%)	5 (17.9%)	4 (14.8%)
Management	5 (9.1%)	3 (10.7%)	2 (7.4%)
Administrative	11 (20.0%)	6 (21.4%)	5 (18.5%)
Other	5 (9.1%)	4 (14.3%)	1 (3.7%)
Nightshift worker	9 (16.4%)	4 (14.3%)	5 (18.5%)
Education level			
None	0 (0.0%)	0 (0.0%)	0 (0.0%)
GCSE/O-level equivalent	4 (7.3%)	3 (10.7%)	1 (3.7%)
A level or equivalent	4 (7.3%)	1 (3.6%)	3 (11.1%)
Degree	26 (47.3%)	14 (50.0%)	12 (44.4%)
Postgraduate	21 (38.2%)	10 (35.7%)	11 (40.7%)
Other	0 (0.0%)	0 (0.0%)	0 (0.0%)
Marital status			
Single	21 (38.2%)	12 (42.9%)	9 (33.3%)
Married	17 (30.9%)	9 (32.1%)	8 (29.6%)
Living together	6 (10.9%)	2 (7.1%)	4 (14.8%)
Separated	3 (5.5%)	3 (10.7%)	0 (0.0%)
Divorced	4 (7.3%)	1 (3.6%)	3 (11.1%)
Widowed	4 (7.3%)	1 (3.6%)	3 (11.1%)
Civil partnership	0 (0.0%)	0 (0.0%)	0 (0.0%)
Family history of obesity	30 (54.5%)	15 (53.6%)	15 (55.6%)
Family history of diabetes	17 (30.9%)	7 (25.0%)	10 (37.0%)
Family history of cardiovascular disease	21 (38.2%)	10 (35.7%)	11 (40.7%)
Smoking			
Yes, current	5 (9.1%)	2 (7.1%)	3 (11.1%)
Yes past	17 (30.9%)	6 (21.4%)	11 (40.7%)
No, never	33 (60.0%)	20 (71.4%)	13 (48.2%)
AUDIT-C	2.55 (0.20)	2.64 (0.32)	2.44 (0.24)
Weekly units of alcohol	3.30 (0.63)	3.34 (1.08)	3.26 (0.66)
Weight (kg)	89.41 (1.74)	91.55 (2.65)	87.20 (2.22)
Height (m)	1.65 (0.01)	1.66 (0.01)	1.64 (0.01)
Estimated basal metabolic rate (kcal)	1,673.64 (28.43)	1,703.61 (44.66)	1,642.56 (34.63)
BMI (kg m^−2^)	32.72 (0.53)	33.16 (0.73)	32.26 (0.77)
BMI			
25–29.9 kg m^−2^	18 (33%)	7 (25.0%)	11 (40.7%)
30–34.9 kg m^−2^	21 (38%)	12 (42.9%)	9 (33.3%)
35–39.9 kg m^−2^	16 (29%)	9 (32.14%)	7 (25.93%)
Waist circumference (cm)	97.25 (1.45)	99.68 (2.11)	94.72 (1.89)
Waist-to-height ratio	0.59 (0.01)	0.60 (0.01)	0.58 (0.01)
SBP (mm Hg)	130.24 (1.84)	133.43 (2.68)	126.93 (2.39)
DBP (mm Hg)	74.85 (1.26)	76.82 (1.76)	72.81 (1.74)
SBP > 140 mm Hg or DBP > 90 mm Hg	15 (27.3%)	8 (28.6%)	7 (25.9%)
HbA1c (%)	5.51 (0.06)	5.42 (0.10)	5.61 (0.07)
Fasting glucose (mmol l^−1^)	4.80 (4.60–5.10)	4.85 (4.60–5.12)	4.80 (4.60–5.10)
Total cholesterol (mmol ^l^^−1^) (*N* = 53)	5.21 (0.12)	5.18 (0.17) (*N* = 27)	5.24 (0.16) (*N* = 26)
Total cholesterol >5 mmol l^−1^ (*N* = 53)	33 (62%)	17 (63.0%)	16 (61.5%)
Participants self-reporting a condition	36 (65.5%)	21 (75.0%)	15 (55.6%)
Participants self-reporting medication use	36 (65.5%)	22 (78.6%)	14 (51.9%)
Self-reported antihypertensive use	8 (14.5%)	6 (21.4%)	2 (7.4%)
Self-reported hypercholesterolemia medication use	3 (5.5%)	2 (7.1%)	1 (3.7%)
Total energy (kcal day^−1^)	2,122.5 (88.6)	2,010.4 (103.7)	2,238.8 (143.6)
MPF (percentage of kcal day^−1^)	22.4 (0.9)	21.0 (1.4)	23.8 (1.1)
PCI (percentage of kcal day^−1^)	2.4 (0.4)	1.9 (0.5)	3.0 (0.5)
PF (percentage of kcal day^−1^)	7.6 (0.7)	7.9 (1.1)	7.1 (0.8)
UPF (percentage of kcal day^−1^)	67.4 (1.1)	69.2 (1.5)	65.6 (1.5)
Fat (percentage of kcal day^−1^)	37.0 (0.5)	36.0 (0.7)	38.1 (0.8)
Saturated fat (percentage of kcal day^−1^)	13.4 (0.4)	12.8 (0.4)	14.0 (0.6)
Carbohydrate (percentage of kcal day^−1^)	44.5 (0.7)	45.3 (1.0)	43.8 (0.8)
Total sugar (percentage of kcal day^−1^)	17.0 (0.7)	16.7 (1.1)	17.2 (0.8)
Total free sugar (percentage of kcal day^−1^)	10.1 (0.6)	10.1 (0.9)	10.1 (0.7)
Salt (g day^−1^)	6.1 (0.3)	5.8 (0.3)	6.3 (0.4)
Protein (percentage of kcal day^−1^)	16.2 (0.4)	16.3 (0.5)	16.0 (0.5)
Fiber (g day^−1^)	19.6 (1.0)	18.4 (1.1)	20.9 (1.7)
Alcohol (percentage of kcal day^−1^)	2.2 (0.5)	2.4 (0.7)	2.1 (0.7)
Red meat (g day^−1^)	54.5 (5.2)	53.2 (6.2)	55.8 (8.4)
Oily fish (g day^−1^)	8.0 (1.9)	6.4 (2.8)	9.7 (2.7)
Fruit and vegetables (portions day^−1^)	3.3 (0.2)	3.2 (0.3)	3.3 (0.3)
Average daily MVPA (min) (*N* = 48)	47.2 (3.7)	40.3 (5.0) (*N* = 24)	54.2 (5.3) (*N* = 24)

Data is presented as mean, count or median where appropriate with corresponding percentage, s.e. or interquartile range, respectively, in brackets. AHP, allied health professional; AUDIT-C, alcohol use disorders identification test–consumption; PCI, processed culinary ingredient; PF, processed food.

Sociodemographic, clinical and dietary and baseline characteristics were similar between dropouts (*N* = 12) and nondropouts (*N* = 43) (Supplementary Table 4).

### Primary outcome

**Fig. 2:**
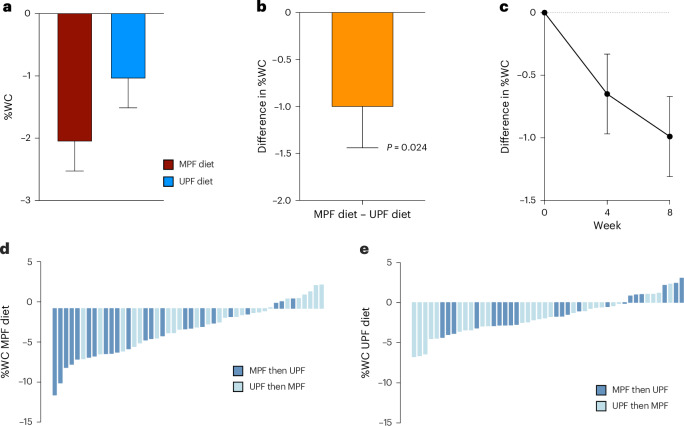
Percent weight change on MPF and UPF diets. Estimated marginal means and s.e. values computed from mixed-effects models adjusted for randomization arm and nightshift status, with an interaction term for diet and randomization arm and a random effect for participant; ITT *N* = 50. **a**, The %WC on MPF and UPF diets from estimated marginal means. **b**, Difference in %WC between MPF and UPF diets from estimated marginal means; two-sided *t* statistic (degrees of freedom, 46.1), Cohen’s *d*, −0.48 (95% CI, −0.91, −0.06); *P* = 0.024, not adjusted for multiple comparisons. **c**, Difference in %WC between MPF and UPF diets from repeated-measures mixed-effects model estimated marginal means. **d**, Unadjusted %WC on the minimally processed diet for each participant. **e**, Unadjusted %WC on the UPF diet for each participant.

In the ITT analysis, %WC at 8 weeks was significantly lower on both diets (MPF, −2.06% ((95% confidence interval (CI), −2.99, −1.13); UPF, −1.05% (95% CI, −1.98, −0.13)) (Fig. 2). Within-participant differences in %WC were significantly greater on the MPF versus UPF diet (Δ%WC, −1.01% (s.e., 0.43; 95% CI, −1.87, −0.14; *P* = 0.024). Unadjusted changes are in Supplementary Table 5.

**Extended Data Fig. 1:**
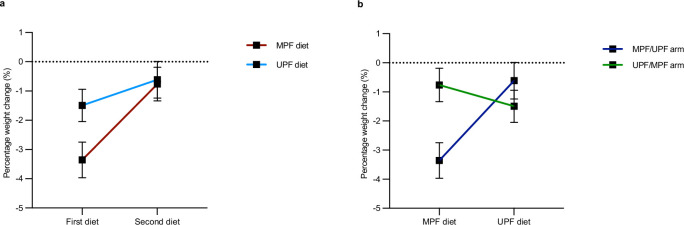
Primary outcome order effect, percentage weight change presented by (a) diet order and (b) randomisation arm. Estimated marginal means and standard errors computed from mixed-effects models adjusted for randomisation arm and night-shift status, with an interaction effect for diet and randomisation arm, and a random effect for participant. Abbreviations: MPF: minimally processed food; UPF: ultra-processed food. Intention-to-treat N = 50.

Results by randomization arm are in Supplementary Table 6. A significant diet order effect was detected (*P*_interaction_ < 0.05; Extended Data Fig. 1). A larger Δ%WC was observed when analyzing the first-period diet of each arm only (Δ%WC, −1.86% (s.e., 0.72); *P* = 0.012).

### Secondary outcomes

Secondary ITT outcomes are in Table 2, with unadjusted changes and changes by randomization arm in Supplementary Tables 5 and 6, respectively.

**Table 2:** Changes in secondary outcomes from baseline to week 8 on each diet, and differences in changes in outcomes from baseline to week 8 between diets

ITT *N* = 50	MPF diet			UPF diet			MPF diet − UPF diet				
	Mean	Lower 95% CI	Upper 95% CI	Mean	Lower 95% CI	Upper 95% CI	Mean	s.e.	Lower 95% CI	Upper 95% CI	*P* value
Weight (kg)	−1.84	−2.68	−1.00	−0.88	−1.72	−0.05	−0.96	0.40	−1.76	−0.17	0.019
BMI (kg m^−2^)	−0.67	−0.98	−0.37	−0.33	−0.63	−0.03	−0.34	0.14	−0.63	−0.05	0.021
Waist circumference (cm)	−1.70	−3.52	0.12	−0.18	−1.94	1.57	−1.51	1.03	−3.59	0.56	0.148
Fat mass (kg)	−1.59	−2.32	−0.85	−0.61	−1.34	0.12	−0.98	0.32	−1.62	−0.33	0.004
Body fat percentage (%)	−1.08	−1.66	−0.51	−0.32	−0.89	0.24	−0.76	0.28	−1.33	−0.19	0.010
Visceral fat rating	−0.57	−0.81	−0.32	−0.16	−0.40	0.08	−0.41	0.15	−0.70	−0.11	0.008
Fat-free mass (kg)	−0.30	−0.78	0.19	−0.30	−0.77	0.18	0.00	0.28	−0.57	0.57	0.993
Muscle mass (kg)	−0.29	−0.75	0.17	−0.28	−0.73	0.17	−0.01	0.27	−0.55	0.54	0.980
Bone mass (kg)	−0.01	−0.03	0.02	−0.02	−0.04	0.01	0.01	0.02	−0.02	0.04	0.569
Total body water mass (kg)	−0.65	−0.96	−0.33	−0.14	−0.45	0.17	−0.51	0.15	−0.81	−0.20	0.002
Total body water percentage (%)	0.21	−0.15	0.56	0.30	−0.05	0.65	−0.10	0.16	−0.42	0.23	0.563
SBP (mm Hg)	−5.75	−10.13	−1.36	−2.67	−7.01	1.67	−3.08	2.44	−7.99	1.84	0.214
DBP (mm Hg)	−3.52	−6.02	−1.02	−1.88	−4.35	0.60	−1.64	1.46	−4.59	1.31	0.268
HR (beats per minute)	−3.57	−7.56	0.42	−4.35	−8.28	−0.42	0.78	2.36	−3.97	5.53	0.743
Bilirubin (μmol l^−1^)	0.15	−1.05	1.36	0.70	−0.47	1.87	−0.54	0.49	−1.53	0.45	0.274
Alkaline phosphatase (IU l^−1^)	0.33	−3.17	3.83	−0.51	−3.78	2.77	0.83	2.08	−3.37	5.04	0.690
Alanine transaminase (IU l^−1^)	1.99	−3.34	7.32	−0.52	−5.53	4.50	2.51	2.63	−2.83	7.84	0.347
Albumin (g l^−1^)	−0.18	−1.01	0.64	−0.09	−0.89	0.70	−0.09	0.48	−1.06	0.88	0.852
HbA1c (%)	−0.08	−0.15	−0.01	−0.03	−0.09	0.04	−0.06	0.04	−0.14	0.02	0.165
Fasting glucose (mmol l^−1^)	−0.13	−0.27	0.00	−0.19	−0.32	−0.06	0.05	0.08	−0.10	0.21	0.488
Total cholesterol (mmol l^−1^)	−0.31	−0.51	−0.10	−0.44	−0.63	−0.24	0.13	0.12	−0.11	0.37	0.283
Total-cholesterol-to-HDL ratio	−0.04	−0.21	0.13	−0.13	−0.30	0.03	0.09	0.10	−0.11	0.29	0.368
HDL-C (mmol l^−1^)	−0.11	−0.19	−0.03	−0.08	−0.16	−0.01	−0.03	0.05	−0.12	0.07	0.575
LDL-C (mmol l^−1^)	−0.13	−0.30	0.03	−0.38	−0.54	−0.22	0.25	0.10	0.05	0.45	0.016
Non-HDL-C (mmol l^−1^)	−0.22	−0.41	−0.03	−0.36	−0.54	−0.17	0.14	0.11	−0.09	0.36	0.234
Triglycerides (mmol l^−1^)	−0.18	−0.32	−0.04	0.07	−0.06	0.20	−0.25	0.08	−0.42	−0.09	0.004
C-reactive protein (mg l^−1^)	−0.80	−1.90	0.31	−0.38	−1.44	0.69	−0.42	0.64	−1.72	0.88	0.519
PFS food available	−0.41	−0.84	0.03	−0.12	−0.56	0.31	−0.28	0.18	−0.65	0.08	0.125
PFS food present	−0.67	−1.15	−0.19	−0.38	−0.86	0.10	−0.29	0.18	−0.66	0.08	0.122
PFS food tasted	−0.40	−0.79	−0.01	−0.21	−0.61	0.18	−0.19	0.17	−0.53	0.15	0.275
PFS total	−0.50	−0.88	−0.11	−0.24	−0.63	0.15	−0.25	0.14	−0.55	0.04	0.089
CoEQ craving control	23.81	12.52	35.09	12.13	0.76	23.49	11.68	4.74	2.07	21.28	0.019
CoEQ craving for sweet	−9.67	−16.54	−2.81	−2.70	−9.61	4.21	−6.97	3.52	−14.10	0.16	0.055
CoEQ craving for savory	−13.39	−21.57	−5.22	−2.94	−11.16	5.28	−10.46	4.12	−18.80	−2.12	0.015
CoEQ positive mood	−1.23	−9.10	6.64	2.60	−5.33	10.52	−3.83	3.13	−10.18	2.52	0.229
CoEQ control over craved nominated food	−28.44	−45.41	−11.47	−14.67	−31.76	2.41	−13.77	6.35	−26.66	−0.88	0.037

Estimated marginal means and 95% CIs computed from mixed-effects models adjusted for randomization arm and nightshift status, with an interaction term for diet and randomization arm and a random effect for participant.

#### Anthropometrics

Weight and BMI were significantly lower at 8 weeks from baseline on both diets. Waist circumference and waist-to-height ratio did not differ significantly. Reductions in weight (−0.96 kg (s.e., 0.40); *P* = 0.019) and BMI (−0.34 kg m^−2^ (s.e., 0.14); *P* = 0.021) were significantly greater on the MPF versus UPF diet, with no significant differences in waist circumference.

#### Body composition

Fat mass, body fat percentage, visceral fat rating and total body water mass were significantly lower at 8 weeks from baseline on the MPF but not UPF diet. Muscle mass, bone mass, fat-free mass and total body water percentage did not significantly differ at 8 weeks from baseline on either diet. Reductions in fat mass (−0.98 kg (s.e., 0.32); *P* = 0.004), body fat percentage (−0.76% (s.e., 0.28); *P* = 0.010), visceral fat rating (−0.41 (s.e., 0.15); *P* = 0.008) and total body water mass (−0.51 kg (s.e., 0.15); *P* = 0.002) were significantly greater on the MPF compared with UPF diet. Other body composition outcomes did not differ significantly between diets.

#### Heart rate and blood pressure

Systolic blood pressure (BP) (SBP) and diastolic blood pressure (DBP) were significantly lower at 8 weeks compared with baseline on the MPF but not UPF diet, whereas heart rate (HR) was significantly lower on the UPF but not MPF diet. Changes in BP and HR did not differ significantly between diets.

#### Clinical markers

Total cholesterol, high-density lipoprotein cholesterol (HDL-C) and non-HDL-C were significantly lower at 8 weeks compared with baseline on both diets. Glycated hemoglobin (HbA1c) and triglycerides were significantly lower at 8 weeks compared with baseline on the MPF diet only, whereas fasting glucose and low-density lipoprotein cholesterol (LDL-C) were significantly lower at 8 weeks compared with baseline on the UPF diet only. Bilirubin, alkaline phosphatase, alanine transaminase, albumin, total-cholesterol-to-HDL ratio and C-reactive protein (CRP) did not differ significantly at 8 weeks compared with baseline on either diet. Changes in triglycerides were significantly lower on the MPF than UPF diet (−0.25 mmol l^−1^ (s.e., 0.08); *P* = 0.004), whereas changes in LDL-C were significantly lower on the UPF than MPF diet (0.25 mmol l^−1^ (s.e., 0.10); *P* = 0.016). Changes in other biomarkers did not differ significantly.

### Subjective appetite measures

The Power of Food Scale (PFS) (food present, tasted and total score) and Control of Eating Questionnaire (CoEQ) craving for sweet, savory and difficulty to resist craved nominated food were significantly lower at 8 weeks from baseline on the MPF but not UPF diet. CoEQ craving control was significantly higher at 8 weeks on both diets. PFS food available and CoEQ positive mood at 8 weeks did not differ significantly from baseline on either diet. Improvements in CoEQ craving for savory (−10.46 (s.e., 4.12); *P* = 0.015), difficulty to resist craved nominated food (−13.77 (s.e., 6.35); *P* = 0.037) and craving control (11.68 (s.e., 4.74); *P* = 0.019) were significantly greater on the MPF than UPF diet. Other changes in CoEQ and PFS did not differ significantly between diets.

**Fig. 3:**
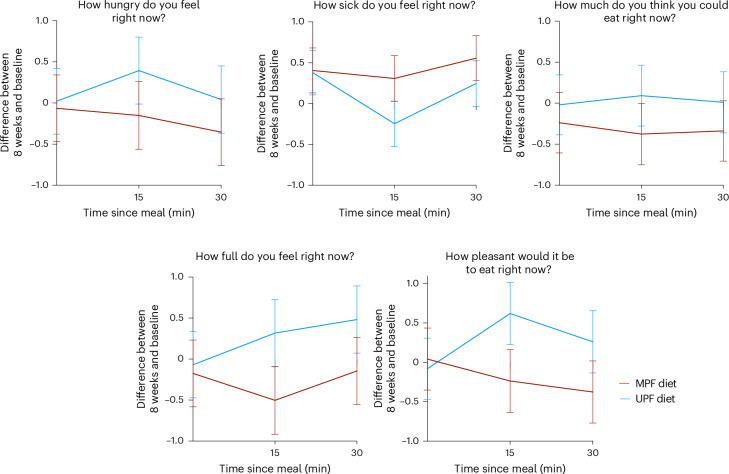
Changes in fasted and fed subjective appetite VAS at week 8 from baseline on MPF and UPF diets. Estimated marginal means and s.e. computed from mixed-effects models adjusted for randomization arm and nightshift status, with an interaction term for diet and randomization arm, interaction term for diet and time at visit (0, 15, 30 min) and a random effect for participant; ITT *N* = 50. Minute 0, fasted; minutes 15 and 30, fed.

Fasted and fed visual analog scales (VAS) did not significantly differ at 8 weeks from baseline on either diet, and changes were not significantly different between diets (Supplementary Tables 7 and 8). Trends were observed for lower postprandial hunger, capacity to eat, pleasure to eat and feelings of fullness on the MPF versus UPF diet (Fig. 3).

#### Diet intake and adherence

Self-reported diet intake data from the MPF and UPF diets are in Supplementary Tables 9–13. In the ITT sample, energy intake was −503.7 kcal day^−1^ (s.e., 130.2; *P* < 0.001) and −289.6 kcal day^−1^ (s.e., 102.8; *P* = 0.007) lower during the MPF and UPF diet compared with baseline, respectively. Self-reported energy intake was significantly lower (−327.3 kcal day^−1^ (s.e., 110.2; *P* = 0.005) on the MPF versus UPF diet.

In the ITT sample (*N* = 50), 32 and 35 participants provided completed food diaries for MPF and UPF diets, respectively. MPF adherence was 84.5% and UPF adherence was 91.2%. Adherence was higher in the first periods (MPF first, 91.8%; second, 78.5%; UPF first, 93.3%, second, 87.4%).

Diet ratings are listed in Supplementary Tables 14 and 15 (by randomization arm). There were no significant differences in ratings of the diets overall, meals and snacks overall, textures, portion sizes, hunger levels, contentment and sustainability. Flavors and tastes (−1.08 (95% CI, −1.99, −0.16); *P* = 0.022) and delivery and preparation (−1.60 (95% CI, −2.78, −0.42); *P* = 0.009) were rated significantly lower on the MPF versus UPF diet.

#### Physical activity

There were no significant differences in the change in moderate-to-vigorous physical activity (MVPA) performed from baseline to week 8 between diets (Supplementary Table 16).

### Safety

Adverse events (AE) are listed in Table 3. AEs were common and mild on both diets, with no related serious AEs (SAEs). Gastrointestinal AEs were most common. AEs did not differ significantly by diet (*P* = 0.088) or randomization arm (*P* = 0.390). Notably, greater AEs were reported on the UPF diet for constipation (MPF, 3; UPF, 11), dyspepsia/gastroesophageal reflux (MPF, 29; UPF, 36), fatigue (MPF, 4; UPF, 16), sleep-related AEs (MPF, 1; UPF, 7) and infections (MPF, 9; UPF, 17).

**Table 3:** AEs by diet and randomization arm

ITT *N* = 50	Frequency by diet			Frequency by randomization arm			Preintervention/washout
	MPF	UPF	*P* value	MPF/UPF	UPF/MPF	*P* value	
Participants reporting no AE	14	11		2	1		38
Gastrointestinal^a^			0.229			0.142	
Vomiting/nausea	2	2		0	4		
Constipation	3	11		7	7		1
Diarrhea (Loose stools, Frequent stools)	9	7		4	12		
Dyspepsia/gastroesophageal reflux^b^	29	36		29	36		
Gastritis	0	0		0	0		1
Abdominal pain	1	0		1	0		
General			0.385			0.465	
Fatigue (brain fog, tiredness)	4	16		8	12		1
Sleep related (sleepiness, poor sleep)	1	7		3	5		
Headache (including migraine)	2	2		2	2		
Dizziness (faint spells, light-headedness, vertigo)	3	4		4	3		1
Forgetfulness	1	0		0	1		
Mood change/low mood	4	3		6	1		
Acne	2	4		2	4		
Musculoskeletal^c^	5	7		5	7		1
Dental^d^	1	3		3	1		1
Cardio-renal-metabolic			1.000			1.000	
Elevated alanine transaminase	1	0		0	1		
Mild hepatic steatosis/fatty liver	1	0		1	0		
Kidney stone	1	0		1	0		
Hematuria	1	0		1	0		
Infection			0.739			0.686	
COVID-19/viral infection/cold/gastroenteritis	8	15		7	16		6
Cellulitis	0	1		1	0		
Scalp inflammation (history of alopecia)	1	0		0	1		
Other infection	0	1		0	1		
Other			0.236			0.746	
Nocturia	0	2		1	1		
Hot flushes	1	0		0	1		
Premenstrual syndrome/premenstrual dysphoric disorder	1	0		0	1		
Body odor change	0	1		1	0		
Cough	0	0		0	0		1
Hand burn	0	1		0	1		1
Spider bite	1	0		0	1		
Shingles	0	0		0	0		1
Puffy face	0	2		2	0		
Panic attack	0	1		0	1		
Hospitalization (unknown reason), unrelated SAE	1	0		0	1		
Totals			0.088			0.390	
Gastrointestinal	44	56		41	59		2
General	23	46		33	36		4
Cardio-renal-metabolic	4	0		3	1		0
Infection	9	17		8	18		6
Other	4	7		4	7		3
Total count	84	126		89	121		15

Statistical comparisons using Fisher’s exact test.

^a^Gastrointestinal adverse events: two vomiting/nausea, one diarrhea, one dyspepsia/gastresophageal reflux on the MPF diet and one vomiting/nausea, one diarrhea on the UPF diet were not related to the intervention.

^b^Dyspepsia/gastroesophageal reflux includes heartburn/acid reflux, stomach discomfort, flatulence, bloating, belching, dry heaving and indigestion.

^c^Musculoskeletal AEs include muscle cramp, muscle weakness, joint pain, back pain, plantar fasciitis and Achilles tendonitis.

^d^Dental AEs include dental pain, dental implant infection, gingivitis, caries and cracked tooth. Gastrointestinal adverse events: two vomiting/nausea, one diarrhea, one dyspepsia/gastresophageal reflux on the MPF diet and one vomiting/nausea, one diarrhea on the UPF diet were not related to the intervention. Statistical comparisons using Fisher’s exact test.

### Exploratory outcomes

Changes in body composition corresponded to estimated daily energy imbalances of −289.9 kcal day^−1^ (95% CI, −423.7, −156.1) and −119.5 kcal day^−1^ (95% CI, −251.7, 12.7) on MPF and UPF diets, respectively, which was significantly lower on the MPF diet (−170.4 kcal day^−1^ (s.e., 57.9); *P* = 0.005). Maintaining the 8-week weight loss trajectories over 1 year would be estimated to result in ~9% and ~13% weight loss on the MPF diet, and ~5% and ~4% weight loss on the UPF diet, for female and male participants, respectively. Habitual UPF intake and initial weight were not significantly associated with %WC on either diet. Habitual energy intake was inversely associated with %WC on the MPF (*P* = 0.033) but not UPF diet (*P* = 0.090).

Changes in waist-to-height ratio at 8 weeks from baseline did not differ significantly between diets (Supplementary Table 17).

### Sensitivity analyses

In prespecified sensitivity analyses, results were consistent at 4 weeks (Supplementary Table 18), in repeated-measures analyses (Supplementary Table 19), in PP analyses (Supplementary Tables 8 and 20–22) and in sensitivity analyses using inverse probability weighting and multiple imputation (Supplementary Table 23). In post hoc sensitivity analyses, results were consistent when analyzing primary outcome data only where participants provided diet adherence data (Supplementary Table 23), and when using first-period diet data only, except that BP changes were significantly lower on the MPF versus UPF diet, and fasting glucose change was no longer significantly different between diets (Supplementary Tables 6 and 8).

## Discussion

In this study, both MPF and UPF diets following national healthy dietary guidance resulted in percentage weight loss after 8 weeks, with significantly greater reductions on the MPF diet. Greater weight, BMI and fat mass loss were also observed on the MPF compared with the UPF diet, as well as greater reductions in triglycerides and cravings. Conversely, LDL-C was lower on the UPF diet. Overall, these results suggest favorable changes in body composition and craving control from adhering to national dietary guidance with a diet of MPF rather than UPF.

These findings build on two metabolic ward RCTs assessing the health impacts of ad libitum UPF and MPF/non-UPF diets matched for presented calories and nutrients, highlighting the importance of UPF in addition to traditional dietary guidance. Hall et al. observed weight loss on a 2-week MPF diet (−0.9 kg) and weight gain on a 2-week UPF diet (0.9 kg)^20^, whereas Hamano et al. reported weight gain on both 7-day non-UPF and UPF diets, but with significantly greater weight gain (1.1 kg (95% CI, 0.2, 2.0)) on the UPF diet^21^. In contrast with our hypothesis given the body of observational evidence linking UPF with weight gain^8^, the UPF diet following UK dietary guidance resulted in weight loss. However, weight loss on the MPF diet was significantly greater than on the UPF diet. Our study therefore confirms and builds upon previous findings, showing significant differences in weight change between matched UPF and MPF/non-UPF diets^20,21^, within the context of existing healthy dietary guidance.

Previous trials further considered changes in body composition. Hall et al. found that fat mass increased on the UPF diet, but decreased on the MPF diet, differing significantly between diets^20^. Similar findings were reported in Hamano et al.^21^, with additional findings of no liver fat changes. Regarding fat-free mass, Hall et al. observed trends for an increase on the UPF diet and decrease on the MPF diet, which again showed differences^20^, whereas Hamano et al. saw no significant differences between diets^21^. Our study builds upon these findings by considering body composition changes from UPF and MPF diets in the context of dietary guidance. We observed that the greater weight loss on the MPF diet was through greater reductions in fat mass and total body water mass, with no significant differences in fat-free mass change between diets. Despite the UPF diet leading to weight loss, there were no significant reductions in adiposity (fat mass, body fat percentage or visceral fat rating), with the greater fat mass reductions on the MPF diet being key in addressing obesity-related poor cardiometabolic health^23^. However, no significant differences were observed for waist circumference change between diets.

Clinically significant weight loss is related directly to favorable changes in cardiometabolic risk factors, including BP, blood glucose, HbA1c and lipids^24^. However, the greater weight and fat mass reductions on the MPF compared with the UPF diet did not translate into significant improvements in cardiometabolic risk factors over the UPF diet, except triglycerides. Indeed, the UPF diet led to reductions in several cardiometabolic risk factors including HR, fasting glucose, cholesterol and LDL-C. Whereas only the MPF diet resulted in significant BP reduction, this did not differ significantly from the UPF diet. Similarly, limited differences in biomarkers were observed previously. Hall et al. reported lower HDL-C on the MPF versus UPF diet, but no significant differences in triglycerides, high-sensitivity CRP, HbA1c, glucose or LDL-C between diets^20^. Hamano et al. reported significantly greater reductions in total cholesterol and HDL-C on the non-UPF versus UPF diet, and significantly greater increases in liver function markers on the UPF versus non-UPF diet, but no differences in other markers. For our trial and the two previous trials, longer durations may be required for sufficient weight loss differences to occur between diets for clinically significant differences in cardiometabolic risk factors to emerge.

Several mechanisms are proposed for the contrasting weight changes between UPF and MPF diets, including nutrient composition, texture, energy density and eating rate^8^. In contrast to the two previous trials^20,21^ and to typical nutrient-poor UPF diets^25^, the UPF diet in this study followed national healthy dietary guidance. This included nutritionally improved, reformulated UPF such as breakfast cereals, ready meals and plant-based alternatives. These typically have nutrition or health claims and green and amber front-of-package label traffic lights (which guide consumer choice at point-of-purchase, with red traffic lights for products high in fat, saturated fat, sugar and/or salt)^25^. Such foods are nutritionally comparable to MPF in the UK^25^ and recommended in current UK dietary guidelines^16^. Thus, the presented UPF diet contained recommended intakes of nutrients, fiber and fruit and vegetables^16^. Participants’ habitual diets were typically misaligned with EWG recommendations and were above average for UPF consumption in the UK^6^. Therefore, the improvement in diet quality towards EWG recommendations on the provided UPF diet without necessarily increasing UPF intake from participants’ habitual diets likely explained the neutral or favorable changes and the absence of detrimental changes on the UPF diet. However, despite this, the UPF diet did not result in the same extent of weight loss as the MPF diet, nor did it result in significant fat loss. Reported energy intakes were in line with the primary outcome findings, with a deficit on both diets, but to a greater extent on the MPF diet. Removing UPF provided additional benefit beyond existing dietary recommendations, suggesting other potential mechanisms of UPF besides nutritional quality.

One potential mechanism is energy density. The UPF diet in this trial was more energy dense than the MPF diet. This is representative of nutritionally improved ‘healthy’ UPF in the UK, which have a higher energy density than comparable ‘healthy’ MPF^25^. In previous RCTs, nonbeverage energy density of the UPF diet was also higher, with a faster eating rate (both energy and weight)^20^ and fewer chews per bite on the UPF diet^21^, which can promote greater energy intake^26^. Hyperpalatability and taste may also promote increased UPF consumption^27^. Both previous trials reported similar appetite ratings between diets^20^. Similarly, we found comparable ratings between diets. However, flavor and taste ratings were significantly lower on the MPF diet. This may have impacted on eating behavior and led to lower consumption on the MPF diet or greater consumption on the UPF diet, as evidenced with withdrawals on the MPF diet but no withdrawals on the UPF diet. There were also improvements in craving control on the MPF versus UPF diet despite greater weight loss, as well as significant reductions in hedonic appetite on the MPF but not UPF diet, although differences were not significant. There were also trends favoring improved subjective appetite in the immediate postprandial state on the MPF diet. Combined, these findings may promote appropriate meal termination on the MPF versus UPF diet, reflected in the greater weight loss, and further supporting long-term weight loss maintenance^28^. Marketing and advertising heavily influence eating behavior^29^, particularly UPF^30^. Previous trials were conducted in metabolic wards, providing UPF without packaging. In this study, UPF were delivered in their branded packaging as experienced in the real world. Although no products on the UPF diet included ‘reduced calorie’ labeling, many carried nutrition or health claims. This may have influenced eating behavior and perceptions of appropriate portion sizing^31^, eating the suggested UPF portion sizes compared with eating ad libitum on the MPF diet.

Although no significant differences in AEs were observed between diets, a higher incidence of fatigue and common gastrointestinal issues including constipation and dyspepsia/reflux were observed on the UPF diet. Growing evidence links UPF intake with gastrointestinal pathology, including inflammatory bowel disease, colorectal cancer and disorders of gut–brain interaction^32^. This study provides preliminary experimental insights into potential gut disruption from UPF that requires further investigation.

An order effect was observed, with less weight loss on the second-period diets across both randomization arms. Hall et al. observed no significant order effects between diets on energy intake, bodyweight or body composition, despite no washout period^33^. Adherence was likely a factor explaining the lower %WC during second-period diets here, with a notable drop in adherence on the second-period MPF diet and, to a lesser extent, the second-period UPF diet. Furthermore, simultaneously and instantaneously switching onto a healthy diet devoid of ultraprocessing may have had an additive effect on %WC compared with a more modest effect from switching onto a healthy diet that remained high in UPF. The subsequent transition onto the healthy diet devoid of ultraprocessing may have had attenuated effects on %WC. Results were consistent when analyzing data from the first-period diets only, with significant differences of 1.86% in %WC between diets.

These findings highlight the benefit of following UK dietary recommendations. Maintaining the 8-week weight loss trajectories over 1 year may result in an estimated 9–13% and 4–5% weight loss on MPF and UPF diets, respectively^34^. However, this trial also demonstrates the differential impact of UPF and MPF on weight and body composition while following national dietary guidance, and the obesity-related health implications of the foods constituting most of the energy intake in the UK population^6^.

These findings on the relative importance of food processing should complement and expand, not displace, current understanding of diet-related health. A House of Lords committee report highlighted the need for a healthier UK food environment^35^. The wider food system is key in driving diet-related poor health and obesity by enabling ready availability to cheap, unhealthy food^36^. Little success^13^ has been achieved in addressing obesity since 1992, despite 14 government obesity strategies in England^37^. Many obesity policies focus on person-level actions, rather than system-level changes. The concept of Nova and ultraprocessing shifts the focus onto the environmental drivers of obesity and the influence of transnational food companies in shaping unhealthy food environments^36,38^. Reductionist approaches focusing solely on nutrient reformulation or individual-level action insufficiently address environmental factors. Stakeholders across disciplines and organizations must align and focus on wider actions to improve the food environment (for example, taxes and subsidies), to enable affordable, available and desirable healthy diets for all^8,36^.

Strengths of UPDATE include the 8-week duration of each intervention. Diets were matched for UK national dietary guidance, ensuring results are directly relevant for UK public health food policy. Given the similarities between UK and most dietary guidelines worldwide that do not consider UPF, these findings are likely to be relevant to many countries. The crossover design removed between-participant confounding, and the free-living, community-based setting provides real-world evidence directly applicable to the public. Participants were blind to the primary outcome and not told to change their weight or dietary intake. Providing all food and drink ready prepared without cost to participants’ homes helped maximize adherence, ensure internal validity and minimize dropout^39,40^. UPF was sourced from leading UK supermarkets and were not culinary preparations, providing a diet representative of UPF available in the UK. Participants’ habitual dietary intakes were broadly similar to UK averages^41^, aiding generalizability to the UK population. The trial was funded by a medical charity and nongovernmental organization, without industry or commercial influence.

Limitations include that a potential carryover effect cannot be ruled out. However, the washout period helped minimize this. People with dietary restrictions (for example, vegan, halal, kosher) were excluded due to financial and logistical constraints, limiting generalizability. However, participants with minor dietary restrictions/intolerances were eligible if such foods were not on menus. The results also may not generalize to people with low UPF intake, and do not imply that switching from a low- to high-UPF diet following dietary guidance carries neutral or favorable effects. The lack of inpatient settings limits monitoring of adherence. Not all participants returned their food diaries to monitor adherence. However, reported adherence was high, with previous studies indicating high adherence when all food is provided and delivered to participants’ homes^39^. Moreover, results were unchanged when considering only the sample returning food diaries. It was not possible to directly assess energy balance measures such as energy intake or mechanisms such as eating rate due to the free-living design. To minimize participant burden, nutrient biomarkers and stable isotope analyses were not used for diet assessment; however, there are currently no validated biomarkers of UPF intake. Detailed body composition analyses such as dual X-ray absorptiometry were also not conducted. Finally, there was no processed food diet, though the smaller number and range of processed foods limits the ability to create a healthy, balanced processed food diet^25^.

In conclusion, ad libitum 8-week MPF and UPF diets meeting UK dietary guidance resulted in weight loss, but with significantly greater reductions in weight on the MPF than on the UPF diet. These findings highlight the importance of food processing in public health policy and dietary guidance in addition to existing recommendations.

## Methods

The trial protocol has been published previously^22^. Standard Protocol Items: Recommendations for Interventional Trials (SPIRIT) guidelines^42^ were used to design the protocol, and reporting was according to the Consolidated Standards of Reporting Trials (CONSORT)^43^.

### Participants and setting

Fifty-five adults were recruited from South East England and London. Written informed consent was obtained before any screening or research-associated measurement. The last participant last visit was 13 October 2024.

### Eligibility criteria

Inclusion criteria included any staff at University College London Hospital (UCLH), age ≥18 years, BMI between ≥25 kg m^−2^ and <40 kg m^−2^ (living with overweight or obesity), ≥50% kcal day^−1^ of habitual dietary intake consisting of UPF, weight stable (≤5% variation in weight in the last 3 months), medically safe to participate in a dietary intervention, able to read and write in English, willing and able to give written informed consent, able to comply with the study protocol and attend relevant inperson and online sessions and use of contraception until the end of the intervention period where necessary. Exclusion criteria included contraindication for a dietary intervention, participation in another clinical intervention trial, BMI > 40 kg m^−2^ or basal metabolic rate ≥2,300 kcal day^−1^ (to ensure intervention diets are at least 300 kcal day^−1^ greater than maintenance energy needs, based on excess energy intakes reported in ref. ^20^), diagnosis of type 2 diabetes or use of insulin, eating disorder, celiac disease or inflammatory bowel disease, any dietary restrictions (for example, vegan, vegetarian, Halal or kosher requirements, diagnosed food allergy or other allergy) that limit the ability to adhere to the dietary intervention, recent commencement of medications that cause weight gain or weight loss, a history of drug or alcohol abuse, pregnancy, breast-feeding or intention to become pregnant and any other factor making the participant unsuitable in the view of investigator.

### Changes to protocol

On 16 June 2023, the UPF intake inclusion criterion was lowered from ≥60% to ≥50% to better reflect average UPF intake of prospective participants in South East England and London^6^. Participants were also asked to provide ratings of both diets after completion of the RCT (detailed below).

### Randomization

Participants were block randomized by the research team using Sealed Envelope (https://www.sealedenvelope.com) to either (1) the MPF diet then UPF diet (*n* = 28), or (2) the UPF diet then MPF diet (*n* = 27). Sealed Envelope generated the random allocation sequence. Randomization was stratified by nightshift status, sex and ethnicity. Researchers were not blind to assignment and enrolled participants. An independent statistician verified the primary outcome analysis while remaining blind to allocation assignment. Participants were not informed of the processing groups of the diets. All participant communications omitted the terms MPF or UPF, with diets being referred to as Diet A or Diet B.

### Intervention

Participants were provided with an 8-week MPF diet and an 8-week UPF diet, both following EWG recommendations, in a random order, with a 4-week washout period. Participants were given all meals, snacks and drinks for both diets, which were delivered to participants’ homes twice per week. The Nova classification was used to classify food and drink into UPF and MPF^5^. The research team agreed on UPF items based on identifying ingredients of industrial use in product ingredient lists explicitly defining a product as UPF in published definitions (for example, cosmetic additives)^5^. Meals and snacks on the MPF diet were culinary preparations of individual ingredients (for example, raw meat, vegetables, oats, butter) ensuring correct Nova classification and no ambiguous decision on mixed dishes/shop-bought items.

Diets were matched for, and followed, government recommended nutrient intakes in the EWG^15,16^, which focuses on specific macronutrients and food types. Guidance includes choosing foods lower in saturated fat, added sugar and salt, consuming five daily portions of fruit and vegetables, basing meals on starchy carbohydrates and eating a variety of foods in the right proportions^16^ (Supplementary Table 24). To ensure ad libitum energy intake, diets were scaled up to approximately 4,000 kcal day^−1^. Menus were designed to be representative of UK diets by identifying the most commonly consumed food groups from the UK National Diet and Nutrition Survey^41^. Practical and logistical aspects including price, best-before dates, storage and preparation requirements, and accessibility were factored into the design^44^. Meals and snacks were matched across diets where possible, with a 7-day rotating menu to prevent participant boredom and sensory-specific satiety^45^. A patient and public involvement focus group provided feedback on the menu before the study. Menu guides were provided with instructions and pictures to prepare each meal. Supplementary Tables 24–26 report the average nutrient compositions of the provided diets, the menus and the images of meals and snacks on the menus, respectively.

As in previous ad libitum feeding trials investigating weight change^46^, participants were asked to consume as much or as little of the provided diets as desired. Participants were told to consume only the food and drink provided and to not consume any other food or drink, except water, during each 8-week diet. Tea and coffee were provided. Minor modifications to the intervention that did not alter the overall design were acceptable for enabling adherence (for example participants were allowed to add additional herbs and spices to meals but were not allowed to use any calorie- or salt-containing condiments). Alcohol was allowed but not provided. Participants were told to keep alcohol consumption within government guidelines (≤14 weekly units)^47^. Participants were educated on the EWG, but no further lifestyle guidance was provided (that is, no advice on physical activity, smoking or sleep). Participants were supported during each diet through weekly calls with the research team to discuss any issues and to promote adherence. Participants returned to their habitual diet during the 4-week washout period to minimize carryover effects. No food was provided during the 2-week baseline periods.

### Procedures

Figure 1a outlines the study design and measurement timepoints. Age, sex, ethnicity, occupation, nightshift work pattern, educational level, marital status, medical history, medication intake, alcohol consumption, smoking habits and family history of obesity, cardiovascular disease and diabetes were self-reported at screening.

The baseline period lasted 2 weeks to allow time to collect all data to check eligibility, including regarding habitual UPF intake with two additional nonconsecutive recalls after screening, followed by randomization and booking in the baseline visit, and then sufficient notice and time to set participants up with their first food delivery. For consistency, a 2-week assessment window was used for the baseline visit of the second diet.

Weight was measured using an electronic scale to the nearest 0.1 kg (Tanita DC-430MAS; Tanita). Body composition, including fat mass, body fat percentage, visceral fat rating, fat-free mass, muscle mass, bone mass, total body water mass and total body water percentage were assessed using bioelectrical impedance analysis (BIA) (Tanita) at each visit. BIA at baseline and week 8 was conducted following an overnight fast with no alcohol intake or strenuous activity in the preceding 24 h. Assessments at week 4 were not fasted. Participants were provided with standardized wording in the week before their baseline and week 8 visits to maintain a consistent hydration status: “Please make sure that for the visit, you eat your usual diet for the 24 h before the visit day and to avoid alcohol and strenuous exercise. Please fast from 20:00 pm on the night before the study visit, and drink only water. Please do try to drink some water before the visit as this helps with the cannulation.ˮ Upon arrival, participants were asked to confirm that they had fasted and given the opportunity to drink water to thirst before measurements to ensure consistency. Basal metabolic rate was estimated by the Tanita BIA scanner based on fat-free mass and participant age. Height was assessed using a stadiometer to the nearest 0.5 cm. Waist circumference was measured in centimeters using an inelastic tape measure at the iliac crest^48^. BMI was derived from weight and height (in kg m^−2^), and waist-to-height ratio from WC and height. Estimated daily energy imbalance was assessed using the energy densities of fat mass and fat-free mass of ~9,300 kcal kg^−1^ and 1,100 kcal kg^−1^, respectively^49^. The mean daily energy imbalance (kcal day^−1^) for each participant for each diet was calculated as (9,300 × change in fat mass (kg) from baseline to week 8 + 1,100 × change in fat-free mass (kg) from baseline to week 8)/exact number of days from the start of the diet to the week 8 BIA assessment date. BP was recorded in triplicate, seated, alongside HR with an automated sphygmomanometer and oximeter. BP was recorded as the average of the second and third recordings. Venous blood samples were collected after an overnight fast and included glucose, HbA1c, liver function tests (bilirubin, alkaline phosphatase, alanine transaminase and albumin), lipids (total cholesterol, HDL-C, LDL-C, total-cholesterol-to-HDL ratio, non-HDL-C and triglycerides) and CRP.

CoEQ is a 21-item validated measure of the severity and type of food cravings that a person experiences, as well as of their inhibitory control of eating and subjective sensation of appetite and mood^50^. The CoEQ contains four domains: overall craving control, craving for sweet, craving for savory and positive mood and one question on perceived control over resisting a self-nominated craved food. PFS is a 15-item validated measure of hedonic appetite, food reward sensitivity and the psychological impact of living in food-abundant environments^51^. PFS assesses the appetite for and motivation to consume palatable foods at three levels: food available (but not physically present), food present (but not tasted) and food tasted (but not yet consumed)^52^. An overall PFS score is then computed from the mean of the three subscores. PFS and CoEQ were collected at baseline and at 4- and 8-week visits.

A 30-min meal test was used to assess acute changes in subjective appetite levels in the fasted and fed state at baseline and at 8 weeks. A five-item subjective appetite VAS was completed following an overnight fast. The questions capture aspects of hunger and the desire to eat: “How hungry do you feel right now?,ˮ “How sick do you feel right now?,ˮ “How much do you think you could eat right now?,ˮ “How full do you feel right now?ˮ and “How pleasant would it be to eat right now?,ˮ on a ten-point 100-mm scale, with words anchored at either end marking the extremes (“Not at allˮ and “Extremelyˮ)^53^. A liquid meal (187.5 ml Abbott Ensure (450 kcal, 17.5 g fat, 54.0 g carbohydrate, 19.1 g protein) was then consumed, and the subjective appetite VAS assessments were repeated at 15 and 30 min after starting the liquid meal.

Baseline habitual dietary intake was assessed using Intake24 (ref. ^54^)—a validated, online, self-reported 24-h recall system, based on a multiple-pass recall suitable for the general population (https://intake24.co.uk)^55,56^. Two nonconsecutive 24-h recalls were completed at screening, baseline and at week 4 and week 8 on each diet. Food diaries were provided to record adherence to the diets and report any foods consumed off diet. Nonadherence was prespecified as consuming more than one meal per week off the provided intervention diet. Participants were encouraged to report any deviations from the provided diets and to be as honest as possible, with no repercussions. All completed and returned food diaries were analyzed. The research team provided several options and opportunities for participants to return food diaries to maximize collection, including drop off at follow-up visits or at the research center at participants’ convenience during the trial. For any unreturned food diaries, participants were followed up several times to drop off food diaries at the research center at their convenience, post them at no cost or to email their food diary.

MVPA was measured objectively using wGT3X-BT (ActiGraph)—an accelerometer-based activity monitor providing information on body movement using a motion sensor. The device is a reliable tool and has been used widely in clinical research given its practicality, noninvasiveness, and accuracy in measuring physical activity levels in free-living adults^57^. Participants were instructed to wear the device on their dominant hip continuously for 7 days, to be removed only for water-based activities. Average daily MVPA is a validated measure obtained from hip-worn accelerometers^58^. For data to be valid, participants must wear the device for at least 4 days with at least 10 h of daily wear time. Wear time was validated in ActiGraph ActiLife software (v.6.13.6), based on the criteria in ref. ^59^. Thereafter, the cut points proposed by Freedson et al.^58^ were applied to each participant’s counts per minute data to derive the length of time spent in sedentary, light, moderate, vigorous and very vigorous physical activity to calculate average daily MVPA.

Following completion of the RCT, participants were asked to rate both diets on a scale of 0–10, with 0 indicating a negative, poor or bland experience, or the least intensity of the attribute being evaluated, and 10 indicating a positive, excellent or flavorful experience, or the greatest intensity of the attribute being evaluated. Ratings were of the overall experience, of meals and snacks, of flavors and taste, of textures, of portion sizes, of delivery and required preparation, of hunger level, of happiness/contentment and of diet sustainability. Further details on the ratings are provided in Supplementary Table 27.

### Safety and AE monitoring

AEs were recorded by the clinical research team at baseline, at 4-week and 8-week study visits and from weekly phone calls. The assessment of the relationship of an AE with the intervention was carried out by the clinical research team. AEs were considered related if the causal relationship between the intervention and an AE was at least a reasonable possibility, that is, the relationship could not be ruled out. Reporting of AEs and serious AEs was conducted according to the Sponsor’s standard operating procedures, and updates on AEs were reported to the Trial Steering Committee and Trial Management Group. All SAEs were reported to the Sponsor within 24 h of the clinical research team becoming aware. A Data and Safety Monitoring Committee was not set up as no SAEs or notable risks were expected from participation. Incidental findings were reported to participants and their general practitioner as per written informed consent.

### Outcomes

The primary outcome was the within-participant difference in %WC between MPF and UPF diets at 8 weeks from baseline. %WC is currently used clinically in weight management clinics and across all NHS weight management programs.

Prespecified secondary outcomes include changes in weight, waist circumference, BMI, body fat percentage, fat mass, fat-free mass, visceral fat rating, muscle mass, bone mass, total body water mass, total body water percentage, HR, SBP, DBP, blood markers (HbA1c, glucose, liver function tests, lipids and CRP), PFS (food available, food present, food tasted and total score), CoEQ (overall craving control, craving for sweet, craving for savory, positive mood, perceived control over resisting a self-nominated craved food), fasted and fed changes in the five-item subjective appetite VAS, dietary intake and average daily MVPA. Secondary outcomes of brain magnetic resonance imaging functional resting-state connectivity, physical function, sleep quality, mental health, quality of life and metabolomics, and results from the follow-up 6-month behavioral support program^22^ will be reported separately.

Nonprespecified outcomes included AEs, changes in waist-to-height ratio, estimated daily energy imbalance and differences in post hoc ratings of each diet.

### Power calculation and sample size

The sample size is based on estimated within-participant variation^22^. The expected weight loss trajectory over 8 weeks was modeled using the National Institutes of Health bodyweight planner^34^ (https://www.niddk.nih.gov/bwp) and based on data from Hall et al. showing 0.9 kg weight loss following a 2-week MPF diet^20^, with a s.d. of the mean difference in weight change between MPF and UPF diets of 1.98 kg (mean, 1.85 kg). In total, 44 participants were required to detect a mean difference of 2.7% WC between groups, assuming weight loss on the MPF diet and no WC on the UPF diet, with a s.d. of the mean difference of 5.4% (power, 0.9; alpha, 0.05; two-sided paired *t*-test, SPSS v.27.0). The final sample size was 55, factoring for a 20% dropout rate.

Normally distributed variables were reported using means and s.e., and non-normally distributed variables reported using medians and interquartile ranges. Categorical variables were described using frequencies and percentages, and analyzed using *χ*^2^ tests or Fisher’s exact tests where appropriate. Values are presented by randomization group and analyzed as randomized.

The primary outcome analysis was prespecified as an ITT analysis, with all available data being analyzed as randomized. Any participants were included regardless of dropout status if follow-up data on the primary outcome was observed. To use all available data, any participants withdrawing from the trial were included in the ITT analysis if they provided follow-up primary outcome data for at least one of the diet interventions (that is, the minimum data required to contribute to the primary analysis). This includes participants who dropped out after completing the first-period 8-week assessment (use of the first-period data only), or participants who dropped out in the first or second period before the 8-week assessment and agreed to attend 8-week follow-up assessments for measurement of weight (use of the first- and second-period data).

The ITT analysis included participants with baseline and week 8 primary outcome values for at least one diet. The PP analysis included participants with baseline and week 8 values for both diets, and no withdrawal.

### Statistical analysis

#### Primary analysis

Mixed-effects models were used in an ITT analysis to assess the difference in %WC at 8 weeks and secondary outcomes, with a random effect for participants, and adjusting for randomization arm (including interaction with diet) and nightshift status. The primary outcome effect size with 95% CIs was then computed from the mixed-effects model using Cohen’s *d*^60^.

Initially, an interaction term was included in the primary outcome analysis mixed-effects model between the treatment (UPF or MPF diet) and the randomization arm (MPF/UPF or UPF/MPF) to assess any potential treatment-by-period or carryover effect. This interaction was significant (*P* < 0.001) and included in the mixed-effects models to account for the diet order/sequence.

The criteria used for selecting potential adjustment covariates for the mixed-effects model were prespecified in the protocol. These were defined as the randomization stratification variables (sex, ethnicity and nightshift status selected a priori based on the literature^61–63^), as well as any baseline participant variables that were not balanced between randomization arms.

Each potential participant baseline adjustment confounder identified from the prespecified criteria was added to the base mixed-effects model (the effect of diet adjusted for randomization arm, with an interaction term for diet and randomization arm and a random effect for participant). The significance of each confounder and the impact on the ITT primary outcome analysis effect estimate was assessed to determine inclusion in the final model (see Supplementary Table 23 for the model adjustment results). In this respect, sex, ethnicity and estimated baseline BMR were each individually not significant predictors of %WC in the mixed-effects model and did not alter the effect estimate of the ITT primary outcome analysis (see Supplementary Table 23 for the model adjustment results). These potential confounders were therefore not included in the final model. In contrast, nightshift status, when added individually, to the based mixed-effects model was a significant predictor of %WC.

Mixed-effects model assumptions including normality of residuals and homoscedasticity were checked visually and verified.

#### Sensitivity analyses

Unadjusted analyses of primary and secondary outcomes at 8 weeks were compared with baseline, and differences in changes from baseline to 8 weeks between diets were assessed using paired *t*-tests. Analyses were repeated for changes in outcomes at week 4 from baseline between diets, for changes at week 4 and week 8 from baseline between diets using repeated-measures mixed-effects models, for the PP sample, and for results using data from the first period of each randomization arm only. Carryover effects were not assessed as it is not possible to identify a carryover effect or adjust for it in a 2 × 2 crossover design^43^. No interim analysis was planned or conducted.

The impact of missing data on the primary outcome analysis was assessed using multiple imputation with chained equations under the assumption of data missing at random. Missing data for the primary outcome were first imputed using model variables (diet, randomization arm, nightshift status and available data for %WC), and then imputed using model variables and auxiliary baseline variables (diet, randomization arm, nightshift status, available data for %WC, ethnicity, sex, occupation, education, family history of obesity, baseline estimated BMR, baseline energy intake and baseline weight). The impact of missing data on the primary outcome analysis was also assessed using inverse probability weighting. Propensity scores for receiving the treatment (MPF diet or UPF diet) were first calculated using the randomization stratification variables: sex, ethnicity and nightshift status, and baseline estimated BMR, and then calculated using randomization stratification variables and auxiliary baseline variables (randomization arm, nightshift status, available data for %WC, ethnicity, sex, occupation, education, family history of obesity, baseline estimated BMR, baseline energy intake and baseline weight). Stabilized weights were then used to reweight the remaining sample.

Analyses were conducted in R v.2024.04.1+748. Data were presented in tabular form using Microsoft Excel v.16.91 (24111020), figures were created using Prism 10 v.10.2.3. Statistical significance was set at *P* < 0.05. As secondary outcomes in this study are exploratory in nature, significance values were not adjusted for multiple comparisons. Any apparent significance of these results should be confirmed in future research.

### Ethics

The Yorkshire and The Humber–Sheffield Research Ethics Committee approved the trial on 22 December 2022 (22/YH/0281). The study was registered prospectively on ClinicalTrials.gov (NCT05627570). All participants provided written informed consent before any screening or research-associated measurement.

### Patient and public involvement

NHS staff at UCLH provided input to the trial design following a focus group session. Obesity Empowerment Network UK members with lived experience of obesity also contributed to the study design. One member of the trial steering committee was a lay person. Participants could consent to a lay summary of the trial results.

### Reporting summary

Further information on research design is available in the Nature Portfolio Reporting Summary linked to this article.

## Online content

Any methods, additional references, Nature Portfolio reporting summaries, source data, extended data, supplementary information, acknowledgements, peer review information; details of author contributions and competing interests; and statements of data and code availability are available at 10.1038/s41591-025-03842-0.

## Supplementary information

Supplementary Information Supplementary Tables 1–27.

Reporting Summary

## Data Availability

UCL is the data controller for the data in this study. Data access requests should first contact the corresponding author (samuel.dicken.20@ucl.ac.uk) to discuss data of interest and to obtain approval. Data for all outcomes in this paper can be requested. Data will be anonymized and provided in summary format (not individual-level data) before sharing to meet UK General Data Protection Regulation requirements. The timeline between requesting data and approval of data requests is 3 months. Data will be provided within 3 months of approval.

## Code availability

Code for the analysis is publicly available and available without restriction via GitHub at https://github.com/SamuelJDicken/UPDATE.
