# Supplementary material for: Ultraprocessed or minimally processed diets following healthy dietary guidelines on weight and cardiometabolic health: a randomized, crossover trial
Source: Nat Med. 2025 Aug 4;31(10):3297–308. doi: 10.1038/s41591-025-03842-0 (PMC12532614; doi:10.1038/s41591-025-03842-0)
Supplement: Supplementary file 1 — Supplementary Tables 1–27. [file 41591_2025_3842_MOESM1_ESM.pdf]

nature medicine

Supplementary information https://doi.org/10.1038/s41591-025-03842-0

Ultraprocessed or minimally processed diets following healthy dietary guidelines on weight and cardiometabolic health: a randomized, crossover trial

In the format provided by the authors and unedited

Supplementary Materials

Supplementary Tables

1. Supplementary Table 1: ITT and PP: Baseline habitual dietary intakes overall and by randomisation arm
2. Supplementary Table 2: ITT: Baseline demographic and clinical characteristics overall and by randomisation arm.
3. Supplementary Table 3: PP: Baseline demographic and clinical characteristics overall and by randomisation arm.
4. Supplementary Table 4: Baseline demographic, clinical and dietary characteristics by dropouts versus non-dropouts.
5. Supplementary Table 5: ITT: Unadjusted changes in outcomes from baseline to week 8 on each diet, and differences in changes in outcomes from baseline to week 8 between diets.
6. Supplementary Table 6: ITT: Unadjusted changes in outcomes from baseline to week 8 on each diet by randomisation arm, and unadjusted and adjusted differences in changes in outcomes from baseline to week 8 between first diets.
7. Supplementary Table 7: ITT: Unadjusted and adjusted changes in fasted and fed subjective appetite visual analogue scales from baseline to week 8 on each diet.
8. Supplementary Table 8: Adjusted differences in fasted and fed subjective appetite visual analogue scales from baseline to week 8 for ITT, PP and for first diets only.
9. Supplementary Table 9: ITT: Unadjusted self-reported dietary intakes at baseline, week 4 and week 8 on each ad libitum diet.
10. Supplementary Table 10: Unadjusted self-reported dietary intakes on each ad libitum diet (week 4 and week 8 average) and unadjusted and adjusted differences in dietary intake between each ad libitum diet for ITT and PP.
11. Supplementary Table 11: ITT: Unadjusted changes in self-reported dietary intake from baseline to week 4 and week 8 on each ad libitum diet.
12. Supplementary Table 12: Unadjusted changes in self-reported dietary intake from baseline on each ad libitum diet (week 4 and week 8 average), and unadjusted and adjusted differences in changes in dietary intake from baseline between ad libitum diets for ITT and PP.
13. Supplementary Table 13: Self-reported energy intake at each timepoint by randomisation arm and period for ITT and PP.
14. Supplementary Table 14: Diet ratings overall
15. Supplementary Table 15: Diet ratings by randomisation arm
16. Supplementary Table 16: Unadjusted and adjusted changes in physical activity from baseline to week 8 on each diet for ITT and PP.

17. Supplementary Table 17: ITT: Changes in waist-to-height ratio and estimated energy imbalance from baseline to week 8 on each diet, and differences in changes from baseline to week 8 between diets.
18. Supplementary Table 18: ITT: Unadjusted changes in outcomes from baseline to week 4 on each diet, and unadjusted differences in changes in outcomes from baseline to week 4 between diets.
19. Supplementary Table 19: ITT: Repeated measures mixed-effects analysis of adjusted differences in changes in outcomes from baseline to week 4 and baseline to week 8 between diets.
20. Supplementary Table 20: PP: Unadjusted changes in outcomes from baseline to week 8 on each diet, and unadjusted differences in changes in outcomes from baseline to week 8 between diets.
21. Supplementary Table 21: PP: Changes in outcomes from baseline to week 8 on each diet, and differences in changes in outcomes from baseline to week 8 between diets.
22. Supplementary Table 22: PP: Unadjusted changes in outcomes from baseline to week 8 on each diet by randomisation arm, and unadjusted and adjusted differences in changes in outcomes from baseline to week 8 between first diets.
23. Supplementary Table 23: Sensitivity models for missing data, and model covariate selection results
24. Supplementary Table 24: Average nutrient composition of the provided MPF and UPF diets.
25. Supplementary Table 25: Menus for the provided MPF and UPF diets.
26. Supplementary Table 26: Pictures of meals and snacks of the provided MPF and UPF diets.
27. Supplementary Table 27: Descriptors of diet ratings.

Supplementary Table 1: Intention-to-treat and per-protocol: Baseline habitual dietary intakes overall and by randomisation arm

ITT N = 50	Overall		By randomisation arm				
			MPF/UPF (N = 23)		UPF/MPF (N = 27)		
	Mean	SE	Mean	SE	Mean	SE	p value
Total energy (kcal/day)	2178.8	93.0	2108.4	113.3	2238.8	143.6	0.490
MPF (% kcal/day)	22.5	0.9	21.0	1.4	23.8	1.1	0.107
PCI (% kcal/day)	2.4	0.4	1.7	0.5	3.0	0.5	0.068
PF (% kcal/day)	7.6	0.7	8.1	1.2	7.1	0.8	0.510
UPF (% kcal/day)	67.3	1.1	69.3	1.6	65.6	1.5	0.096
Fat (% kcal/day)	36.9	0.6	35.4	0.8	38.1	0.8	0.018
Saturated Fat (% kcal/day)	13.5	0.4	12.9	0.4	14.0	0.6	0.142
Carbohydrate (% kcal/day)	44.8	0.7	46.0	1.1	43.8	0.9	0.116
Total sugar (% kcal/day)	17.1	0.7	16.9	1.2	17.2	0.8	0.864
Total free sugar (% kcal/day)	10.1	0.6	10.2	0.9	10.1	0.7	0.947
Salt (g/day)	6.2	0.3	6.0	0.4	6.3	0.4	0.587
Protein (% kcal/day)	16.1	0.4	16.2	0.6	16.0	0.5	0.859
Fibre (g/day)	20.3	1.1	19.6	1.2	20.9	1.7	0.525
Alcohol (% kcal/day)	2.3	0.5	2.5	0.8	2.1	0.7	0.719
Red meat (g/day)	54.7	5.3	53.3	6.2	55.8	8.4	0.815
Oily fish (g/day)	8.6	2.1	7.3	3.4	9.7	2.7	0.564
Fruit and vegetables (portions/day)	3.4	0.2	3.4	0.4	3.3	0.3	0.832
**PP N = 43**			**(N = 20)**		**(N = 23)**		
Total energy (kcal/day)	2165.0	106.8	2129.1	129.9	2196.2	167.1	0.758
MPF (% kcal/day)	22.9	1.0	20.8	1.6	24.6	1.2	0.061
PCI (% kcal/day)	2.5	0.4	1.8	0.5	3.1	0.6	0.103
PF (% kcal/day)	8.0	0.8	8.8	1.3	7.2	0.8	0.324
UPF (% kcal/day)	66.4	1.2	68.6	1.8	64.4	1.5	0.077
Fat (% kcal/day)	36.8	0.6	35.6	0.9	37.9	0.8	0.055

Saturated Fat (% kcal/day)	13.4	0.4	12.9	0.5	13.9	0.7	0.238
Carbohydrate (% kcal/day)	44.4	0.7	45.0	1.1	43.9	1.0	0.424
Total sugar (% kcal/day)	16.9	0.8	16.2	1.2	17.6	0.9	0.366
Total free sugar (% kcal/day)	9.9	0.6	9.5	0.9	10.2	0.8	0.606
Salt (g/day)	6.1	0.3	6.1	0.4	6.0	0.5	0.915
Protein (% kcal/day)	16.3	0.4	16.7	0.6	16.0	0.6	0.439
Fibre (g/day)	20.6	1.2	19.9	1.4	21.2	2.0	0.623
Alcohol (% kcal/day)	2.5	0.6	2.8	0.9	2.2	0.8	0.644
Red meat (g/day)	52.6	5.7	55.8	6.9	49.9	9.0	0.614
Oily fish (g/day)	9.6	2.4	7.4	3.8	11.4	3.0	0.407
Fruit and vegetables (portions/day)	3.5	0.3	3.4	0.4	3.5	0.3	0.889

Abbreviations: ITT: intention-to-treat; MPF: minimally processed food; N: number; PCI: processed culinary ingredients, PF: processed food; PP: per protocol; SE: standard error; UPF: ultra-processed food.

Supplementary Table 2: Intention-to-treat: Baseline demographic and clinical characteristics overall and by randomisation arm.

ITT N = 50	Overall	By randomisation arm	
		MPF/UPF (23) (46.0%)	UPF/MPF (27) (54.0%)
Age at screening	42.6 (1.6)	42.3 (2.7)	42.9 (1.92)
Sex (Female)	45 (90.0%)	20 (87.0%)	25 (92.6%)
Ethnicity (White)	34 (68.0%)	19 (82.6%)	15 (55.6%)
Occupation			
Doctor	1 (2.0%)	1 (4.3%)	0 (0%)
Nurse	23 (46.0%)	8 (34.8%)	15 (55.6%)
AHP	9 (18.0%)	5 (21.7%)	4 (14.8%)
Management	4 (8.0%)	2 (8.7%)	2 (7.4%)
Administrative	8 (16.0%)	3 (13.0%)	5 (18.5%)
Other	5 (10.0%)	4 (17.4%)	1 (3.7%)
Night shift worker	8 (16.0%)	3 (13.0%)	5 (18.5%)
Education level			
None	0 (0.0%)	0 (0.0%)	0 (0.0%)
GCSE/O-level equivalent	3 (6.0%)	2 (8.7%)	1 (3.7%)
A level or equivalent	4 (8.0%)	1 (4.3%)	3 (11.1%)
Degree	22 (44.0%)	10 (43.5%)	12 (44.4%)
Postgraduate	21 (42.0%)	10 (43.5%)	11 (40.7%)
Other	0 (0.0%)	0 (0.0%)	0 (0.0%)
Marital status			
Single	18 (36.0%)	9 (39.1%)	9 (33.3%)
Married	16 (32.0%)	8 (34.8%)	8 (29.6%)
Living together	6 (12.0%)	2 (8.7%)	4 (14.8%)
Separated	2 (4.0%)	2 (8.7%)	0 (0%)
Divorced	4 (8.0%)	1 (4.3%)	3 (11.1%)
Widowed	4 (8.0%)	1 (4.3%)	3 (11.1%)
Civil partnership	0 (0.0%)	0 (0.0%)	0 (0.0%)
Family history of obesity	28 (56.0%)	13 (56.5%)	15 (55.6%)
Family history of diabetes	16 (32.0%)	6 (26.1%)	10 (37.0%)
Family history of cardiovascular disease	20 (40.0%)	9 (39.1%)	11 (40.7%)
Smoking			
Yes, current	5 (10.0%)	2 (8.7%)	3 (11.1%)
Yes past	16 (32.0%)	5 (21.7%)	11 (40.7%)
No, never	29 (58.0%)	16 (69.6%)	13 (48.1%)
AUDIT-C	2.5 (0.2)	2.5 (0.4)	2.4 (0.2)
Weekly units of alcohol	3.4 (0.7)	3.6 (1.3)	3.3 (0.7)
Weight (kg)	90.18 (1.78)	93.69 (2.75)	87.20 (2.22)
Height (m)	1.66 (0.01)	1.68 (0.01)	1.64 (0.01)
Estimated basal metabolic rate (kcal)	1695.88 (29.03)	1758.48 (45.68)	1642.56 (34.63)
BMI (kg/m^2)	32.74 (0.55)	33.30 (0.77)	32.26 (0.77)
BMI			

25-29.9 kg/m^2	16 (32.0%)	5 (21.7%)	11 (40.7%)
30-34.9 kg/m^2	20 (40.0%)	11 (47.8%)	9 (33.3%)
35-39.9 kg/m^2	14 (28.0%)	7 (30.4%)	7 (25.9%)
Waist circumference (cm)	97.34 (1.46)	100.41 (2.15)	94.72 (1.89)
Waist-to-height ratio	0.59 (0.01)	0.60 (0.01)	0.58 (0.01)
SBP (mmHg)	130.58 (1.97)	134.87 (3.06)	126.93 (2.39)
DBP (mmHg)	74.93 (1.31)	77.41 (1.91)	72.81 (1.74)
SBP >140mmHg or DBP >90mmHg	14 (28.0%)	7 (30.4%)	7 (25.9%)
HR (beats per minute)	5.54 (0.06)	5.47 (0.11)	5.61 (0.07)
HbA1c (%)	5.54 (0.06)	5.47 (0.11)	5.61 (0.07)
Fasting glucose (mmol/L)	4.90 [4.60;5.20]	5.00 [4.75;5.25]	4.80 [4.60;5.10]
Total cholesterol (mmol/L) (N = 49)	5.17 (0.13)	5.09 (0.19)	5.24 (0.16) (N = 26)
Total cholesterol >5mmol/L (N = 49)	29 (59.2%)	13 (56.5%)	16 (61.5%)
Participants self-reporting a condition	33 (66.0%)	18 (78.3%)	15 (55.6%)
Self-reporting medication use	32 (64.0%)	18 (78.3%)	14 (51.9%)
Self-reported antihypertensive use	7 (14.0%)	5 (21.7%)	2 (7.4%)
Self-reported hypercholesterolemia medication use	3 (6.0%)	2 (8.7%)	1 (3.7%)
Fat mass (kg)	34.51 (1.11)	36.06 (0.34)	33.20 (1.49)
Body fat percentage (%)	37.95 (0.66)	38.23 (0.19)	37.71 (0.97)
Visceral fat rating	9.32 (0.49)	9.96 (0.19)	8.78 (0.49)
Fat-free mass (kg)	55.67 (0.96)	57.63 (0.32)	54.00 (1.14)
Muscle mass (kg)	52.86 (0.91)	54.73 (1.45)	51.27 (1.08)
Bone mass (kg)	2.81 (0.05)	2.90 (0.07)	2.73 (0.06)
Total body water (kg)	37.59 (0.70)	38.63 (1.23)	36.70 (0.73)
Total body water percentage (%)	41.34 (0.49)	42.40 (0.82)	41.91 (0.72)
Bilirubin (umol/L)	8.0 [6.0;11.0]	9.0 [7.0;13.5]	8.0 [5.3;9.8]
Alkaline phosphatase (IU/L)	73.0 [60.5;84.0]	73.5 [61.8;84.5]	73.0 [59.0;83.0]
Alanine transaminase (IU/L)	21.0 [19.0;28.8]	25.5 [20.0;31.5]	20.0 [17.0;25.0]
Albumin (g/L) (N = 49)	44.9 (0.3)	45.7 (0.5)	44.2 (0.3) (N = 26)
Total cholesterol:HDL ratio	3.30 [2.70;4.20]	3.40 [2.85;4.20]	3.20 [2.55;4.05]
HDL-C (mmol/L)	1.50 [1.30;1.80]	1.50 [1.25;1.65]	1.60 [1.42;1.80]
LDL-C (mmol/L)	3.00 [2.40;3.60]	2.90 [2.45;3.50]	3.15 [2.42;3.60]
Non-HDL-C (mmol/L)	3.60 [2.90;4.30]	3.60 [3.00;4.20]	3.70 [2.90;4.30]
Triglycerides (mmol/L)	1.20 [0.90;1.60]	1.30 [0.85;1.60]	1.15 [0.90;1.40]
C-reactive protein (mg/L)	2.80 [1.17;5.00]	3.20 [1.85;4.80]	1.80 [0.60;5.00]
Average daily MVPA (minutes) (N= 45)	47.92 (3.91)	40.77 (5.52) (N = 21)	54.17 (5.30) (N = 24)

Data is presented as mean, count, or median where appropriate with corresponding percentage, standard error or interquartile range, respectively, in brackets. Abbreviations: AHP: allied health professional; AUDIT-C: Alcohol use disorders identification test - consumption; BMI, body mass index; DBP: diastolic blood pressure; HbA1c: haemoglobin A1c; HDL-C: high-density lipoprotein cholesterol; ITT: intention-to-treat; LDL-C: low-density lipoprotein cholesterol; MPF: minimally processed food; MVPA: moderate to vigorous physical activity; Non-HDL-C: non-high-density lipoprotein cholesterol; PCI: processed culinary ingredients; PF: processed food; SBP: systolic blood pressure; UPF: ultra-processed food.

Supplementary Table 3: Per-protocol: Baseline demographic and clinical characteristics overall and by randomisation arm.

PP N = 43	Overall	By randomisation arm	
		MPF/UPF (20) (46.5%)	UPF/MPF (23) (53.5%)
Age at screening	43.2 (1.7)	43.8 (2.7)	42.7 (2.2)
Sex (Female)	39 (90.7%)	17 (85.0%)	22 (95.7%)
Ethnicity (White)	29 (67.4%)	16 (80.0%)	13 (56.5%)
Occupation			
Doctor	1 (2.3%)	1 (5.0%)	0 (0.0%)
Nurse	20 (46.5%)	7 (35.0%)	13 (56.5%)
AHP	8 (18.6%)	4 (20.0%)	4 (17.4%)
Management	2 (4.7%)	1 (5.0%)	1 (4.3%)
Administrative	7 (16.3%)	3 (15.0%)	4 (17.4%)
Other	5 (11.6%)	4 (20.0%)	1 (4.3%)
Night shift worker	7 (16.3%)	3 (15.0%)	4 (17.4%)
Education level			
None	0 (0.0%)	0 (0.0%)	0 (0.0%)
GCSE/O-level equivalent	2 (4.7%)	2 (10.0%)	0 (0.0%)
A level or equivalent	3 (7.0%)	1 (5.0%)	2 (8.7%)
Degree	19 (44.2%)	8 (40.0%)	11 (47.8%)
Postgraduate	19 (44.2%)	9 (45.0%)	10 (43.5%)
Other	0 (0.0%)	0 (0.0%)	0 (0.0%)
Marital status			
Single	16 (37.2%)	8 (40.0%)	8 (34.8%)
Married	14 (32.6%)	7 (35.0%)	7 (30.4%)
Living together	3 (7.0%)	1 (5.0%)	2 (8.7%)
Separated	2 (4.7%)	2 (10.0%)	0 (0.0%)
Divorced	4 (9.3%)	1 (5.0%)	3 (13.0%)
Widowed	4 (9.3%)	1 (5.0%)	3 (13.0%)
Civil partnership	0 (0.0%)	0 (0.0%)	0 (0.0%)
Family history of obesity	25 (58.1%)	11 (55.0%)	14 (60.9%)
Family history of diabetes	15 (34.9%)	6 (30.0%)	9 (39.1%)
Family history of cardiovascular disease	16 (37.2%)	7 (35.0%)	9 (39.1%)
Smoking			
Yes, current	3 (7.0%)	2 (10.0%)	1 (4.3%)
Yes past	15 (34.9%)	5 (25.0%)	10 (43.5%)
No, never	25 (58.1%)	13 (65.0%)	12 (52.2%)
AUDIT-C	2.6 (0.2)	2.8 (0.4)	2.4 (0.3)
Weekly units of alcohol	3.7 (0.8)	4.0 (1.5)	3.4 (0.8)
Weight (kg)	89.8 (2.0)	94.4 (3.1)	85.9 (2.4)
Height (m)	1.66 (0.01)	1.68 (0.01)	1.65 (0.01)
Estimated basal metabolic rate (kcal)	1693.72 (33.16)	1773.20 (51.81)	1624.61 (37.98)
BMI (kg/m^2)	32.40 (0.58)	33.36 (0.87)	31.56 (3.59)
BMI			

25-29.9 kg/m^2	15 (34.88%)	5 (25.00%)	10 (43.48%)
30-34.9 kg/m^2	17 (39.53%)	8 (40.00%)	9 (39.13%)
35-39.9 kg/m^2	11 (25.58%)	7 (35.00%)	4 (17.39%)
Waist circumference (cm)	96.99 (1.61)	101.05 (2.39)	93.46 (1.95)
Waist-to-height ratio	0.58 (0.01)	0.60 (0.01)	0.57 (0.01)
SBP (mmHg)	130.3 (2.3)	135.6 (3.4)	125.8 (2.7)
DBP (mmHg)	74.0 (1.3)	77.7 (2.0)	70.8 (1.5)
SBP >140mmHg or DBP >90mmHg	12 (27.91%)	7 (35.00%)	5 (21.74%)
HbA1c (%)	5.53 (0.07)	5.49 (0.12)	5.56 (0.08)
Fasting glucose (mmol/L)	4.80 (4.60-5.20)	5.00 (4.68-5.30)	4.80 (4.53-5.18)
Total cholesterol (mmol/L)	5.18 (0.14)	5.25 (0.19)	5.09 (0.20)
Total cholesterol >5mmol/L	25 (58.14%)	11 (55.00%)	14 (60.87%)
Participants self-reporting a condition	27 (62.8%)	16 (80.0%)	11 (47.8%)
Self-reporting medication use	26 (60.5%)	15 (75.0%)	11 (47.8%)
Self-reported antihypertensive use	6 (14.0%)	5 (25.0%)	1 (4.3%)
Self-reported hypercholesterolemia medication use	2 (4.7%)	2 (10.0%)	0 (0.0%)
Average daily MVPA (minutes) (N= 42)	48.04 (4.05)	42.25 (5.59)	53.31 (5.71) (N = 22)

Data is presented as mean, count, or median where appropriate with corresponding percentage, standard error or interquartile range, respectively, in brackets. Abbreviations: AHP: allied health professional; AUDIT-C: Alcohol use disorders identification test - consumption; BMI, body mass index; DBP: diastolic blood pressure; HbA1c: haemoglobin A1c; MPF: minimally processed food; MVPA: moderate to vigorous physical activity; PCI: processed culinary ingredients; PF: processed food; PP: per-protocol; SBP: systolic blood pressure; UPF: ultra-processed food.

Supplementary Table 4: Baseline demographic, clinical and dietary characteristics by dropouts versus non-dropouts.

N = 55	No dropout (N = 43)	Dropout (N = 12)	p value
Age at screening	43.2 (1.7)	43.4 (3.8)	0.947
Sex (Female)	39 (90.70%)	11 (91.67%)	1.000
Ethnicity (White)	29 (67.4%)	7 (58.3%)	0.733
Occupation			0.161
Doctor	1 (2.3%)	0 (0%)	
Nurse	20 (46.5%)	4 (33.3%)	
AHP	8 (18.6%)	1 (8.3%)	
Management	2 (4.7%)	3 (25.0%)	
Administrative	7 (16.3%)	4 (33.3%)	
Other	5 (11.6%)	0 (0%)	
Night shift worker	7 (16.3%)	2 (16.7%)	1.000
Education level			0.172
None	0 (0.0%)	0 (0.0%)	
GCSE/O-level equivalent	2 (4.7%)	2 (16.7%)	
A level or equivalent	3 (7.0%)	1 (8.3%)	
Degree	19 (44.2%)	7 (58.3%)	
Postgraduate	19 (44.2%)	2 (16.7%)	
Other	0 (0.0%)	0 (0.0%)	
Marital status			0.393
Single	16 (37.2%)	5 (41.7%)	
Married	14 (32.6%)	3 (25.0%)	
Living together	3 (7.0%)	3 (25.0%)	
Separated	2 (4.7%)	1 (8.3%)	
Divorced	4 (9.3%)	0 (0%)	
Widowed	4 (9.3%)	0 (0%)	
Civil partnership	0 (0.0%)	0 (0.0%)	
Family history of obesity	25 (58.1%)	5 (41.7%)	0.493
Family history of diabetes	15 (34.9%)	2 (16.7%)	0.304
Family history of cardiovascular disease	16 (37.2%)	5 (41.7%)	1.000
Smoking			0.317

Yes, current	3 (6.98%)	2 (16.67%)	
Yes past	15 (34.88%)	2 (16.67%)	
No, never	25 (58.14%)	8 (66.67%)	
AUDIT-C	2.58 (0.22)	2.42 (0.45)	0.736
Weekly units of alcohol	3.71 (0.78)	1.83 (0.59)	0.061
Weight (kg)	89.81 (2.01)	87.98 (3.56)	0.668
BMI (kg/m^2)	32.40 (0.58)	33.87 (1.26)	0.258
Total energy (kcal/day)	2164.99 (106.77)	1970.43 (134.46)	0.370
UPF (% kcal/day)	66.38 (1.18)	71.06 (2.42)	0.074
Average daily MVPA (minutes) (N = 48)	48.04 (4.05) (N = 42)	41.56 (10.28) (N = 6)	0.668

Data is presented as mean, count, or median where appropriate with corresponding percentage, standard error or interquartile range, respectively, in brackets. Abbreviations: AHP: allied health professional; AUDIT-C: Alcohol use disorders identification test - consumption; BMI, body mass index; MVPA: moderate to vigorous physical activity; UPF: ultra-processed food.

Supplementary Table 5: Intention-to-treat: Unadjusted changes in outcomes from baseline to week 8 on each diet, and differences in changes in outcomes from baseline to week 8 between diets.

ITT N = 50	MPF diet				UPF diet				MPF diet - UPF diet			
	N	Mean	SE	p-value	N	Mean	SE	p-value	N	Mean	SE	p-value
Percentage weight change (%)	48	-2.73	0.5	<0.001	48	-1.73	0.3	<0.001	46	-0.72	0.5	0.150
Weight (kg)	48	-2.54	0.4	<0.001	48	-1.59	0.3	<0.001	46	-0.70	0.5	0.132
BMI (kg/m^2)	48	-0.91	0.2	<0.001	48	-0.58	0.1	<0.001	46	-0.24	0.2	0.146
Waist circumference (cm)	45	-2.27	0.8	0.005	46	-1.18	0.8	0.135	43	-0.67	1.1	0.559
Waist-to-height ratio	45	-0.01	0.0	0.005	46	-0.01	0.0	0.125	43	0.00	0.0	0.563
Fat mass (kg)	47	-2.08	0.4	<0.001	48	-1.05	0.3	<0.001	45	-0.73	0.4	0.047
Body fat percentage (%)	47	-1.26	0.3	<0.001	48	-0.49	0.2	0.008	45	-0.56	0.3	0.054
Visceral fat rating	47	-0.70	0.1	<0.001	48	-0.31	0.1	0.001	45	-0.36	0.2	0.037
Fat-free mass (kg)	47	-0.54	0.2	0.029	48	-0.54	0.2	0.001	45	-0.02	0.3	0.946
Muscle mass (kg)	47	-0.52	0.2	0.028	48	-0.51	0.1	0.001	45	-0.03	0.3	0.925
Bone mass (kg)	47	-0.02	0.0	0.086	48	-0.03	0.0	0.001	45	0.01	0.0	0.673
Total body water (kg)	47	-0.88	0.1	<0.001	48	-0.39	0.1	0.012	45	-0.48	0.2	0.005
Total body water percentage (%)	47	0.26	0.1	0.083	48	0.33	0.1	0.017	45	-0.18	0.2	0.279
SBP (mmHg)	47	-4.73	1.7	0.008	47	-2.03	2.0	0.305	45	-2.68	2.6	0.302
DBP (mmHg)	47	-3.34	1.2	0.008	47	-1.64	0.8	0.060	45	-2.14	1.4	0.140
HR (beats per minute)	46	-3.39	1.6	0.034	47	-4.28	1.7	0.016	44	0.98	2.3	0.674
Bilirubin (umol/L)	42	0.07	0.5	0.876	44	0.61	0.5	0.213	38	-0.50	0.5	0.350
Alkaline phosphatase (IU/L)	36	0.97	0.9	0.297	40	0.05	1.7	0.977	29	0.86	1.9	0.646
Alanine transaminase (IU/L)	35	2.86	2.6	0.280	39	0.90	1.7	0.604	27	4.52	3.0	0.142
Albumin (g/L)	44	0.43	0.4	0.227	44	0.45	0.3	0.189	40	-0.15	0.5	0.779
HbA1C (%)	46	-0.08	0.0	0.026	47	-0.03	0.0	0.190	45	-0.05	0.0	0.246
Fasting glucose (mmol/L)	46	-0.09	0.1	0.148	46	-0.14	0.1	0.007	44	0.04	0.1	0.590
Total cholesterol (mmol/L)	41	-0.28	0.1	0.006	42	-0.41	0.1	<0.001	35	0.07	0.1	0.619

Total cholesterol:HDL ratio	41	0.05	0.1	0.501	42	-0.05	0.1	0.453	35	0.10	0.1	0.461
HDL-C (mmol/L)	43	-0.14	0.0	0.001	44	-0.12	0.0	<0.001	39	-0.04	0.1	0.455
LDL-C (mmol/L)	41	-0.09	0.1	0.234	42	-0.34	0.1	<0.001	35	0.22	0.1	0.082
Non-HDL-C (mmol/L)	41	-0.16	0.1	0.088	42	-0.30	0.1	<0.001	35	0.10	0.1	0.464
Triglycerides (mmol/L)	41	-0.15	0.1	0.037	42	0.10	0.0	0.019	35	-0.25	0.1	0.012
C-reactive protein (mg/L)	44	-0.75	0.3	0.037	43	-0.33	0.5	0.540	39	-0.28	0.7	0.687
PFS food available	39	-0.55	0.2	0.001	36	-0.37	0.2	0.021	33	-0.10	0.2	0.651
PFS food present	39	-0.67	0.2	0.000	36	-0.51	0.2	0.005	33	0.02	0.2	0.922
PFS food tasted	39	-0.38	0.1	0.014	36	-0.27	0.1	0.017	33	-0.12	0.2	0.489
PFS total	39	-0.53	0.1	0.001	36	-0.38	0.1	0.004	33	-0.06	0.2	0.714
CoEQ craving control	39	21.79	4.1	<0.001	36	13.09	4.0	0.002	33	6.30	5.5	0.264
CoEQ craving for sweet	39	-16.61	2.6	<0.001	36	-10.67	2.5	0.000	33	-4.19	3.9	0.291
CoEQ craving for savoury	39	-13.71	3.1	<0.001	35	-5.14	2.7	0.062	33	-6.76	4.6	0.153
CoEQ positive mood	39	0.76	2.5	0.768	36	4.06	2.6	0.131	33	-3.12	3.1	0.325
CoEQ control over craved nominated food	39	-26.41	5.3	<0.001	36	-16.56	6.0	0.009	33	-9.00	7.1	0.212

Unadjusted change from baseline and between diets assessed using paired t-test. Abbreviations: BMI: body mass index; CoEQ: Control of Eating Questionnaire; CRP: c-reactive protein; DBP: diastolic blood pressure; HbA1c: glycated haemoglobin; HDL-C: high-density lipoprotein cholesterol; HR: heart rate; ITT: intention-to-treat; LDL-C: low-density lipoprotein cholesterol; MPF: minimally processed food; Non-HDL-C: Non-high-density lipoprotein cholesterol; PFS: Power of Food Scale; SBP: systolic blood pressure; SE: standard error; UPF: ultra-processed food.

Supplementary Table 6: Intention-to-treat: Unadjusted changes in outcomes from baseline to week 8 on each diet by randomisation arm, and unadjusted and adjusted differences in changes in outcomes from baseline to week 8 between first diets.

ITT N = 50	MPF diet								UPF diet								MPF diet first - UPF diet first							
	Arm MPF/UPF - first				Arm UPF/MPF - second				Arm MPF/UPF - second				Arm UPF/MPF - first				Unadjusted					Mixed-effects model		
	N	Mean	SE	p	N	Mean	SE	p	N	Mean	SE	p	N	Mean	SE	p	N	Mean	Lower 95%CI	Upper 95%CI	p	Mean	SE	p
Percentage weight change (%)	23	-4.09	0.7	<0.001	25	-1.49	0.5	0.010	21	-1.24	0.5	0.020	27	-2.12	0.5	<0.001	50	-1.97	-3.54	-0.40	0.015	-1.86	0.72	0.012
Weight change (kg)	23	-3.86	0.6	<0.001	25	-1.32	0.5	0.008	21	-1.15	0.4	0.016	27	-1.93	0.4	<0.001	50	-1.93	-3.43	-0.44	0.012	-1.82	0.66	0.007
BMI change (kg/m2)	23	-1.38	0.2	<0.001	25	-0.48	0.2	0.008	21	-0.40	0.2	0.018	27	-0.71	0.2	<0.001	50	-0.67	-1.21	-0.13	0.015	-0.63	0.24	0.010
Waist circumference (cm)	20	-4.33	1.2	0.002	25	-0.62	0.9	0.500	19	1.00	1.2	0.424	27	-2.72	0.9	0.006	47	-1.60	-4.58	1.38	0.284	-1.43	1.46	0.331
Waist-to-height ratio	23	-0.03	0.0	0.002	25	0.00	0.0	0.494	19	0.01	0.0	0.444	27	-0.02	0.0	0.006	47	-0.01	-0.03	0.01	0.289	-0.01	0.01	0.341
Fat mass (kg)	23	-3.09	0.6	<0.001	24	-1.12	0.4	0.006	21	-0.60	0.3	0.083	27	-1.40	0.4	0.001	50	-1.69	-2.99	-0.39	0.012	-1.62	0.57	0.006
Body fat percentage (%)	23	-1.85	0.4	0.000	24	-0.70	0.4	0.061	21	-0.19	0.2	0.443	27	-0.73	0.2	0.007	50	-1.13	-2.11	-0.15	0.026	-1.10	0.45	0.017
Visceral fat rating	23	-1.09	0.2	<0.001	24	-0.33	0.2	0.043	21	-0.19	0.1	0.104	27	-0.41	0.1	0.003	50	-0.68	-1.12	-0.24	0.003	-0.66	0.20	0.002
Fat-free mass (kg)	23	-0.77	0.3	0.007	24	-0.33	0.4	0.425	21	-0.55	0.2	0.017	27	-0.53	0.2	0.024	50	-0.24	-0.92	0.44	0.481	-0.20	0.40	0.298
Muscle mass (kg)	23	-0.73	0.2	0.007	24	-0.31	0.4	0.421	21	-0.51	0.2	0.019	27	-0.50	0.2	0.025	50	-0.23	-0.88	0.42	0.477	-0.19	0.37	0.601
Bone mass (kg)	23	-0.03	0.0	0.043	24	-0.01	0.0	0.560	21	-0.04	0.0	0.017	27	-0.04	0.0	0.032	50	0.00	-0.05	0.03	0.650	-0.01	0.02	0.775
Total body water (kg)	23	-1.13	0.2	<0.001	24	-0.64	0.1	<0.001	21	-0.24	0.2	0.292	27	-0.50	0.2	0.019	50	-0.63	-1.18	-0.09	0.023	-0.60	0.24	0.016
Total body water percentage (%)	23	0.55	0.2	0.019	24	-0.01	0.2	0.966	21	0.34	0.2	0.138	27	0.32	0.2	0.064	50	0.23	-0.31	0.77	0.396	0.22	0.28	0.423
SBP (mmHg)	22	-9.70	2.0	<0.001	25	-0.36	2.4	0.881	20	-1.85	2.9	0.533	27	-2.17	2.7	0.428	49	-7.54	-14.53	-0.54	0.035	-7.64	3.54	0.034
DBP (mmHg)	22	-5.30	1.5	0.002	25	-1.62	1.8	0.383	20	-3.10	1.3	0.025	27	-0.56	1.1	0.620	49	-4.74	-8.38	-1.10	0.012	-4.75	2.04	0.022
HR (beats per minute)	22	-4.18	1.9	0.041	24	-2.67	2.4	0.282	20	-3.75	2.3	0.117	27	-4.67	2.5	0.070	49	0.48	-6.04	7.01	0.882	0.47	3.25	0.887
Bilirubin (umol/L)	21	0.05	0.6	0.940	21	0.10	0.7	0.889	20	1.65	0.8	0.043	24	-0.25	0.6	0.671	45	0.30	-1.42	2.02	0.729	0.34	0.92	0.718
Alkaline phosphatase (IU/L)	19	0.84	1.3	0.519	17	1.12	1.4	0.423	19	0.79	2.0	0.693	21	-0.62	2.8	0.830	40	1.46	-4.92	7.84	0.642	1.30	2.86	0.650
Alanine transaminase (IU/L)	18	4.61	4.3	0.297	17	1.00	2.9	0.737	19	1.21	3.0	0.690	20	0.60	1.9	0.751	38	4.01	-5.65	13.67	0.400	4.18	4.27	0.332
Albumin (g/L)	21	0.76	0.4	0.057	23	0.13	0.6	0.824	20	0.55	0.5	0.308	24	0.38	0.5	0.417	45	0.39	-0.80	1.58	0.516	0.27	0.67	0.684
HbA1C (%)	21	-0.18	0.1	0.003	25	0.01	0.0	0.822	20	-0.02	0.0	0.577	27	-0.04	0.0	0.242	48	-0.14	-0.27	-0.01	0.038	-0.14	0.06	0.017
Fasting glucose (mmol/L)	21	-0.11	0.1	0.251	25	-0.06	0.1	0.395	20	-0.17	0.1	0.010	26	-0.12	0.1	0.129	47	0.01	-0.24	0.26	0.943	0.00	0.11	0.984
Total cholesterol (mmol/L)	20	-0.33	0.1	0.009	21	-0.23	0.2	0.152	20	-0.40	0.1	0.002	22	-0.42	0.1	0.000	42	0.10	-0.20	0.39	0.505	0.09	0.17	0.593

Total cholesterol:HDL ratio	20	0.10	0.1	0.369	21	0.00	0.1	0.965	20	-0.05	0.1	0.612	22	-0.05	0.1	0.591	42	0.15	-0.14	0.45	0.301	0.13	0.14	0.344
HDL-C (mmol/L)	20	-0.20	0.1	0.009	23	-0.09	0.0	0.018	20	-0.10	0.0	0.007	24	-0.13	0.0	0.004	44	-0.07	-0.20	0.13	0.395	-0.06	0.06	0.340
LDL-C (mmol/L)	20	-0.13	0.1	0.212	21	-0.06	0.1	0.624	20	-0.34	0.1	0.004	22	-0.35	0.1	<0.001	42	0.23	-0.02	0.47	0.071	0.22	0.14	0.124
Non-HDL-C (mmol/L)	20	-0.18	0.1	0.103	21	-0.14	0.2	0.354	20	-0.31	0.1	0.006	22	-0.30	0.1	0.002	42	0.13	-0.14	0.39	0.354	0.11	0.16	0.483
Triglycerides (mmol/L)	20	-0.11	0.1	0.248	21	-0.19	0.1	0.087	20	0.06	0.1	0.304	22	0.14	0.1	0.030	42	-0.25	-0.46	-0.03	0.027	-0.25	0.12	0.031
C-reactive protein (mg/L)	21	-0.75	0.3	0.028	23	-0.75	0.6	0.230	20	-0.51	0.9	0.580	23	-0.17	0.6	0.787	44	-0.58	-1.99	0.84	0.412	-0.59	0.90	0.518
PFS food available	17	-1.06	0.2	<0.001	22	-0.16	0.2	0.349	12	-0.10	0.3	0.745	24	-0.51	0.2	0.008	41	-0.55	-1.14	0.034	0.064	-0.46	0.29	0.120
PFS food present	17	-1.27	0.3	<0.001	22	-0.20	0.2	0.215	12	0.00	0.3	1.000	24	-0.76	0.2	0.001	41	-0.50	-1.18	0.175	0.141	-0.49	0.32	0.131
PFS food tasted	17	-0.67	0.2	0.015	22	-0.16	0.2	0.353	12	-0.13	0.3	0.627	24	-0.34	0.1	0.002	41	-0.33	-0.88	0.222	0.228	-0.33	0.26	0.220
PFS total	17	-1.00	0.2	<0.001	22	-0.18	0.2	0.274	12	-0.08	0.3	0.765	24	-0.54	0.1	0.001	41	-0.46	-0.96	0.038	0.069	-0.43	0.26	0.101
CoEQ craving control	17	36.93	5.5	<0.001	22	10.09	4.6	0.040	12	6.92	4.6	0.165	24	16.18	5.4	0.007	41	20.75	4.671	36.82	0.013	20.97	7.59	0.008
CoEQ craving for sweet	17	-21.35	3.5	<0.001	22	-12.95	3.7	0.002	12	-6.20	3.1	0.073	24	-12.91	3.3	0.001	41	-8.44	-18.37	1.476	0.093	-4.84	4.79	0.316
CoEQ craving for savoury	17	-20.37	4.6	<0.001	22	-8.57	3.9	0.040	12	0.15	4.2	0.973	23	-7.90	3.3	0.027	40	-12.47	-23.62	-1.31	0.029	-11.93	5.73	0.041
CoEQ positive mood	17	1.59	4.6	0.734	22	0.11	2.9	0.969	12	6.79	4.6	0.165	24	2.70	3.2	0.414	41	-1.11	-12.14	9.926	0.840	-1.95	5.26	0.713
CoEQ control over craved nominated food	17	-37.94	7.6	<0.001	22	-17.50	7.0	0.020	12	-7.92	7.3	0.299	24	-20.88	8.2	0.018	41	-17.06	-40.67	6.534	0.152	-17.78	11.30	0.120

Unadjusted change from baseline assessed using paired t-test and between diets using two-sample t-test. Estimated marginal means and 95% confidence intervals computed from mixed-effects models adjusted for randomisation arm and night-shift status, with an interaction term for diet and randomisation arm, and a random effect for participant. Abbreviations: 95%CI: 95% confidence interval; BMI: body mass index; CoEQ: Control of Eating Questionnaire; CRP: c-reactive protein; DBP: diastolic blood pressure; HbA1c: glycated haemoglobin; HDL-C: high-density lipoprotein cholesterol; HR: heart rate; ITT: intention-to-treat; LDL-C: low-density lipoprotein cholesterol; MPF: minimally processed food; Non-HDL-C: Non-high-density lipoprotein cholesterol; PFS: Power of Food Scale; SBP: systolic blood pressure; SE: standard error; UPF: ultra-processed food.

Supplementary Table 7: Intention-to-treat: Unadjusted and adjusted changes in fasted and fed subjective appetite visual analogue scales from baseline to week 8 on each diet.

ITT	Time (minutes)	How hungry do you feel right now?			How sick do you feel right now?			How much do you think you could eat right now?			How full do you feel right now?			How pleasant would it be to eat right now?		
Unadjusted		Mean	SE	p-value	Mean	SE	p-value	Mean	SE	p-value	Mean	SE	p-value	Mean	SE	p-value
MPF diet (N = 46)	0 (fasted)	0.1	0.4	0.780	0.2	0.3	0.475	-0.1	0.3	0.665	-0.4	0.4	0.310	0.1	0.3	0.662
MPF diet (N = 44)	15 (fed)	0.0	0.4	0.952	0.2	0.3	0.572	-0.3	0.4	0.433	-0.7	0.4	0.054	-0.1	0.4	0.759
MPF diet (N = 46)	30 (fed)	-0.2	0.4	0.638	0.4	0.2	0.039	-0.2	0.3	0.450	-0.4	0.4	0.387	-0.3	0.4	0.481
UPF diet (N = 47)	0 (fasted)	0.2	0.3	0.594	0.2	0.3	0.424	0.0	0.3	0.894	-0.3	0.3	0.337	-0.1	0.3	0.809
UPF diet (N = 46)	15 (fed)	0.6	0.3	0.078	-0.4	0.2	0.074	0.1	0.3	0.682	0.1	0.3	0.821	0.6	0.4	0.096
UPF diet (N = 45)	30 (fed)	0.2	0.4	0.542	0.1	0.2	0.582	0.1	0.3	0.763	0.2	0.4	0.582	0.3	0.3	0.393
Adjusted		Mean	Lower 95%CI	Upper 95%CI	Mean	Lower 95%CI	Upper 95%CI	Mean	Lower 95%CI	Upper 95%CI	Mean	Lower 95%CI	Upper 95%CI	Mean	Lower 95%CI	Upper 95%CI
MPF diet (N = 46)	0 (fasted)	-0.1	-0.9	0.7	0.4	-0.1	1.0	-0.2	-1.0	0.5	-0.2	-1.0	0.6	0.0	-0.7	0.8
MPF diet (N = 44)	15 (fed)	-0.2	-1.0	0.7	0.3	-0.2	0.9	-0.4	-1.1	0.4	-0.5	-1.3	0.3	-0.2	-1.0	0.6
MPF diet (N = 46)	30 (fed)	-0.4	-1.2	0.4	0.6	0.0	1.1	-0.3	-1.1	0.4	-0.1	-1.0	0.7	-0.4	-1.2	0.4
UPF diet (N = 47)	0 (fasted)	0.0	-0.8	0.8	0.4	-0.2	0.9	0.0	-0.7	0.7	-0.1	-0.9	0.7	-0.1	-0.8	0.7
UPF diet (N = 46)	15 (fed)	0.4	-0.4	1.2	-0.2	-0.8	0.3	0.1	-0.6	0.8	0.3	-0.5	1.1	0.6	-0.2	1.4
UPF diet (N = 45)	30 (fed)	0.0	-0.8	0.8	0.2	-0.3	0.8	0.0	-0.7	0.7	0.5	-0.3	1.3	0.3	-0.5	1.0

Unadjusted change from baseline on each diet assessed using paired t-test. Estimated marginal means and 95% confidence intervals computed from mixed-effects models adjusted for randomisation arm and night-shift status, with an interaction term for diet and randomisation arm, and a random effect for participant. Abbreviations: 95%CI: 95% confidence interval; ITT: intention-to-treat; MPF: minimally processed food; SE: standard error; UPF: ultra-processed food.

Supplementary Table 8: Intention-to-treat and per-protocol: Adjusted differences in fasted and fed subjective appetite visual analogue scales from baseline to week 8 for ITT, PP and for first diets only.

	Change from baseline to week 8, MPF diet - UPF diet

	0 minutes (fasted)			15 minutes (fed)			30 minutes (fed)			Overall (0, 15 and 30 minutes)
	Mean	SE	p-value	Mean	SE	p-value	Mean	SE	p-value	p-value
**ITT = 50**										
How hungry do you feel right now?	-0.1	0.5	0.854	-0.5	0.5	0.261	-0.4	0.5	0.408	0.220
How sick do you feel right now?	0.0	0.3	0.936	0.6	0.3	0.109	0.3	0.3	0.365	0.136
How much do you think you could eat right now?	-0.2	0.4	0.602	-0.5	0.4	0.269	-0.3	0.4	0.408	0.160
How full do you feel right now?	-0.1	0.5	0.819	-0.8	0.5	0.082	-0.6	0.5	0.180	0.058
How pleasant would it be to eat right now?	0.1	0.5	0.787	-0.9	0.5	0.065	-0.6	0.5	0.165	0.087
**PP = 43**										
How hungry do you feel right now?	0.1	0.5	0.907	-0.7	0.5	0.181	-0.3	0.5	0.598	0.309
How sick do you feel right now?	0.1	0.4	0.848	0.7	0.4	0.070	0.4	0.4	0.293	0.077
How much do you think you could eat right now?	-0.3	0.4	0.528	-0.6	0.4	0.152	-0.1	0.4	0.838	0.190
How full do you feel right now?	-0.2	0.5	0.754	-0.7	0.5	0.151	-0.6	0.5	0.217	0.085
How pleasant would it be to eat right now?	0.2	0.5	0.742	-0.8	0.5	0.091	-0.6	0.5	0.211	0.128
**First-period diets only**										
How hungry do you feel right now?	0.2	0.8	0.835	-0.5	0.8	0.511	-0.4	0.8	0.595	0.674
How sick do you feel right now?	0.0	0.5	0.961	0.7	0.5	0.152	0.4	0.5	0.396	0.271
How much do you think you could eat right now?	-0.3	0.7	0.696	-1.0	0.7	0.188	-0.5	0.7	0.479	0.325
How full do you feel right now?	-0.5	0.7	0.529	-0.7	0.7	0.329	0.1	0.8	0.909	0.533
How pleasant would it be to eat right now?	0.2	0.8	0.768	-1.1	0.8	0.164	-1.0	0.8	0.196	0.314

A negative value indicates either a smaller reduction or a greater increase on the UPF than MPF diet. A positive value indicates either a greater reduction or a smaller increase on the UPF than MPF diet. Estimated marginal means and 95% confidence intervals computed from mixed-effects models adjusted for randomisation arm and night-shift status, with an interaction term for diet and randomisation arm, and a random effect for participant. Abbreviations: 95%CI: 95% confidence interval; ITT: intention-to-treat; MPF: minimally processed food; PP: per-protocol; SE: standard error; UPF: ultra-processed food.

Supplementary Table 9: ITT: Unadjusted self-reported dietary intakes at baseline, week 4 and week 8 on each ad libitum diet.

ITT N = 50	MPF diet			UPF diet		
	Baseline (N = 50)	Week 4 (N = 34)	Week 8 (N = 33)	Baseline (N = 48)	Week 4 (N = 43)	Week 8 (N = 36)

	Mean	SE	Mean	SE	Mean	SE	Mean	SE	Mean	SE	Mean	SE
Total energy (kcal/day)	1938.0	85.0	1334.8	80.3	1463.1	110.9	2009.2	100.9	1763.7	66.1	1769.7	96.7
Fat (% kcal/day)	36.4	0.8	30.9	1.1	33.3	1.0	37.6	0.8	29.4	0.9	31.7	1.1
Saturated Fat (% kcal/day)	12.7	0.4	9.3	0.5	10.0	0.7	13.2	0.5	8.5	0.4	10.0	0.6
Carbohydrate (% kcal/day)	45.9	0.9	47.4	1.3	43.1	1.3	43.2	0.8	51.8	1.2	49.8	1.3
Total sugar (% kcal/day)	16.9	0.9	18.6	1.0	15.9	0.9	16.9	0.7	18.2	0.9	17.1	0.8
Total free sugar (% kcal/day)	9.7	0.7	3.2	0.5	3.2	0.4	9.2	0.6	10.4	0.7	9.2	0.5
Salt (g/day)	5.3	0.3	2.5	0.2	2.6	0.3	5.8	0.3	4.3	0.3	4.5	0.3
Protein (% kcal/day)	16.2	0.4	20.6	0.6	21.0	0.7	17.0	0.4	17.6	0.5	17.3	0.5
Fibre (g/day)	19.1	0.9	19.4	1.4	20.6	1.7	18.8	1.2	25.3	1.1	23.8	1.8
Alcohol (% kcal/day)	1.6	0.4	1.1	0.4	2.6	1.1	2.2	0.6	1.1	0.5	1.0	0.5
Red meat (g/day)	49.6	6.2	49.0	5.9	39.0	8.5	59.0	6.8	30.1	5.4	33.6	8.0
Oily fish (g/day)	10.5	3.0	20.3	7.1	23.4	7.8	6.4	1.9	6.7	2.3	6.6	3.0
Fruit and vegetables (portions/day)	3.4	0.3	5.1	0.4	5.0	0.5	3.7	0.3	3.1	0.2	3.2	0.4

Abbreviations: ITT: intention-to-treat; MPF: minimally processed food; PP: per-protocol; SE: standard error; UPF: ultra-processed food.

Supplementary Table 10: Unadjusted self-reported dietary intakes on each ad libitum diet (week 4 and week 8 average) and unadjusted and adjusted differences in dietary intake between each ad libitum diet for ITT and PP.

ITT N = 50	MPF diet	UPF diet	MPF - UPF diet (N = 36) Unadjusted	MPF - UPF diet (N = 48) Adjusted
	Week 4 and 8 (N = 39)	Week 4 and 8 (N = 45)		

	Mean	SE	Mean	SE	Mean	SE	p-value	Mean	SE	p-value
Total energy (kcal/day)	1420.4	92.1	1744.7	64.0	-347.1	118.1	0.006	-327.3	110.2	0.005
Fat (% kcal/day)	32.0	0.6	30.5	0.8	2.0	1.2	0.104	1.5	1.0	0.148
Saturated Fat (% kcal/day)	9.6	0.4	9.1	0.3	0.4	0.5	0.459	0.4	0.5	0.380
Carbohydrate (% kcal/day)	45.4	0.8	50.8	1.0	-6.2	1.5	<0.001	-5.4	1.3	<0.001
Total sugar (% kcal/day)	17.3	0.7	17.6	0.8	-0.6	1.0	0.577	-0.2	1.0	0.851
Total free sugar (% kcal/day)	3.1	0.3	9.9	0.6	-6.7	0.8	<0.001	-6.7	0.7	<0.001
Salt (g/day)	2.7	0.2	4.3	0.2	-1.6	0.3	<0.001	-1.6	0.3	<0.001
Protein (% kcal/day)	21.2	0.6	17.5	0.4	3.7	0.8	<0.001	3.8	0.7	<0.001
Fibre (g/day)	20.2	1.5	24.3	1.2	-4.8	1.9	0.017	-4.1	1.8	0.031
Alcohol (% kcal/day)	1.4	0.4	1.2	0.5	0.6	0.4	0.187	0.5	0.4	0.314
Red meat (g/day)	48.3	7.4	31.0	4.4	19.3	7.8	0.019	17.4	7.3	0.023
Oily fish (g/day)	21.2	6.1	6.4	2.2	15.8	7.4	0.039	14.6	6.2	0.024
Fruit and vegetables (portions/day)	5.1	0.4	3.1	0.2	1.9	0.4	<0.001	2.0	0.3	<0.001
**PP N = 43**	**MPF diet**		**UPF diet**		**MPF - UPF diet (N = 34) Unadjusted**			**MPF - UPF diet (N = 42) Adjusted**		
	**Week 4 and 8 (N = 35)**		**Week 4 and 8 (N = 41)**							
	**Mean**	**SE**	**Mean**	**SE**	**Mean**	**SE**	**p-value**	**Mean**	**SE**	**p-value**
Total energy (kcal/day)	1432.4	97.4	1739.9	66.1	-340.83	125.0	0.010	-311.3	117.2	0.012
Fat (% kcal/day)	31.7	0.6	30.1	0.7	1.5	1.2	0.224	1.6	1.0	0.105
Saturated Fat (% kcal/day)	9.7	0.4	9.1	0.4	0.3	0.5	0.527	0.5	0.5	0.306
Carbohydrate (% kcal/day)	45.5	0.8	51.3	1.0	-5.8	1.5	0.001	-5.7	1.3	<0.001
Total sugar (% kcal/day)	17.6	0.7	17.6	0.8	-0.1	1.0	0.929	0.1	1.0	0.900
Total free sugar (% kcal/day)	2.9	0.3	9.8	0.6	-6.8	0.8	<0.001	-6.9	0.7	<0.001
Salt (g/day)	2.6	0.2	4.3	0.2	-1.7	0.3	<0.001	-1.7	0.3	<0.001
Protein (% kcal/day)	21.2	0.7	17.4	0.4	3.7	0.8	<0.001	3.8	0.7	<0.001
Fibre (g/day)	20.9	1.6	24.3	1.3	-4.2	2.0	0.040	-3.5	1.9	0.080
Alcohol (% kcal/day)	1.6	0.4	1.2	0.6	0.7	0.4	0.104	0.6	0.5	0.181
Red meat (g/day)	43.6	5.3	28.9	4.3	16.1	7.3	0.035	14.4	6.7	0.038
Oily fish (g/day)	23.6	6.6	7.0	2.4	16.7	7.8	0.039	16.7	6.7	0.018
Fruit and vegetables (portions/day)	5.3	0.4	3.0	0.2	2.0	0.4	<0.001	2.1	0.4	<0.001

Unadjusted difference between diets assessed using paired t-test. Estimated marginal means and 95% confidence intervals computed from mixed-effects models adjusted for randomisation arm and night-shift status, with an interaction term for diet and randomisation arm, and a random effect for participant. Abbreviations: ITT: intention-to-treat; MPF: minimally processed food; PP: per-protocol; SE: standard error; UPF: ultra-processed food.

Supplementary Table 11: ITT: Unadjusted changes in self-reported dietary intake from baseline to week 4 and week 8 on each ad libitum diet.

ITT N = 50	MPF diet from baseline	UPF diet from baseline

	Week 4 (N = 34)			Week 8 (N = 33)			Week 4 (N = 43)			Week 8 (N = 36)		
	Mean	SE	p-value	Mean	SE	p-value	Mean	SE	p-value	Mean	SE	p-value
Total energy (kcal/day)	-551.3	117.1	<0.001	-438.7	154.6	0.008	-291.1	118.4	0.018	-329.2	728.8	0.010
Fat (% kcal/day)	-6.0	1.4	<0.001	-3.3	1.4	0.026	-8.5	1.2	<0.001	-6.4	6.6	<0.001
Saturated Fat (% kcal/day)	-3.0	0.5	<0.001	-2.6	0.8	0.003	-4.7	0.6	<0.001	-3.2	3.6	<0.001
Carbohydrate (% kcal/day)	2.1	1.5	0.183	-2.6	1.7	0.129	8.9	1.3	<0.001	6.8	8.0	<0.001
Total sugar (% kcal/day)	2.0	1.4	0.171	-1.2	1.4	0.392	1.3	1.0	0.219	-0.3	5.7	0.750
Total free sugar (% kcal/day)	-6.0	0.9	<0.001	-6.6	0.9	<0.001	1.1	0.8	0.199	-0.8	4.2	0.289
Salt (g/day)	-2.8	0.3	<0.001	-2.8	0.5	<0.001	-1.7	0.4	<0.001	-1.5	2.5	0.001
Protein (% kcal/day)	4.3	0.7	<0.001	4.7	0.8	<0.001	0.7	0.6	0.239	0.4	3.8	0.517
Fibre (g/day)	0.4	1.6	0.780	1.8	1.8	0.329	6.3	1.3	<0.001	4.2	10.6	0.023
Alcohol (% kcal/day)	-0.3	0.5	0.514	1.3	1.1	0.253	-1.2	0.5	0.010	-0.9	4.0	0.174
Red meat (g/day)	-3.8	9.6	0.696	-13.9	13.3	0.306	-32.1	9.1	0.001	-29.3	61.7	0.007
Oily fish (g/day)	11.4	6.1	0.073	17.2	8.6	0.054	-0.5	2.9	0.860	0.1	20.2	0.971
Fruit and vegetables (portions/day)	1.6	0.5	0.003	1.3	0.5	0.012	-0.6	0.4	0.133	-0.7	2.3	0.105

Unadjusted change from baseline assessed using paired t-test. Abbreviations: ITT: intention-to-treat; MPF: minimally processed food; SE: standard error; UPF: ultra-processed food.

Supplementary Table 12: Unadjusted changes in self-reported dietary intake from baseline on each ad libitum diet (week 4 and week 8 average), and unadjusted and adjusted differences in changes in dietary intake from baseline between ad libitum diets for ITT and PP.

ITT N = 50	MPF diet from baseline (N = 39)	UPF diet from baseline (N = 45)	MPF - UPF diet from baseline (N = 36) Unadjusted	MPF - UPF diet from baseline (N = 48) Adjusted

	Mean	SE	p-value	Mean	SE	p-value	Mean	SE	p-value	Mean	SE	p-value
Total energy (kcal/day)	-503.7	130.2	<0.001	-289.6	102.8	0.007	-283.6	185.2	0.135	-259.2	157.7	0.108
Fat (% kcal/day)	-4.6	1.1	<0.001	-7.3	1.0	<0.001	3.7	1.7	0.043	2.6	1.5	0.088
Saturated Fat (% kcal/day)	-2.9	0.5	<0.001	-4.2	0.5	<0.001	1.3	0.8	0.125	1.1	0.7	0.121
Carbohydrate (% kcal/day)	-0.4	1.2	0.734	7.9	1.1	<0.001	-9.1	2.0	<0.001	-8.5	1.7	<0.001
Total sugar (% kcal/day)	0.3	1.3	0.821	0.8	0.9	0.418	-0.7	1.8	0.710	-0.4	1.6	0.816
Total free sugar (% kcal/day)	-6.6	0.8	<0.001	0.5	0.7	0.470	-7.0	1.2	<0.001	-7.3	1.1	<0.001
Salt (g/day)	-2.7	0.4	<0.001	-1.5	0.4	<0.001	-1.4	0.5	0.005	-1.4	0.4	0.001
Protein (% kcal/day)	5.0	0.7	<0.001	0.5	0.5	0.373	4.3	1.0	<0.001	4.8	0.9	<0.001
Fibre (g/day)	1.1	1.6	0.489	5.5	1.3	<0.001	-4.9	2.3	0.040	-4.8	2.0	0.021
Alcohol (% kcal/day)	0.0	0.4	0.920	-1.2	0.5	0.020	1.3	0.7	0.067	1.2	0.6	0.068
Red meat (g/day)	-5.0	11.5	0.667	-29.9	8.0	0.001	21.9	13.2	0.105	24.8	12.5	0.055
Oily fish (g/day)	12.6	5.6	0.029	-0.5	2.8	0.867	13.8	7.2	0.065	11.9	6.0	0.054
Fruit and vegetables (portions/day)	1.5	0.4	0.001	-0.6	0.3	0.099	2.0	0.5	<0.001	2.1	0.5	<0.001

**PP N = 43**	**MPF diet from baseline (N = 35)**			**UPF diet from baseline (N = 41)**			**MPF - UPF diet from baseline (N = 34) Unadjusted**			**MPF - UPF diet from baseline (N = 42) adjusted**		
	**Mean**	**SE**	**p-value**	**Mean**	**SE**	**p-value**	**Mean**	**SE**	**p-value**	**Mean**	**SE**	**p-value**
Total energy (kcal/day)	-485.2	132.2	0.001	-250.5	107.1	0.024	-302.8	194.0	0.128	-260.1	160.4	0.113
Fat (% kcal/day)	-5.2	1.1	<0.001	-7.6	1.0	<0.001	2.6	1.7	0.125	2.3	1.5	0.129
Saturated Fat (% kcal/day)	-2.8	0.5	<0.001	-4.1	0.5	<0.001	1.1	0.8	0.184	1.2	0.7	0.091
Carbohydrate (% kcal/day)	0.4	1.2	0.734	8.4	1.1	<0.001	-8.2	2.0	<0.001	-8.0	1.6	<0.001
Total sugar (% kcal/day)	1.1	1.2	0.370	0.5	1.0	0.594	0.5	1.7	0.779	0.7	1.6	0.676
Total free sugar (% kcal/day)	-6.1	0.8	<0.001	0.5	0.8	0.500	-6.4	1.2	<0.001	-6.6	1.1	<0.001
Salt (g/day)	-2.7	0.4	<0.001	-1.3	0.4	0.001	-1.5	0.5	0.003	-1.4	0.4	0.001
Protein (% kcal/day)	4.7	0.8	<0.001	0.4	0.6	0.535	4.2	1.1	<0.001	4.5	0.9	<0.001
Fibre (g/day)	1.6	1.7	0.356	5.6	1.4	<0.001	-4.5	2.4	0.070	-4.2	2.1	0.054
Alcohol (% kcal/day)	0.0	0.5	0.921	-1.3	0.5	0.018	1.5	0.7	0.046	1.3	0.7	0.060
Red meat (g/day)	-10.6	10.1	0.302	-29.1	8.5	0.001	18.3	12.7	0.159	19.2	12.5	0.132
Oily fish (g/day)	14.6	6.1	0.022	-0.5	3.1	0.867	14.6	7.6	0.065	14.6	6.5	0.031
Fruit and vegetables (portions/day)	1.7	0.5	0.001	-0.7	0.3	0.045	2.2	0.5	<0.001	2.4	0.5	<0.001

Unadjusted change from baseline and between diets assessed using paired t-test. Estimated marginal means and 95% confidence intervals computed from mixed-effects models adjusted for randomisation arm and night-shift status, with an interaction term for diet and randomisation arm, and a random effect for participant. Abbreviations: ITT: intention-to-treat; MPF: minimally processed food; PP: per-protocol; SE: standard error; UPF: ultra-processed food.

Supplementary Table 13: Self-reported energy intake at each timepoint by randomisation arm and period for ITT and PP.

ITT N = 50	Randomisation arm	N		Total energy (kcal/day)

			Mean number of recalls per participant:	Mean	SE
Baseline period 1	MPF / UPF (N = 23)	23	4.0	2108.4	113.3
Ad libitum diet period 1 (MPF)		20	3.0	1402.8	134.9
Baseline period 2		21	1.7	1714.1	111.9
Ad libitum diet period 2 (UPF)		18	2.8	1773.4	121.0
Baseline period 1	UPF / MPF (N = 27)	27	4.0	2238.8	143.6
Ad libitum diet period 1 (UPF)		27	3.3	1725.6	71.5
Baseline period 2		27	1.8	1792.9	119.1
Ad libitum diet period 2 (MPF)		19	3.1	1438.9	128.4
**PP N = 43**	**Diet**	**N**	**Mean number of recalls per participant:**	**Total energy (kcal/day)**<br>**Mean**	**SE**
Baseline period 1	MPF / UPF (N = 20)	20	4.0	2129.1	129.9
Ad libitum diet period 1 (MPF)		18	3.0	1438.8	147.5
Baseline period 2		20	1.7	1722.9	117.2
Ad libitum diet period 2 (UPF)		18	2.8	1773.4	121.0
Baseline period 1	UPF / MPF (N = 23)	23	4.0	2196.2	167.1
Ad libitum diet period 1 (UPF)		23	3.4	1713.6	72.2
Baseline period 2		23	1.8	1737.4	122.1
Ad libitum diet period 2 (MPF)		17	3.2	1425.5	130.4

Abbreviations: ITT: intention-to-treat; MPF: minimally processed food; PP: per-protocol; SE: standard error; UPF: ultra-processed food.

Supplementary Table 14: Diet ratings overall.

	MPF diet (N = 40)		UPF diet (N = 41)		MPF diet - UPF diet (N = 40)			
	Mean	SE	Mean	SE	Mean	Lower 95%CI	Upper 95%CI	p-value
Diet overall	6.23	0.3	6.20	0.3	0.03	-0.99	1.04	0.961
Meals and snacks	6.18	0.3	6.22	0.3	0.00	-0.82	0.82	1.000
Flavours and taste	5.18	0.3	6.29	0.3	-1.08	-1.99	-0.16	0.022
Textures	5.80	0.3	6.20	0.4	-0.35	-1.37	0.67	0.491
Portion sizes	8.28	0.3	8.68	0.3	-0.40	-1.03	0.23	0.210
Delivery and preparation	7.08	0.5	8.56	0.3	-1.60	-2.78	-0.42	0.009
Hunger level	7.63	0.2	8.15	0.2	-0.55	-1.19	0.09	0.088
Contentment level	6.04	0.4	6.29	0.3	-0.24	-1.14	0.66	0.597
Diet sustainability	5.40	0.5	4.71	0.5	0.68	-0.58	1.93	0.283

Unadjusted differences between diets assessed using paired t-test. Abbreviations: 95%CI: 95% confidence interval; MPF: minimally processed food; SE: standard error ; UPF: ultra-processed food.

Supplementary Table 15: Diet ratings by randomisation arm.

	MPF diet				UPF diet				MPF diet first - UPF diet first (N = 41)			
	Arm MPF/UPF - first (N = 17)		Arm UPF/MPF - second (N = 16)		Arm MPF/UPF - second (N = 24)		Arm UPF/MPF - first (N = 24)					
	**Mean**	**SE**	**Mean**	**SE**	**Mean**	**SE**	**Mean**	**SE**	**Mean**	**Lower 95%CI**	**Upper 95%CI**	**p-value**
Diet overall	6.71	0.4	5.87	0.6	6.47	0.5	6.00	0.4	0.71	-0.45	1.87	0.226
Meals and snacks	6.47	0.3	5.96	0.5	6.53	0.4	6.00	0.4	0.47	-0.65	1.59	0.399
Flavours and taste	5.29	0.5	5.09	0.5	6.94	0.4	5.83	0.4	-0.54	-1.87	0.79	0.416
Textures	6.29	0.6	5.43	0.4	6.65	0.4	5.88	0.5	0.42	-1.15	1.99	0.593
Portion sizes	8.12	0.4	8.39	0.5	8.12	0.5	9.08	0.3	-0.97	-2.03	0.10	0.074
Delivery and preparation	7.41	0.7	6.83	0.8	8.12	0.4	8.88	0.3	-1.46	-3.08	0.16	0.074
Hunger level	7.94	0.4	7.39	0.4	7.76	0.3	8.42	0.3	-0.48	-1.41	0.46	0.310
Contentment level	6.24	0.6	5.89	0.6	6.41	0.4	6.21	0.4	0.03	-1.33	1.39	0.968
Diet sustainability	6.24	0.8	4.78	0.6	5.65	0.6	4.04	0.7	2.19	0.10	4.28	0.040

Unadjusted differences between diets assessed using two-sample t-test. Abbreviations: 95%CI: 95% confidence interval; MPF: minimally processed food; SE: standard error; UPF: ultra-processed food.

Supplementary Table 16: Unadjusted and adjusted changes in physical activity from baseline to week 8 on each diet for ITT and PP.

	MPF diet				UPF diet				MPF diet - UPF diet				MPF diet			UPF diet			MPF diet - UPF diet		
**ITT**	**N**	**Mean**	**SE**	**p-value**	**N**	**Mean**	**SE**	**p-value**	**N**	**Mean**	**SE**	**p-value**	**Mean**	**Lower 95%CI**	**Upper 95%CI**	**Mean**	**Lower 95%CI**	**Upper 95%CI**	**Mean**	**SE**	**p-value**
Average daily MVPA (minutes) (N= 43)	36	-0.55	2.5	0.827	37	2.04	3.2	0.532	30	-0.51	4.4	0.909	-1.11	-8.41	6.19	1.74	-5.34	8.81	-2.85	4.13	0.495
**PP**																					
Average daily MVPA (minutes) (N= 40)	35	-1.10	2.5	0.664	35	2.48	3.2	0.451	30	-0.51	4.4	0.909	-1.66	-8.88	5.56	2.01	-5.01	9.04	-3.67	4.15	0.382

On average, participants wore the device for 6.3 days, with an average 17 hours and 54 minutes hours wear time on each day, and worn for all 7 days in 51% (88/173) of participant measurements. Seven participants (eight measurements) were excluded from the analysis for insufficient wear time (at least four days with at least 10 hours of daily wear time). Unadjusted change from baseline on each diet assessed using paired t-test. Estimated marginal means and 95% confidence intervals computed from mixed-effects models adjusted for randomisation arm and night-shift status, with an interaction term for diet and randomisation arm, and a random effect for participant. Abbreviations: 95%CI: 95% confidence interval; ITT: intention-to-treat; MPF: minimally processed food; MVPA: moderate to vigorous physical activity; SE: standard error; UPF: ultra-processed food.

Supplementary Table 17: ITT: Changes in waist-to-height ratio and estimated energy imbalance from baseline to week 8 on each diet, and differences in changes from baseline to week 8 between diets.

ITT N = 50	MPF diet			UPF diet			MPF diet - UPF diet		
	**Mean**	**Lower 95%CI**	**Upper 95%CI**	**Mean**	**Lower 95%CI**	**Upper 95%CI**	**Mean**	**SE**	**p-value**
Waist-to-height ratio	-0.01	-0.02	0.00	0.00	-0.01	0.01	-0.01	0.01	0.156
Estimated daily energy imbalance (kcal/day)	-289.90	-423.67	-156.12	-119.52	-251.74	12.71	-170.38	57.92	0.005

Estimated marginal means and 95% confidence intervals computed from mixed-effects models adjusted for randomisation arm and night-shift status, with an interaction term for diet and randomisation arm, and a random effect for participant. Abbreviations: 95%CI: 95% confidence interval; ITT: intention-to-treat; MPF: minimally processed food; SE: standard error; UPF: ultra-processed food.

Supplementary Table 18: Intention-to-treat: Unadjusted changes in outcomes from baseline to week 4 on each diet, and unadjusted differences in changes in outcomes from baseline to week 4 between diets.

ITT N = 50	MPF diet				UPF diet				MPF diet - UPF diet			
	N	Mean	SE	p-value	N	Mean	SE	p-value	N	Mean	SE	p-value
Percentage weight change (%)	46	-1.55	0.3	<0.001	47	-0.91	0.2	<0.001	44	-0.51	0.3	0.114
Weight (kg)	46	-1.45	0.3	<0.001	47	-0.87	0.2	<0.001	44	-0.46	0.3	0.116
BMI (kg/m^2)	46	-0.52	0.1	<0.001	47	-0.31	0.1	<0.001	44	-0.17	0.1	0.123
Waist circumference (cm)	45	0.44	0.6	0.483	47	0.03	0.6	0.961	43	0.70	1.1	0.518
Waist-to-height ratio	45	0.00	0.0	0.467	47	0.00	0.0	0.982	43	0.00	0.0	0.498
Fat mass (kg)	44	-1.39	0.3	<0.001	47	-0.93	0.2	<0.001	42	-0.24	0.3	0.420
Body fat percentage (%)	44	-0.92	0.2	<0.001	47	-0.64	0.2	0.004	42	-0.08	0.3	0.769
Visceral fat rating	44	-0.52	0.1	<0.001	47	-0.26	0.1	0.002	42	-0.21	0.1	0.130
Fat-free mass (kg)	44	-0.15	0.1	0.305	47	0.06	0.2	0.741	42	-0.28	0.2	0.246
Muscle mass (kg)	44	-0.14	0.1	0.303	47	0.06	0.2	0.738	42	-0.27	0.2	0.245
Bone mass (kg)	44	-0.01	0.0	0.473	47	0.00	0.0	0.844	42	-0.01	0.0	0.376
Total body water (kg)	44	-0.27	0.1	0.036	47	0.15	0.2	0.325	42	-0.35	0.1	0.019
Total body water percentage (%)	44	0.41	0.1	0.005	47	0.57	0.1	<0.001	42	-0.15	0.1	0.299
SBP (mmHg)	46	-11.95	1.4	<0.001	47	-7.23	1.8	<0.001	44	-4.89	2.3	0.037
DBP (mmHg)	46	6.61	1.4	<0.001	47	8.41	1.3	<0.001	44	-2.61	1.7	0.123
HR (beats per minute)	45	1.31	1.9	0.494	47	-0.28	1.6	0.865	44	1.91	2.4	0.438
PFS food available	41	-0.59	0.1	<0.001	37	-0.50	0.1	<0.001	32	-0.02	0.2	0.908
PFS food present	41	-0.69	0.2	<0.001	37	-0.59	0.1	<0.001	32	0.04	0.2	0.855
PFS food tasted	41	-0.30	0.1	0.032	37	-0.52	0.1	<0.001	32	0.23	0.2	0.207
PFS total	41	-0.53	0.1	<0.001	37	-0.53	0.1	<0.001	32	0.08	0.2	0.628
CoEQ craving control	41	21.89	3.7	<0.001	37	16.90	3.2	<0.001	32	3.30	4.5	0.469
CoEQ craving for sweet	41	-16.33	2.6	<0.001	37	-14.75	2.1	<0.001	32	-2.39	2.9	0.408
CoEQ craving for savoury	41	-13.06	2.9	<0.001	37	-10.01	2.9	0.002	32	7.02	3.6	0.060

CoEQ positive mood	41	2.45	2.2	0.272	37	4.93	2.4	0.049	32	-3.89	2.8	0.177
CoEQ control over craved nominated food	41	-27.95	5.4	<0.001	37	-20.24	5.1	<0.001	32	-9.47	6.7	0.166

Unadjusted change from baseline and between diets assessed using paired t-test. Abbreviations: BMI: body mass index; CoEQ: Control of Eating Questionnaire; DBP: diastolic blood pressure; HR: heart rate; ITT: intention-to-treat; MPF: minimally processed food; PFS: Power of Food Scale; SBP: systolic blood pressure; SE: standard error; UPF: ultra-processed food. Biomarker data not collected at week 4.

Supplementary Table 19: Intention-to-treat: Repeated measures mixed-effects analysis of adjusted differences in changes in outcomes from baseline to week 4 and baseline to week 8 between diets.

ITT N = 50	MPF diet - UPF diet					
	Week 4 difference			Week 8 difference		
	Mean	SE	p-value	Mean	SE	p-value
Percentage weight change (%)	-0.65	0.32	0.045	-0.99	0.32	0.002
Weight (kg)	-0.60	0.30	0.047	-0.95	0.29	0.002
BMI (kg/m^2)	-0.21	0.11	0.048	-0.33	0.11	0.002
Waist circumference (cm)	0.26	0.75	0.728	-1.25	0.76	0.100
Waist-to-height ratio	0.00	0.00	0.701	-0.01	0.00	0.105
Fat mass (kg)	-0.41	0.27	0.127	-0.99	0.26	<0.001
Body fat percentage (%)	-0.24	0.23	0.294	-0.75	0.23	0.001
Visceral fat rating	-0.27	0.11	0.016	-0.40	0.11	<0.001
Fat-free mass (kg)	-0.21	0.21	0.302	0.00	0.20	0.986
Muscle mass (kg)	-0.20	0.20	0.299	-0.01	0.19	0.950
Bone mass (kg)	-0.01	0.01	0.466	0.01	0.01	0.465
Total body water (kg)	-0.42	0.13	0.002	-0.49	0.13	<0.001
Total body water percentage (%)	-0.18	0.14	0.186	-0.07	0.13	0.589
SBP (mmHg)	-4.91	1.88	0.009	-2.98	1.87	0.112
DBP (mmHg)	-1.87	1.27	0.144	-1.80	1.27	0.157
HR (beats per minute)	1.55	1.85	0.403	0.82	1.84	0.655
PoF Food available	-0.12	0.14	0.397	-0.23	0.14	0.104
PoF Food present	-0.12	0.15	0.416	-0.21	0.16	0.189
PoF Food tasted	0.20	0.13	0.120	-0.15	0.13	0.254
PoF Total	-0.02	0.12	0.901	-0.19	0.12	0.118
CoEQ Craving control	5.45	3.58	0.129	10.07	3.64	0.006
CoEQ Craving for sweet	-2.16	2.50	0.388	-6.20	2.55	0.016
CoEQ Craving for savoury	-3.96	2.99	0.187	-9.05	3.06	0.004

CoEQ Positive Mood	-3.01	2.34	0.200	-3.56	2.38	0.136
CoEQ control over craved nominated food	-9.03	5.22	0.085	-11.60	5.30	0.030

Estimated marginal means and 95% confidence intervals computed from mixed-effects models adjusted for randomisation arm and night-shift status, with an interaction term for diet and randomisation arm, and a random effect for participant with week 4 and week 8 data. Abbreviations: 95%CI: 95% confidence interval; BMI: body mass index; CoEQ: Control of Eating Questionnaire; DBP: diastolic blood pressure; HR: heart rate; MPF: minimally processed food; PFS: Power of Food Scale; SBP: systolic blood pressure; SE: standard error; UPF: ultra-processed food. Biomarker data not collected at week 4.

Supplementary Table 20: Per-protocol: Unadjusted changes in outcomes from baseline to week 8 on each diet, and unadjusted differences in changes in outcomes from baseline to week 8 between diets.

PP N = 43	MPF diet				UPF diet				MPF diet - UPF diet			
	N	Mean	SE	p-value	N	Mean	SE	p-value	N	Mean	SE	p-value
Percentage weight change (%)	43	-2.57	0.5	<0.001	43	-1.79	0.4	<0.001	43	-0.79	0.5	0.140
Weight (kg)	43	-2.39	0.4	<0.001	43	-1.61	0.3	<0.001	43	-0.78	0.5	0.115
BMI (kg/m^2)	43	-0.85	0.2	<0.001	43	-0.58	0.1	<0.001	43	-0.27	0.2	0.128
Waist circumference (cm)	42	-1.92	0.8	0.022	42	-0.89	0.8	0.288	41	-0.66	1.2	0.586
Waist-to-height ratio	42	-0.01	0.0	0.022	42	-0.01	0.0	0.273	41	0.00	0.0	0.592
Fat mass (kg)	42	-1.83	0.3	<0.001	43	-1.14	0.3	<0.001	42	-0.67	0.4	0.069
Body fat percentage (%)	42	-1.06	0.2	<0.001	43	-0.59	0.2	0.002	42	-0.45	0.3	0.091
Visceral fat rating	42	-0.62	0.1	<0.001	43	-0.33	0.1	<0.001	42	-0.31	0.2	0.062
Fat-free mass (kg)	42	-0.65	0.3	0.015	43	-0.47	0.1	0.001	42	-0.17	0.3	0.543
Muscle mass (kg)	42	-0.61	0.2	0.016	43	-0.44	0.1	0.001	42	-0.16	0.3	0.532
Bone mass (kg)	42	-0.03	0.0	0.018	43	-0.03	0.0	0.001	42	0.00	0.0	0.875
Total body water (kg)	42	-0.91	0.1	<0.001	43	-0.40	0.2	0.012	42	-0.54	0.2	0.002
Total body water percentage (%)	42	0.18	0.2	0.262	43	0.34	0.1	0.015	42	-0.21	0.2	0.198
SBP (mmHg)	43	-5.09	1.8	0.006	43	-2.06	2.0	0.321	43	-3.03	2.5	0.239
DBP (mmHg)	43	-3.77	1.3	0.005	43	-1.34	0.8	0.116	43	-2.43	1.4	0.101
HR (beats per minute)	42	-2.52	1.5	0.106	43	-4.77	1.8	0.013	42	1.17	2.4	0.627
Bilirubin (umol/L)	39	0.08	0.4	0.860	41	0.59	0.5	0.259	37	-0.62	0.5	0.246
Alkaline phosphatase (IU/L)	33	1.21	0.9	0.204	37	-0.41	1.9	0.829	28	-0.75	1.9	0.699
Alanine transaminase (IU/L)	32	2.91	2.8	0.315	36	1.14	1.9	0.542	26	4.62	3.1	0.149
Albumin (g/L)	41	0.32	0.4	0.384	41	0.51	0.4	0.164	39	0.26	0.5	0.634
HbA1C (%)	43	-0.08	0.0	0.035	43	-0.03	0.0	0.246	43	-0.05	0.0	0.306
Fasting glucose (mmol/L)	43	-0.08	0.1	0.213	42	-0.16	0.0	0.003	42	0.07	0.1	0.349
Total cholesterol (mmol/L)	38	-0.22	0.1	0.007	39	-0.40	0.1	<0.001	34	0.15	0.1	0.245
Total cholesterol:HDL ratio	38	0.09	0.1	0.173	39	-0.04	0.1	0.529	34	0.16	0.1	0.196

HDL-C (mmol/L)	40	-0.15	0.0	0.001	41	-0.11	0.0	<0.001	38	-0.04	0.1	0.395
LDL-C (mmol/L)	38	-0.06	0.1	0.375	39	-0.33	0.1	<0.001	34	0.27	0.1	0.021
Non-HDL-C (mmol/L)	38	-0.10	0.1	0.172	39	-0.29	0.1	<0.001	34	0.19	0.1	0.117
Triglycerides (mmol/L)	38	-0.09	0.1	0.112	39	0.09	0.0	0.047	34	-0.19	0.1	0.015
C-reactive protein (mg/L)	41	-0.51	0.3	0.070	40	-0.37	0.6	0.515	38	-0.23	0.7	0.749
PoF Food available	35	-0.55	0.2	0.003	34	-0.35	0.2	0.037	32	-0.12	0.2	0.583
PoF Food present	35	-0.66	0.2	0.001	34	-0.47	0.2	0.010	32	-0.05	0.2	0.807
PoF Food tasted	35	-0.38	0.2	0.022	34	-0.25	0.1	0.037	32	-0.14	0.2	0.423
PoF Total	35	-0.53	0.2	0.002	34	-0.36	0.1	0.010	32	-0.11	0.2	0.550
CoEQ Craving control	35	21.03	4.3	<0.001	34	14.28	4.1	0.001	32	5.88	5.7	0.311
CoEQ Craving for sweet	35	-16.95	2.7	<0.001	34	-11.90	2.4	<0.001	32	-4.91	4.0	0.224
CoEQ Craving for savoury	35	-14.20	3.4	<0.001	33	-5.89	2.8	0.041	32	-6.93	4.8	0.155
CoEQ Positive Mood	35	-0.16	2.7	0.952	34	3.93	2.8	0.165	32	-3.15	3.2	0.336
CoEQ control over craved nominated food	35	-25.83	5.9	<0.001	34	-16.18	6.3	0.015	32	-9.59	7.3	0.196

Unadjusted change from baseline and between diets assessed using paired t-test. Abbreviations: BMI: body mass index; CoEQ: Control of Eating Questionnaire; CRP: c-reactive protein; DBP: diastolic blood pressure; HbA1c: glycated haemoglobin; HDL-C: high-density lipoprotein cholesterol; HR: heart rate; LDL-C: low-density lipoprotein cholesterol; MPF: minimally processed food; Non-HDL-C: Non-high-density lipoprotein cholesterol; PFS: Power of Food Scale; PP: per-protocol; SBP: systolic blood pressure; SE: standard error; UPF: ultra-processed food.

Supplementary Table 21: Per-protocol: Changes in outcomes from baseline to week 8 on each diet, and differences in changes in outcomes from baseline to week 8 between diets.

PP N = 43	MPF diet			UPF diet			MPF diet - UPF diet		
	Mean	Lower 95%CI	Upper 95%CI	Mean	Lower 95%CI	Upper 95%CI	Mean	SE	p-value
Percentage weight change (%)	-2.06	-3.01	-1.11	-1.16	-2.11	-0.21	-0.91	0.46	0.055
Weight (kg)	-1.84	-2.70	-0.98	-0.95	-1.81	-0.09	-0.89	0.42	0.040
BMI (kg/m2)	-0.67	-0.98	-0.36	-0.36	-0.66	-0.05	-0.31	0.15	0.046
Waist circumference (cm)	-1.37	-3.25	0.51	-0.04	-1.88	1.79	-1.33	1.09	0.230
Waist-to-height ratio	-0.01	-0.02	0.00	0.00	-0.01	0.01	-0.01	0.01	0.242
Fat mass (kg)	-1.45	-2.11	-0.78	-0.69	-1.35	-0.02	-0.76	0.28	0.011
Body fat percentage (%)	-0.92	-1.42	-0.42	-0.41	-0.91	0.09	-0.51	0.23	0.031
Visceral fat rating	-0.51	-0.75	-0.27	-0.19	-0.43	0.05	-0.32	0.15	0.033
Fat-free mass (kg)	-0.44	-0.91	0.04	-0.25	-0.72	0.22	-0.19	0.27	0.496
Muscle mass (kg)	-0.42	-0.87	0.04	-0.23	-0.69	0.22	-0.18	0.26	0.486
Bone mass (kg)	-0.02	-0.05	0.01	-0.02	-0.04	0.01	0.00	0.02	0.824
Total body water (kg)	-0.68	-1.00	-0.37	-0.14	-0.46	0.17	-0.54	0.15	0.001
Total body water percentage (%)	0.17	-0.20	0.54	0.34	-0.03	0.71	-0.17	0.16	0.305
SBP (mmHg)	-6.28	-10.90	-1.71	-2.93	-7.50	1.65	-3.36	2.47	0.183
DBP (mmHg)	-4.02	-6.54	-1.50	-1.57	-4.09	0.95	-2.45	1.47	0.103
HR (beats per minute)	-2.86	-6.94	1.23	-5.03	-9.10	-0.97	2.18	2.44	0.377
Bilirubin (umol/L)	0.23	-0.98	1.43	0.75	-0.43	1.93	-0.52	0.49	0.295
Alkaline phosphatase (IU/L)	0.45	-3.22	4.12	-1.24	-4.72	2.23	1.69	2.20	0.447
Alanine transaminase (IU/L)	1.97	-3.82	7.75	-0.51	-6.03	5.02	2.47	2.85	0.391
Albumin (g/L)	-0.27	-1.12	0.58	-0.05	-0.88	0.78	-0.23	0.50	0.652
HbA1C (%)	-0.08	-0.15	-0.01	-0.02	-0.09	0.05	-0.06	0.04	0.184
Fasting glucose (mmol/L)	-0.12	-0.26	0.02	-0.20	-0.33	-0.06	0.07	0.08	0.329
Total cholesterol (mmol/L)	-0.24	-0.42	-0.06	-0.42	-0.60	-0.24	0.18	0.11	0.107

Total cholesterol:HDL ratio	0.00	-0.16	0.17	-0.13	-0.29	0.03	0.13	0.10	0.177
HDL-C (mmol/L)	-0.11	-0.19	-0.03	-0.08	-0.16	0.00	-0.03	0.05	0.513
LDL-C (mmol/L)	-0.09	-0.25	0.07	-0.36	-0.51	-0.20	0.27	0.09	0.007
Non-HDL-C (mmol/L)	-0.15	-0.32	0.02	-0.34	-0.50	-0.18	0.19	0.10	0.063
Triglycerides (mmol/L)	-0.13	-0.25	-0.01	0.05	-0.06	0.17	-0.18	0.07	0.009
C-reactive protein (mg/L)	-0.65	-1.72	0.43	-0.48	-1.54	0.58	-0.16	0.63	0.801
PoF Food available	-0.38	-0.87	0.10	-0.08	-0.57	0.40	-0.30	0.19	0.124
PoF Food present	-0.72	-1.25	-0.20	-0.41	-0.94	0.11	-0.31	0.18	0.097
PoF Food tasted	-0.34	-0.77	0.08	-0.15	-0.58	0.28	-0.20	0.17	0.266
PoF Total	-0.49	-0.92	-0.05	-0.22	-0.65	0.22	-0.27	0.15	0.078
CoEQ Craving control	23.33	11.11	35.55	13.13	0.84	25.42	10.20	4.91	0.046
CoEQ Craving for sweet	-10.57	-17.54	-3.61	-4.00	-11.01	3.01	-6.58	3.50	0.069
CoEQ Craving for savoury	-14.14	-22.89	-5.39	-3.76	-12.56	5.04	-10.38	4.41	0.025
CoEQ Positive Mood	-2.22	-10.81	6.36	2.10	-6.53	10.73	-4.32	3.26	0.194
CoEQ control over craved nominated food	-30.44	-49.36	-11.53	-16.78	-35.79	2.23	-13.66	6.66	0.048

Estimated marginal means and 95% confidence intervals computed from mixed-effects models adjusted for randomisation arm and night-shift status, with an interaction term for diet and randomisation arm, and a random effect for participant. Abbreviations: 95%CI: 95% confidence interval; BMI: body mass index; CoEQ: Control of Eating Questionnaire; CRP: c-reactive protein; DBP: diastolic blood pressure; HbA1c: glycated haemoglobin; HDL-C: *high-density lipoprotein cholesterol; HR: heart rate;* LDL-C: *low-density lipoprotein cholesterol; MPF: minimally processed food; Non-HDL-C: Non-high-density lipoprotein cholesterol; PFS: Power of Food Scale; PP: per-protocol; SBP: systolic blood pressure; SE: standard error; UPF: ultra-processed food.*

Supplementary Table 22: Per-protocol: Unadjusted changes in outcomes from baseline to week 8 on each diet by randomisation arm, and unadjusted and adjusted differences in changes in outcomes from baseline to week 8 between first diets.

PP N=43	MPF diet								UPF diet								MPF diet first - UPF diet first							
	Arm MPF/UPF - first				Arm UPF/MPF - second				Arm MPF/UPF - second				Arm UPF/MPF - first				Unadjusted					Mixed-effects model		
	N	Mean	SE	p	N	Mean	SE	p	N	Mean	SE	p	N	Mean	SE	p	N	Mean	Lower 95%CI	Upper 95%CI	p	Mean	SE	p
Percentage weight change (%)	20	-3.95	0.7	<0.001	23	-1.37	0.6	0.025	20	-1.34	0.5	0.014	23	-2.17	0.5	<0.001	43	-1.79	-3.42	-0.15	0.033	-1.74	0.77	0.026
Weight (kg)	20	-3.77	0.6	<0.001	23	-1.19	0.5	0.024	20	-1.25	0.4	0.012	23	-1.93	0.5	<0.001	43	-1.84	-3.40	-0.28	0.022	-1.79	0.70	0.012
BMI (kg/m2)	20	-1.34	0.2	<0.001	23	-0.43	0.2	0.024	20	-0.44	0.2	0.013	23	-0.71	0.2	<0.001	43	-0.63	-1.18	-0.08	0.027	-0.61	0.25	0.016
Waist circumference (cm)	19	-4.16	1.3	0.004	23	-0.07	0.9	0.942	19	1.00	1.2	0.424	23	-2.46	1.0	0.028	42	-1.70	-4.96	1.56	0.298	-1.56	1.55	0.316
Waist-to-height ratio	19	-0.02	0.0	0.003	23	0.00	0.0	0.933	19	0.01	0.0	0.444	23	-0.01	0.0	0.027	42	-0.01	-0.03	0.01	0.305	-0.01	0.01	0.327
Fat mass (kg)	20	-2.82	0.4	<0.001	22	-0.94	0.4	0.019	20	-0.66	0.3	0.067	23	-1.56	0.4	0.001	43	-1.26	-2.42	-0.10	0.035	-1.23	0.52	0.022
Body fat percentage (%)	20	-1.58	0.3	<0.001	22	-0.59	0.4	0.123	20	-0.22	0.2	0.396	23	-0.92	0.2	0.001	43	-0.66	-1.36	0.04	0.065	-0.65	0.40	0.109
Visceral fat rating	20	-1.00	0.2	<0.001	22	-0.27	0.1	0.083	20	-0.20	0.1	0.104	23	-0.43	0.1	0.002	43	-0.57	-1.01	-0.12	0.015	-0.56	0.20	0.008
Fat-free mass (kg)	20	-0.95	0.3	0.001	22	-0.37	0.4	0.391	20	-0.59	0.2	0.015	23	-0.37	0.2	0.024	43	-0.58	-1.19	0.03	0.060	-0.56	0.40	0.157
Muscle mass (kg)	20	-0.90	0.2	0.002	22	-0.35	0.4	0.393	20	-0.55	0.2	0.016	23	-0.35	0.1	0.028	43	-0.55	-1.11	0.01	0.054	-0.54	0.38	0.158
Bone mass (kg)	20	-0.05	0.0	0.002	22	-0.02	0.0	0.427	20	-0.04	0.0	0.017	23	-0.02	0.0	0.022	43	-0.03	-0.06	0.00	0.081	-0.03	0.02	0.216
Total body water (kg)	20	-1.20	0.2	<0.001	22	-0.64	0.1	<0.001	20	-0.26	0.2	0.273	23	-0.52	0.2	0.018	43	-0.68	-1.23	-0.14	0.016	-0.67	0.25	0.010
Total body water percentage (%)	20	0.44	0.2	0.072	22	-0.06	0.2	0.777	20	0.36	0.2	0.132	23	0.32	0.2	0.053	43	0.12	-0.43	0.67	0.654	0.12	0.29	0.676
SBP (mmHg)	20	-9.80	2.2	<0.001	23	-1.00	2.5	0.688	20	-1.85	2.9	0.533	23	-2.24	2.9	0.453	43	-7.56	-15.10	-0.02	0.049	-7.62	3.78	0.047
DBP (mmHg)	20	-5.80	1.5	0.001	23	-2.00	1.9	0.306	20	-3.10	1.3	0.025	23	0.20	1.0	0.848	43	-6.00	-9.62	-2.37	0.002	-6.00	2.12	0.006
HR (beats per minute)	20	-2.60	1.7	0.148	22	-2.45	2.5	0.337	20	-3.75	2.3	0.117	23	-5.65	2.8	0.058	43	3.05	-3.67	9.78	0.363	3.03	3.43	0.380
Bilirubin (umol/L)	20	-0.05	0.6	0.939	19	0.21	0.6	0.723	20	1.65	0.8	0.043	21	-0.43	0.6	0.500	41	0.38	-1.44	2.19	0.676	0.27	0.93	0.776
Alkaline phosphatase (IU/L)	18	0.61	1.3	0.652	15	1.93	1.3	0.166	19	0.79	2.0	0.693	18	-1.67	3.3	0.615	36	2.28	-5.00	9.56	0.524	2.13	3.06	0.488
Alanine transaminase (IU/L)	17	4.65	4.5	0.322	15	0.93	3.3	0.781	19	1.21	3.0	0.690	17	1.06	2.2	0.632	34	3.59	-6.83	14.01	0.483	4.01	4.70	0.397
Albumin (g/L)	20	0.75	0.4	0.074	21	-0.10	0.6	0.873	20	0.55	0.5	0.308	21	0.48	0.5	0.362	41	0.27	-1.04	1.59	0.676	0.21	0.70	0.771
HbA1C (%)	20	-0.19	0.1	0.003	23	0.02	0.0	0.643	20	-0.02	0.0	0.577	23	-0.04	0.0	0.318	43	-0.15	-0.29	-0.01	0.042	-0.15	0.06	0.017
Fasting glucose (mmol/L)	20	-0.12	0.1	0.252	23	-0.04	0.1	0.583	20	-0.17	0.1	0.010	22	-0.15	0.1	0.076	42	0.03	-0.23	0.28	0.842	0.02	0.12	0.859

Total cholesterol (mmol/L)	19	-0.32	0.1	0.014	19	-0.12	0.1	0.239	20	-0.40	0.1	0.002	19	-0.40	0.1	0.001	38	0.08	-0.23	0.39	0.612	0.08	0.15	0.624
Total cholesterol:HDL ratio	19	0.11	0.1	0.369	19	0.08	0.1	0.293	20	-0.05	0.1	0.612	19	-0.04	0.1	0.705	38	0.15	-0.17	0.47	0.358	0.13	0.14	0.334
HDL-C (mmol/L)	19	-0.21	0.1	0.011	21	-0.09	0.0	0.024	20	-0.10	0.0	0.007	21	-0.13	0.0	0.009	40	-0.07	-0.24	0.10	0.399	-0.07	0.07	0.319
LDL-C (mmol/L)	19	-0.14	0.1	0.193	19	0.02	0.1	0.860	20	-0.34	0.1	0.004	19	-0.32	0.1	0.001	38	0.18	-0.07	0.44	0.154	0.18	0.13	0.183
Non-HDL-C (mmol/L)	19	-0.17	0.1	0.134	19	-0.03	0.1	0.746	20	-0.31	0.1	0.006	19	-0.28	0.1	0.006	38	0.11	-0.17	0.39	0.433	0.10	0.14	0.466
Triglycerides (mmol/L)	19	-0.07	0.1	0.441	19	-0.11	0.1	0.128	20	0.06	0.1	0.304	19	0.12	0.1	0.088	38	-0.18	-0.40	0.03	0.097	-0.19	0.10	0.057
C-reactive protein (mg/L)	20	-0.79	0.3	0.027	21	-0.26	0.4	0.567	20	-0.51	0.9	0.580	20	-0.24	0.7	0.743	40	-0.55	-2.15	1.05	0.486	-0.57	0.90	0.531
PoF Food available	15	-1.06	0.3	0.002	20	-0.18	0.2	0.349	12	-0.10	0.3	0.745	22	-0.49	0.2	0.018	37	-0.56	-1.21	0.09	0.088	-0.46	0.32	0.150
PoF Food present	15	-1.20	0.3	0.002	20	-0.25	0.2	0.165	12	0.00	0.3	1.000	22	-0.73	0.2	0.002	37	-0.47	-1.20	0.25	0.195	-0.48	0.34	0.165
PoF Food tasted	15	-0.61	0.3	0.041	20	-0.21	0.2	0.272	12	-0.13	0.3	0.627	22	-0.31	0.1	0.007	37	-0.30	-0.92	0.31	0.311	-0.27	0.28	0.337
PoF Total	15	-0.96	0.3	0.002	20	-0.21	0.2	0.228	12	-0.08	0.3	0.765	22	-0.51	0.1	0.002	37	-0.45	-0.99	0.10	0.105	-0.41	0.28	0.151
CoEQ Craving control	15	34.67	6.0	0.000	20	10.80	5.1	0.046	12	6.92	4.6	0.165	22	18.29	5.7	0.004	37	16.38	-0.78	33.53	0.061	16.63	8.07	0.044
CoEQ Craving for sweet	15	-20.81	3.9	0.000	20	-14.06	3.6	0.001	12	-6.20	3.1	0.073	22	-15.01	3.2	0.000	37	-5.80	-16.05	4.44	0.258	-2.87	4.84	0.555
CoEQ Craving for savoury	15	-20.75	5.2	0.001	20	-9.29	4.2	0.040	12	0.15	4.2	0.973	21	-9.35	3.5	0.014	37	-11.40	-23.60	0.79	0.066	-11.01	6.14	0.078
CoEQ Positive Mood	15	-0.48	4.9	0.923	20	0.08	3.1	0.981	12	6.79	4.6	0.165	22	2.36	3.5	0.507	37	-2.85	-14.78	9.08	0.631	-3.71	5.62	0.512
CoEQ control over craved nominated food	15	-37.53	8.5	0.001	20	-17.05	7.6	0.037	12	-7.92	7.3	0.299	22	-20.68	8.8	0.029	37	-16.85	-42.84	9.14	0.197	-18.32	12.26	0.141

Unadjusted change from baseline assessed using paired t-test and between diets using two-sample t-test. Estimated marginal means and 95% confidence intervals computed from mixed-effects models adjusted for randomisation arm and night-shift status, with an interaction term for diet and randomisation arm, and a random effect for participant. Abbreviations: 95%CI: 95% confidence interval; BMI: body mass index; CoEQ: Control of Eating Questionnaire; CRP: c-reactive protein; DBP: diastolic blood pressure; HbA1c: glycated haemoglobin; HDL-C: high-density lipoprotein cholesterol; HR: heart rate; LDL-C: low-density lipoprotein cholesterol; MPF: minimally processed food; Non-HDL-C: Non-high-density lipoprotein cholesterol; PFS: Power of Food Scale; PP: per-protocol; SBP: systolic blood pressure; SE: standard error; UPF: ultra-processed food.

Supplementary Table 23: Sensitivity models for missing data, and model covariate selection results

**Model covariate selection**

ITT (N = 50)	Effect estimate (MPF - UPF)	SE	p value (Diet)	p value (adjustment covariate)
**Base model**				
Diet + Randomisation arm + Diet*Randomisation arm interaction, and a random effect for participant	-1.02	0.432	0.023	Randomisation arm < 0.001; Diet*Randomisation arm < 0.001
**Base model + Randomisation stratification variables (ethnicity, sex, night-shift status)**				
Base model + Ethnicity	-1.02	0.432	0.023	0.502
Base model + Sex	-1.03	0.433	0.022	0.734
Base model + Night-shift status	-1.01	0.431	0.024	0.020
Base model + Ethnicity + Sex + Night-shift status	-1.01	0.432	0.024	Only night-shift status significant (p = 0.027)
**Base model + Unbalanced baseline participant characteristics (estimated baseline BMR)**				
Base model + Baseline estimated BMR	-1.03	0.434	0.022	0.130
Base model + Night-shift status + Baseline estimated BMR	-1.01	0.432	0.023	0.324
**Sensitivity Analysis for missing data**				
**Inverse probability weighting (stabilised weights)**	**Effect estimate (MPF - UPF)**	**SE**	**p value (Diet)**	
Propensity score estimated from: Ethnicity + Sex + Night-shift status + baseline estimated BMR	-1.01	0.431	0.023	
Propensity score estimated from: Ethnicity + Sex + Night-shift status + Baseline estimated BMR + Baseline energy intake + Baseline weight	-1.01	0.431	0.023	
Propensity score estimated from: Ethnicity + Sex + Night-shift status + Occupation + Education + Family history of obesity + Baseline estimated BMR + Baseline energy intake + Baseline weight	-1.02	0.431	0.022	
Propensity score estimated from: Ethnicity + Sex + Night-shift status + Occupation + Education + Family history of obesity + Baseline estimated BMR + Baseline energy intake + Baseline weight + Randomisation arm	-1.03	0.430	0.020	
**Multiple imputation with chained equations**	**Effect estimate (MPF - UPF)**	**SE**	**p value (Diet)**	
Percent weight change imputed using model variables: Diet + Randomisation arm + Night-shift status + Percent Weight Change	-1.02	0.442	0.026	
Percent weight change imputed using model and auxillary variables: Diet + Randomisation arm + Ethnicity + Sex + Night-shift status + Occupation + Education + Family history of obesity + Baseline estimated BMR + Baseline energy intake + Baseline weight	-1.00	0.462	0.037	
**Sensitivity Analysis of primary outcome for adherence data (N = 38)**	**Effect estimate (MPF - UPF)**	**SE**	**p value (Diet)**	

Primary outcome analysis for provided adherence data only	-1.14	0.554	0.048	

Estimated marginal means and 95% confidence intervals computed from mixed-effects models adjusted as described, with a random effect for participant. Abbreviations: BMR: basal metabolic rate; ITT: intention to treat; MPF: minimally processed food; UPF: ultra-processed food

Supplementary Table 24: Average nutrient composition of the provided MPF and UPF diets and Eatwell Guide recommendations[1–5].

per 2000 kcal of provided diet	Eatwell Guide recommendations	MPF diet	UPF diet
Energy (kcal/day)		2000	2000
Energy density (kcal/g)		1.25	1.60
UPF (kcal)	N/A	34.9	1812.6
UPF (%)	N/A	1.7%	90.7%
MPF (kcal)	N/A	1643.0	61.7
MPF (%)	N/A	82.1%	3.1%
Total fat (g)	78 g or less / 2000 kcal	76.6	70.1
Total fat (%)	35% of provided energy intake or below	34.2%	31.6%
Saturates (g)	24 g or less / 2000 kcal	21.5	19.0
Saturates (%)	10% of provided energy intake or below	9.6%	8.60%
Carbohydrate (g)	267 g / 2000 kcal	255.0	250.2
Carbohydrate (%)	Around 50% of provided energy intake	50.5%	50.0%
Total sugars (g)	Less than 18% of total energy	66.1	83.7
Total sugars (%)	Less than 90 g / 2000 kcal	13.1%	16.7%
Protein (g)	45 g / 2000 kcal	77.2	78.2
Protein (%)	Around 15% of provided energy intake[5*]	15.3%	15.6%
Salt (g)	Less than 6 g per day. Intake in line with current UK average intakes[6], aiming for below 6 g per day	3.0	3.8
Fibre (g)	At least 30 g / 2000 kcal	35.9	32.4
Fruit and Veg (portions/day)	Five portions per day	6.1	5.1

Abbreviations: MPF: minimally processed food; UPF: ultra-processed food.

1. Public Health England. Government recommendations for energy and nutrients for males and females aged 1–18 years and 19+ years. London, UK: Public Health England, 2016.

2. NHS. The Eatwell Guide. NHS. https://www.nhs.uk/live-well/eat-well/the-eatwell-guide/ (accessed Oct 11, 2021).

3. Scientific Advisory, Committee on Nutrition. Salt and Health. 2003. https://assets.publishing.service.gov.uk/media/5a74983de5274a44083b7ef6/SACN_Salt_and_Health_report.pdf (accessed Oct 3, 2024).

4. Scientific Advisory Committee on Nutrition. Update on trans fatty acids and health: Position statement by the Scientific Advisory Committee on Nutrition. 2007. https://assets.publishing.service.gov.uk/media/5a7eda08e5274a2e87db24f4/SACN_Update_on_Trans_Fatty_Acids_2007.pdf (accessed Oct 2, 2024).

5. Scheelbeek P, Green R, Papier K, et al. Health impacts and environmental footprints of diets that meet the Eatwell Guide recommendations: analyses of multiple UK studies. BMJ Open 2020; 10: e037554.

6. Public Health England. National Diet and Nutrition Survey: Assessment of salt intake from urinary sodium in adults (aged 19 to 64 years) in England, 2018 to 2019. GOV.UK. https://www.gov.uk/government/statistics/national-diet-and-nutrition-survey-assessment-of-salt-intake-from-urinary-sodium-in-adults-aged-19-to-64-years-in-england-2018-to-2019 (accessed Oct 3, 2024).

*Protein percentage estimates are from published papers reporting adherence to Eatwell Guide dietary guidelines[5], as a proportion of total energy intake, consumption of ~15% protein is necessary to achieve the recommended balance of <35% fat and ~50% carbohydrate.

Supplementary Table 25: Menus for the provided MPF and UPF diets.

	MPF Menu	UPF Menu
	**Day 1**	
Breakfast	Cinnamon and apple overnight oats	Oat and fruit bars
Lunch	Mexican chicken with flatbread	Chicken wraps
Dinner	Cottage pie with green beans and corn	Cottage pie, greens and corn
Snacks	Fruit and nut bar, Fruit, Vanilla and berry yoghurt pot	Nut snacks, Yoghurts
	**Day 2**	
Breakfast	Summer Fruits overnight oats, Fruit	Multigrain cereal with plant-based milk, Fruit bars
Lunch	Chicken salad with flatbread, Fruit and nut bar	Chicken sandwich, Fruit and nut bar
Dinner	Shredded hoisin duck and stir-fried egg noodles	Gyoza with egg noodles
Snacks	Vanilla and berry yoghurt pot, Raspberry yoghurt pot	Fruit and custard, Ice cream
	**Day 3**	
Breakfast	Raspberry oat pot	Granola with plant-based milk, Fruit bars
Lunch	Sticky BBQ ribs with smoky BBQ rice, Fruit	Ribs and rice, Fruit snack
Dinner	Salmon with herbed new potatoes and mixed vegetables	Salmon, potatoes and corn
Snacks	Dark chocolate and strawberry parfait, Fruit and nut bar, Fruit	Chocolate and dairy desserts, Flapjack
	**Day 4**	
Breakfast	Peanut and banana overnight oats, Fruit	Cereal with plant-based milk, Fruit bars
Lunch	Chicken salad, Fruit	Sandwich, Fruit and nut bar
Dinner	Cheese and broccoli pasta with greens	Cheese and pasta with peas and sweetcorn
Snacks	Banana bread oat muffin, Yoghurt pot, Fruit	Protein bar, Flapjack, Oats and yoghurt
	**Day 5**	
Breakfast	Strawberry overnight oats, Fruit	Cereal with plant-based milk, Drinking yoghurt
Lunch	Chicken tikka & onion bhaji with flatbread, Fruit	Chicken sandwich, Fruit bars
Dinner	Spaghetti Bolognese with greens	Lasagne, vegetables and salad
Snacks	Blueberry, oat, nut & seed muffin, Mediterranean vegetable pasta salad	Fruit and nut bars, Meal-replacement drink

	**Day 6**	
Breakfast	Cherry oat pot, Fruit	Granola with plant-based milk, Fruit bars
Lunch	BBQ beef noodles	BBQ Beef Noodles, Reduced salt crisps
Dinner	Chicken and vegetable Thai curry with jasmine rice	Tikka masala curry, vegetables and salad
Snacks	Dark chocolate and nut snack pot, Flaked almond and cinnamon rice pudding	Protein bars, Rice cakes, Nuts
	**Day 7**	
Breakfast	Blueberry oat pot, Fruit	Cereal with plant-based milk
Lunch	Pesto, Bean and med veg with flatbread, Nut bar	Caesar salad and ham, Fruit snacks
Dinner	Chicken stir fry, rice and vegetables	Sweet and sour chicken, rice and vegetables
Snacks	Mustard dressed potato salad, Strawberry, yoghurt and toasted oat pot	Meal-replacement drink, Plant-based yoghurt, Oat bar

Abbreviations: MPF: minimally processed food; UPF: ultra-processed food.

Supplementary Table 26: Pictures of meals and snacks of the provided MPF and UPF diets.

MPF diet<br><br>*Participants provided with multiple fruits on the diet. Apples and bananas pictured for menus as example fruits.	UPF diet<br><br>*Participants provided with squash and artificially-sweetened beverages to consume at their discretion.
Breakfast Cinnamon and apple overnight oats<br>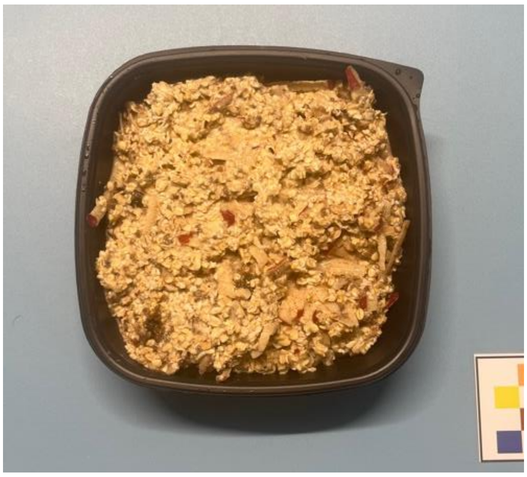	Breakfast Oat and fruit bars<br>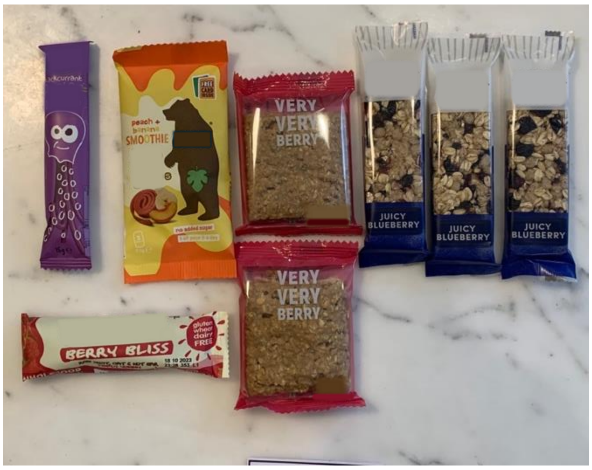
Lunch Mexican chicken with flatbread<br>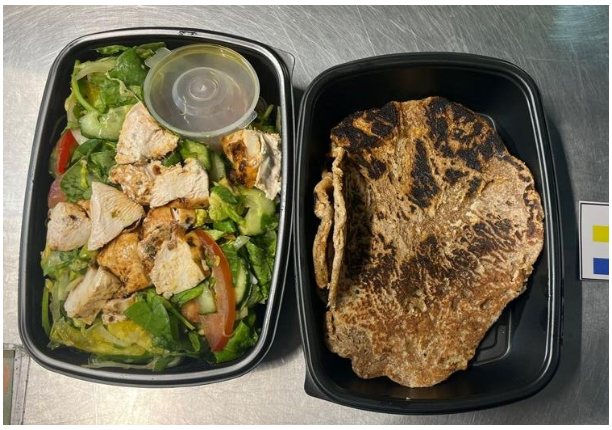	Lunch Chicken Wraps<br>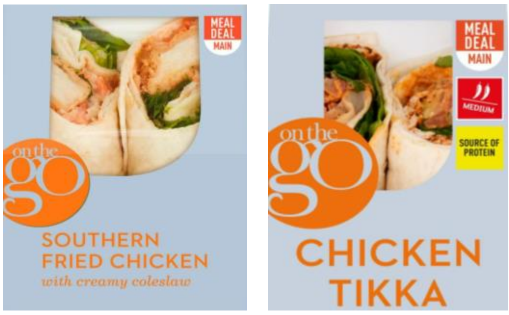
Dinner Cottage pie with green beans and corn<br>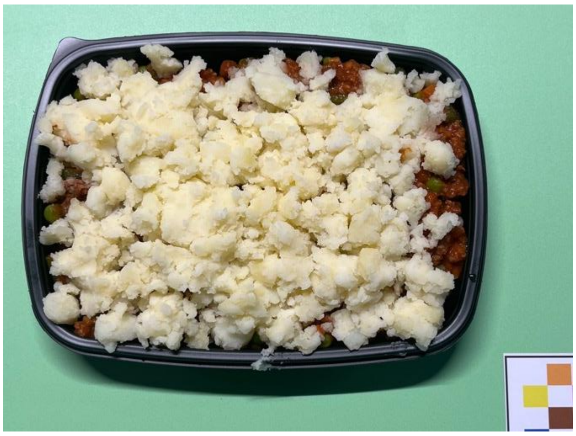	Dinner Cottage pie, greens and corn<br>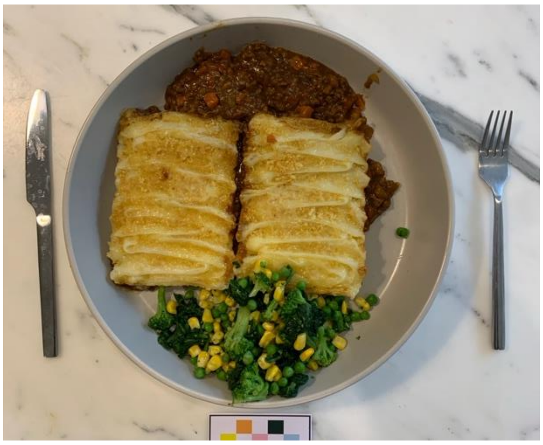
Snacks Fruit and nut bar, Fruit, Vanilla and berry yoghurt pot	Snacks Nut snacks, Yoghurts

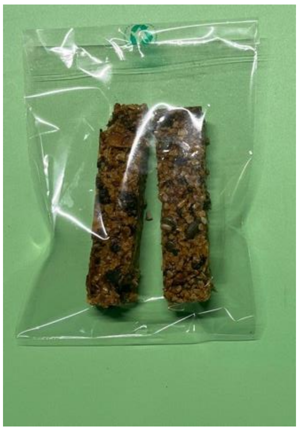 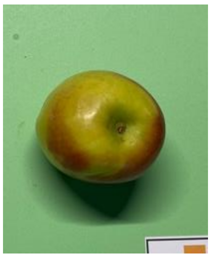 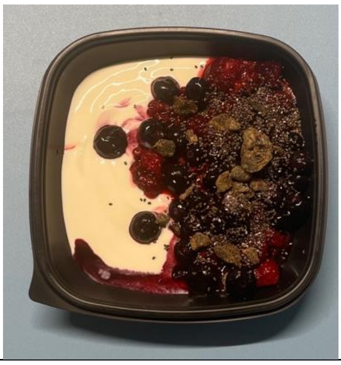	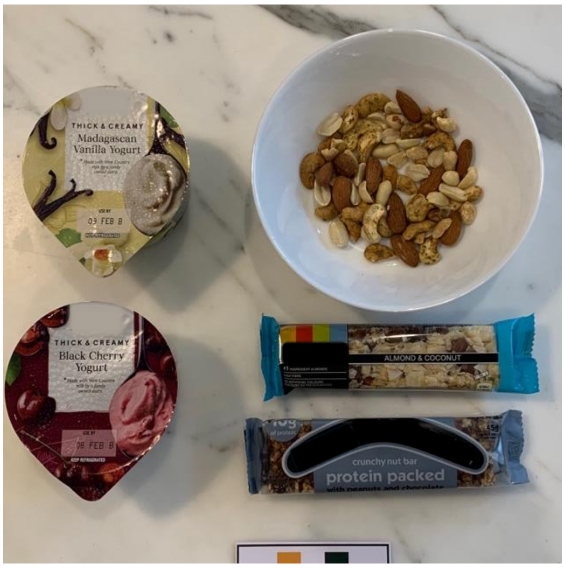
Breakfast Summer fruit overnight oats, Fruit<br>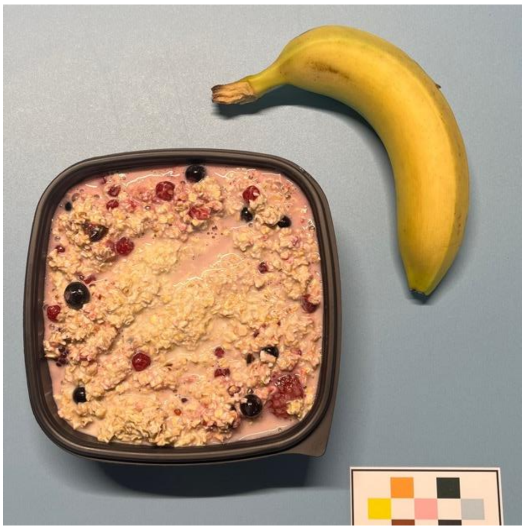	Breakfast Multigrain cereal with plant-based milk, Fruit bars<br>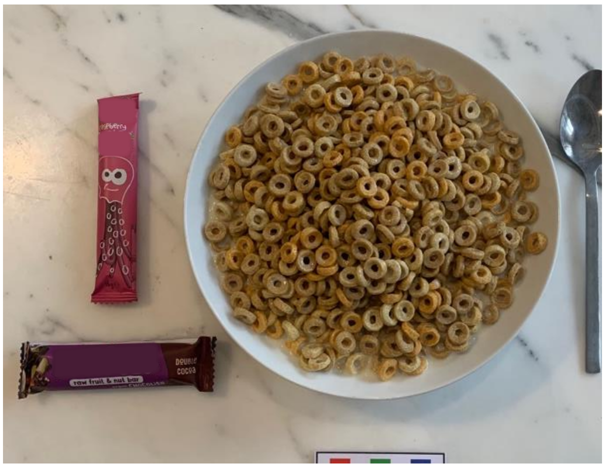
Lunch Chicken salad with flatbread, Fruit and nut bar<br>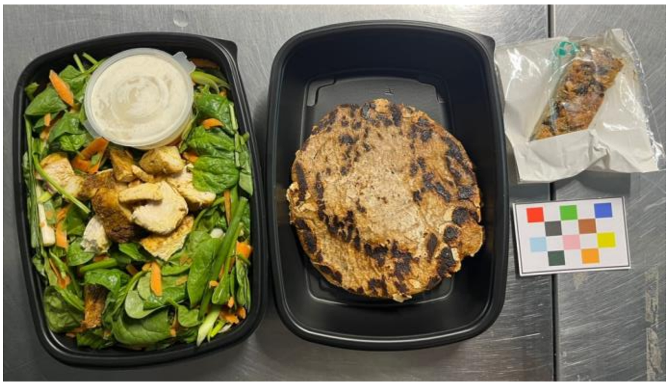	Lunch Chicken sandwich, Fruit and nut bar<br>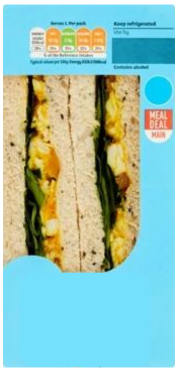 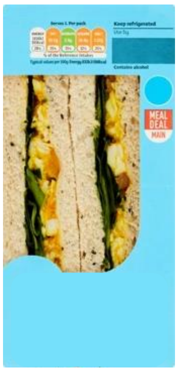 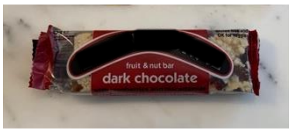

<table>
<tr>
<td>Dinner Shredded hoisin duck and stir-fried egg noodles
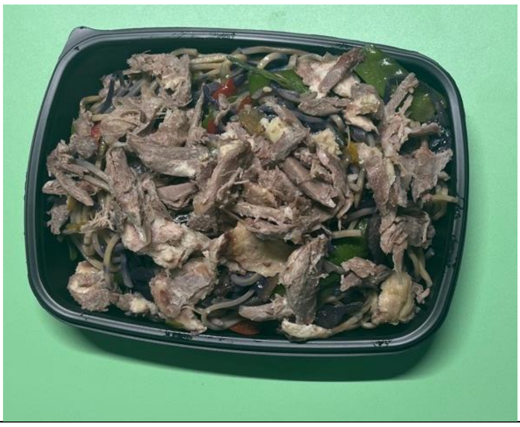</td>
<td>Dinner Gyoza with egg noodles
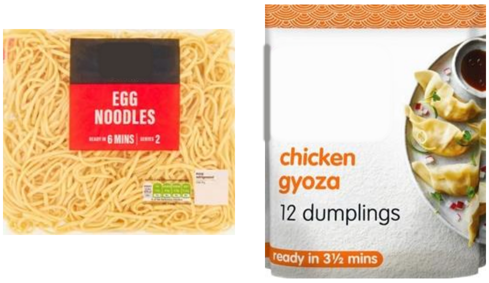
</td>
</tr>
<tr>
<td>Snacks Vanilla and berry yoghurt pot, Raspberry yoghurt pot
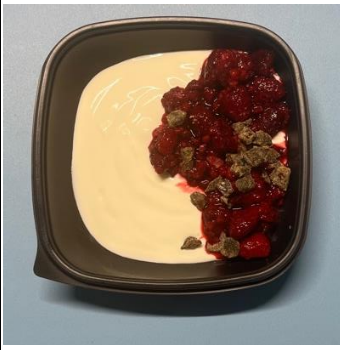 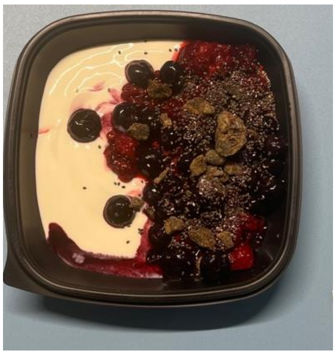</td>
<td>Snacks Fruit and custard, Ice cream
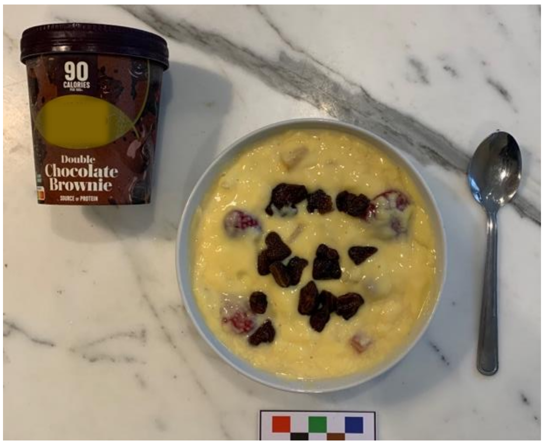
</td>
</tr>
<tr>
<td>Breakfast Raspberry oat pot
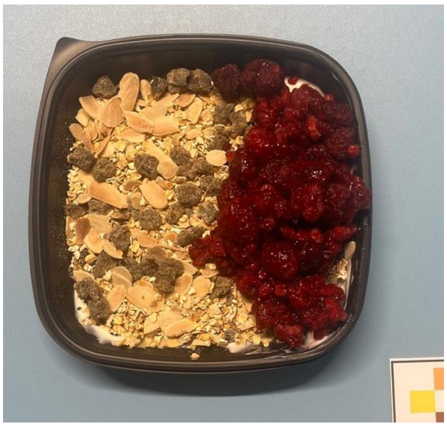</td>
<td>Breakfast Granola with plant-based milk, Fruit bars
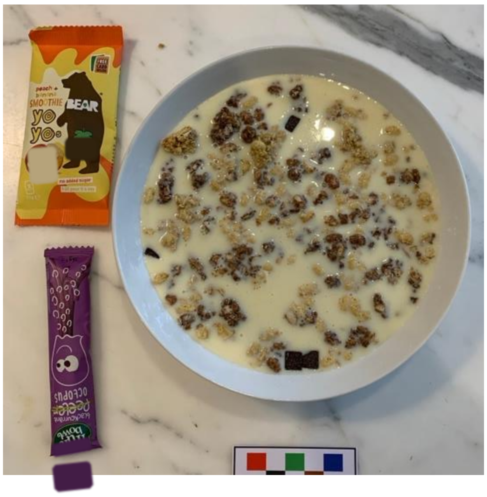
</td>
</tr>
</table>

Lunch Sticky BBQ ribs with smoky BBQ rice, Fruit

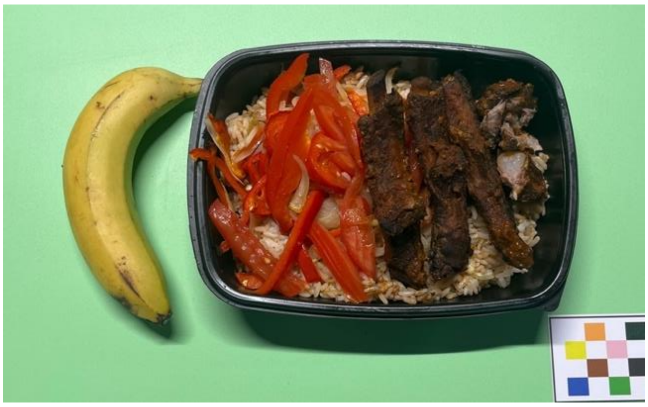

Lunch Ribs and rice, Fruit snack

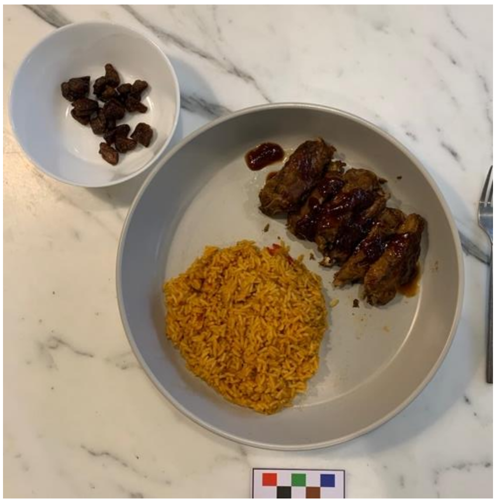

Dinner Salmon with herbed new potatoes and mixed vegetables

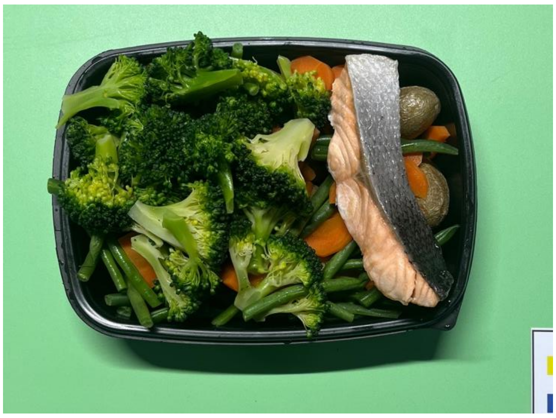

Dinner Salmon, potatoes and corn

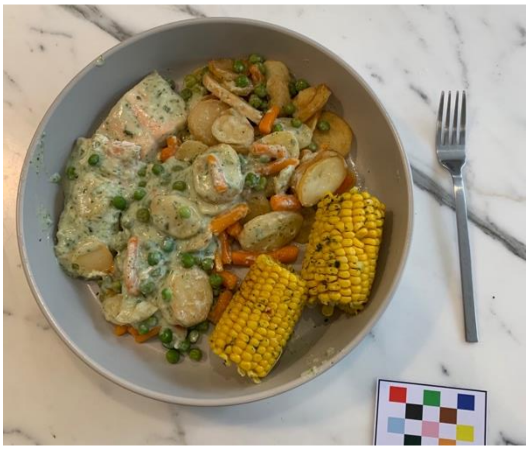

Snacks Dark chocolate and strawberry parfait, Fruit and nut bar, Fruit

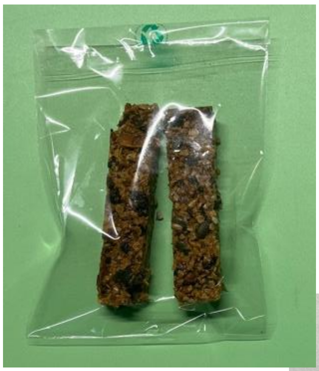

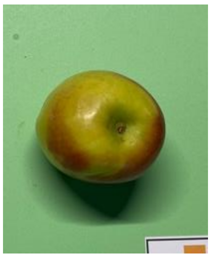

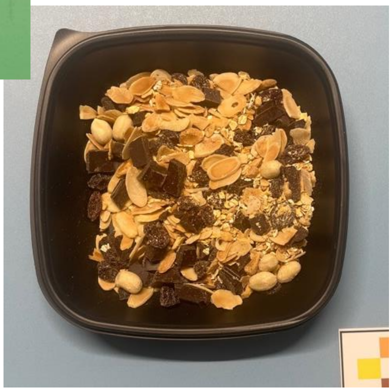

Snacks Chocolate and dairy desserts, Flapjack

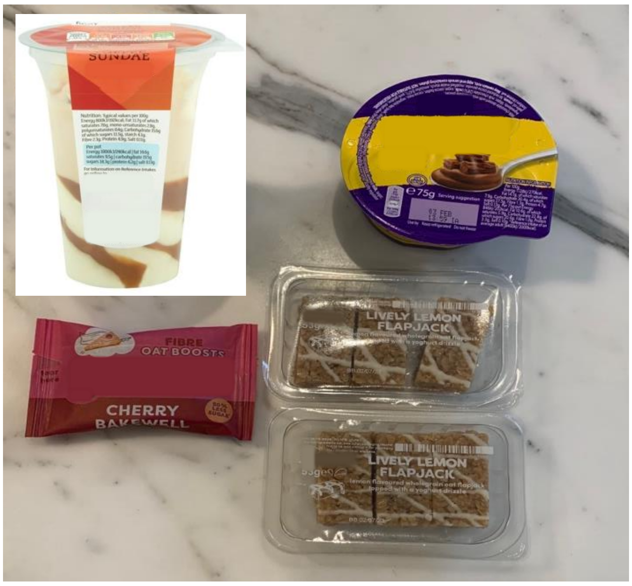

Breakfast Peanut and banana overnight oats, Fruit

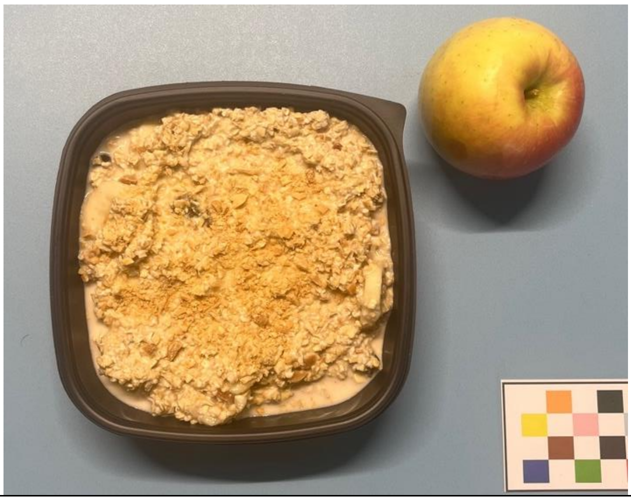

Breakfast Cereal with plant-based milk, Fruit bars

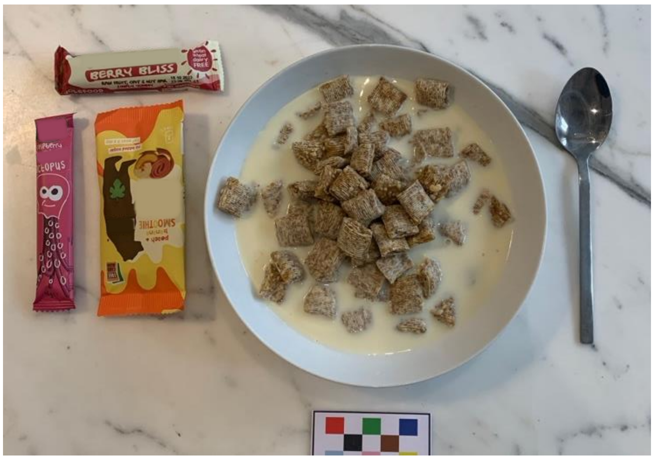

Lunch Chicken salad, Fruit

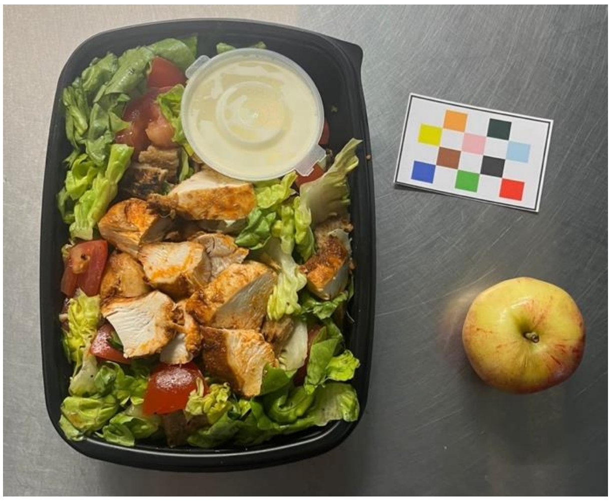

Lunch Sandwich, Fruit and nut bar

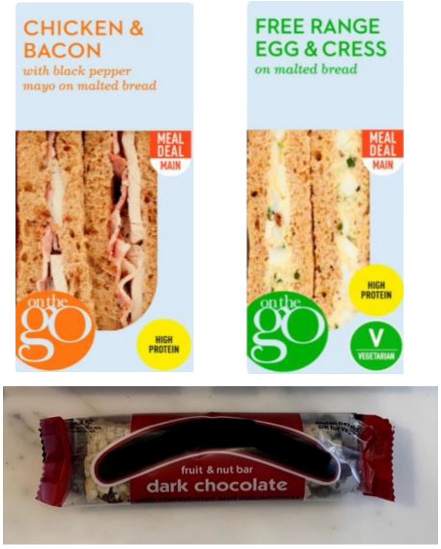

Dinner Cheese and broccoli pasta with greens

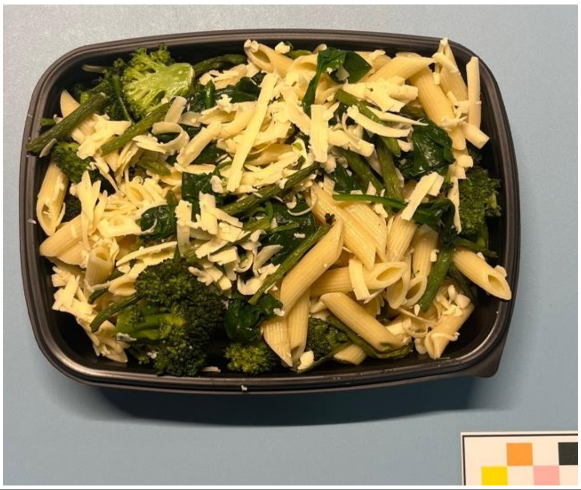

Dinner Cheese and pasta with peas and sweetcorn

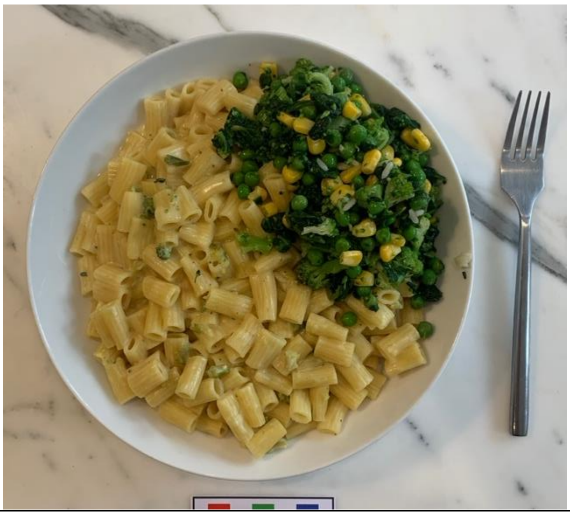

Snacks Banana bread oat muffin, Yoghurt pot, Fruit 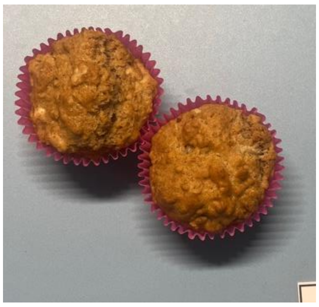 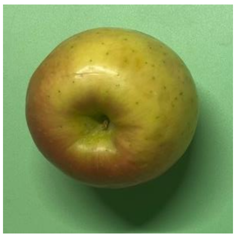 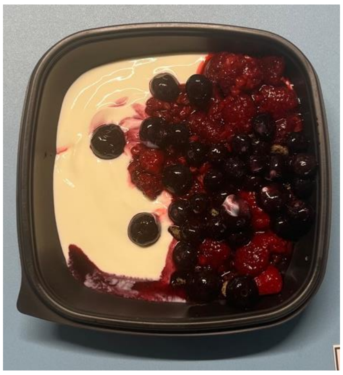	Snacks Protein bar, Flapjack, Oats and yoghurt 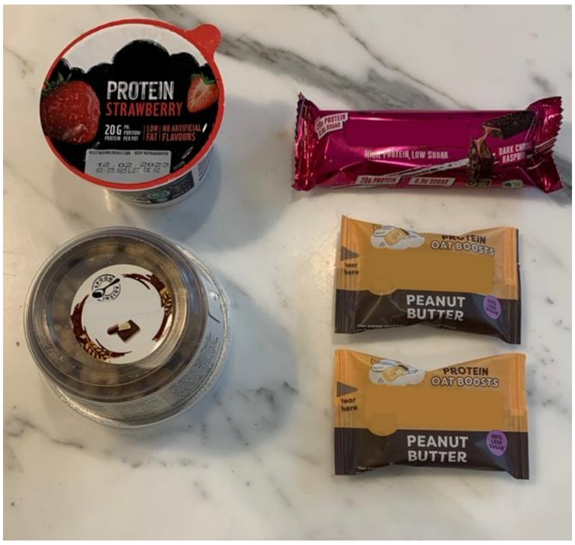
Breakfast Strawberry overnight oats, Fruit 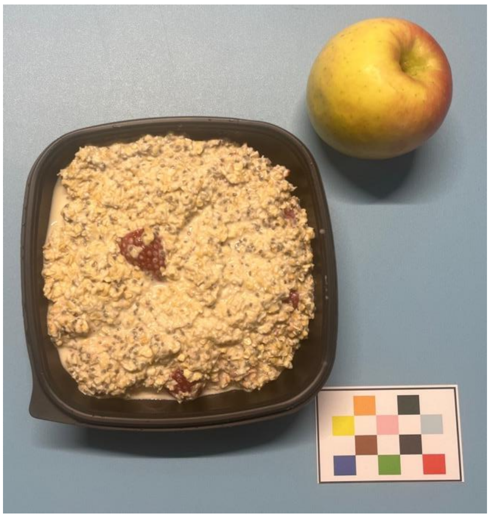	Breakfast Cereal with plant-based milk, Drinking yoghurt 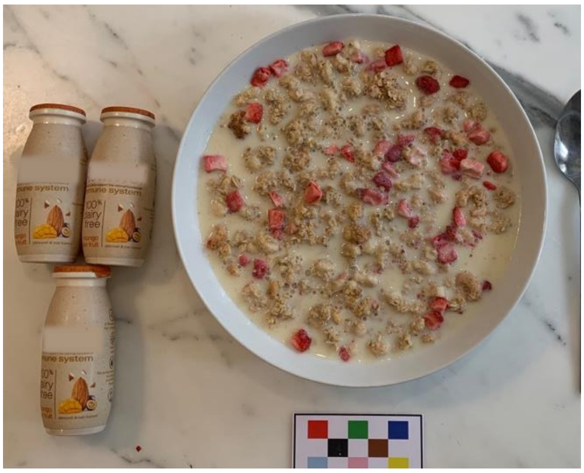
Lunch Chicken tikka and onion bhaji with flatbread, Fruit 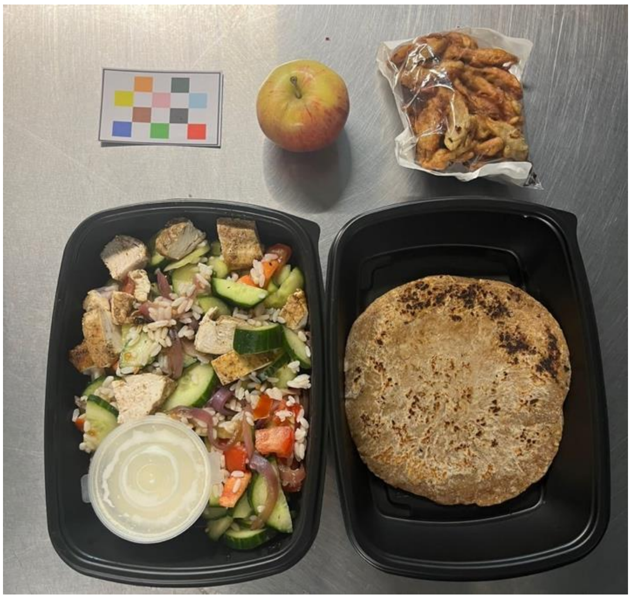	Lunch Chicken sandwich, Fruit bars 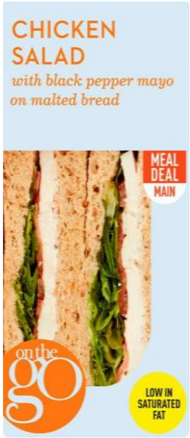 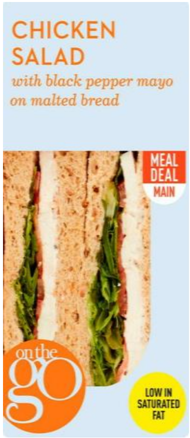 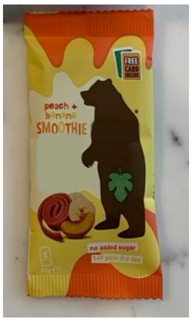 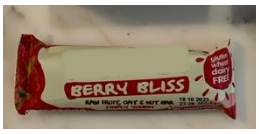

Dinner Spaghetti Bolognese with greens 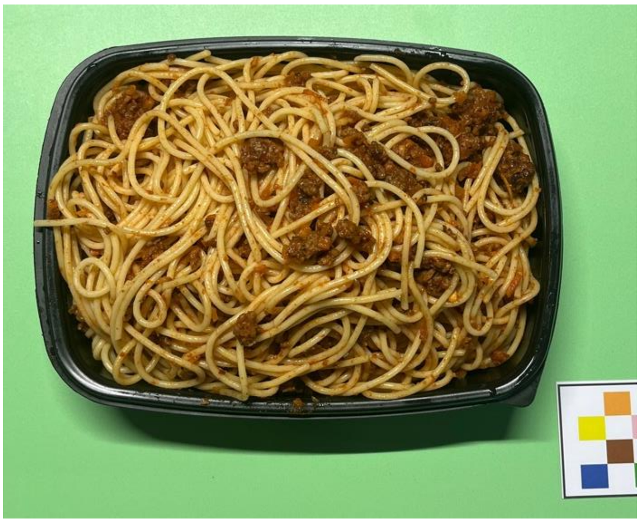	Dinner Lasagne, vegetables and salad 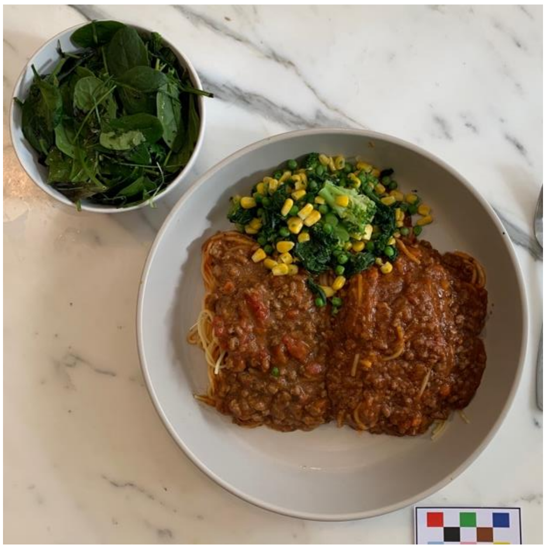
Snacks Blueberry, oat, nut & seed muffin, Mediterranean vegetable pasta salad 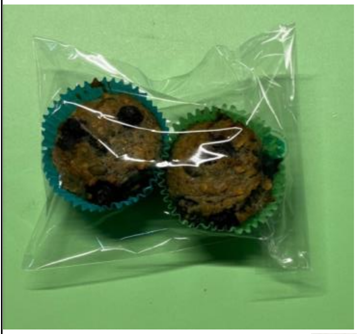 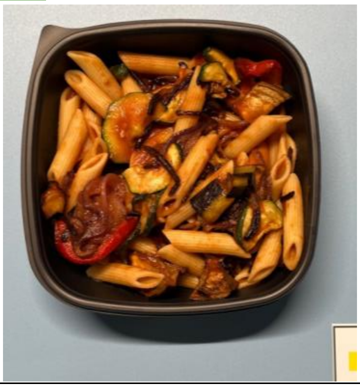	Snacks Fruit and nut bars, Meal-replacement drink 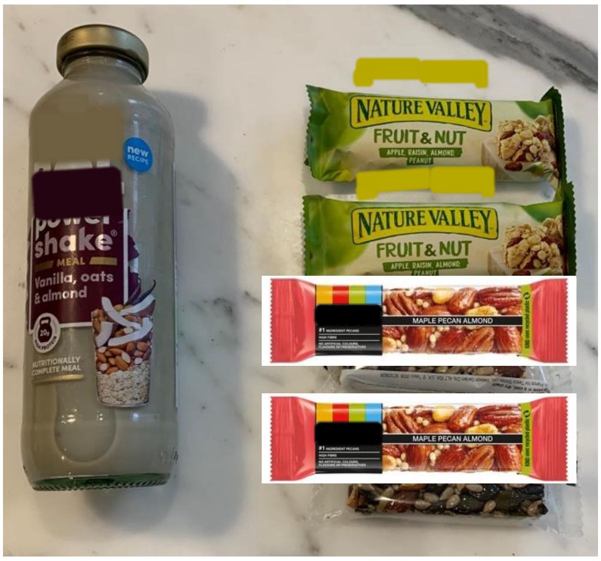

Breakfast Cherry oat pot, Fruit<br>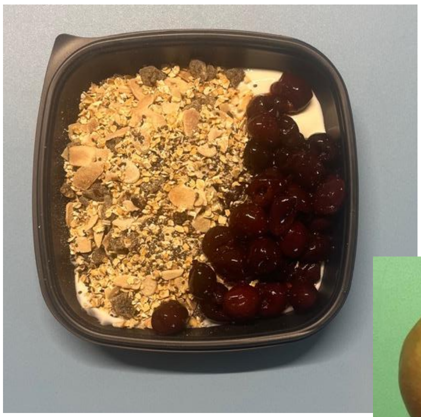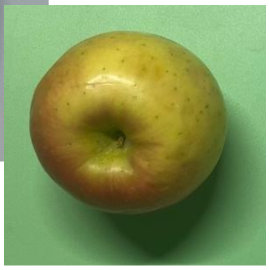	Breakfast Granola with plant-based milk, Fruit bars<br>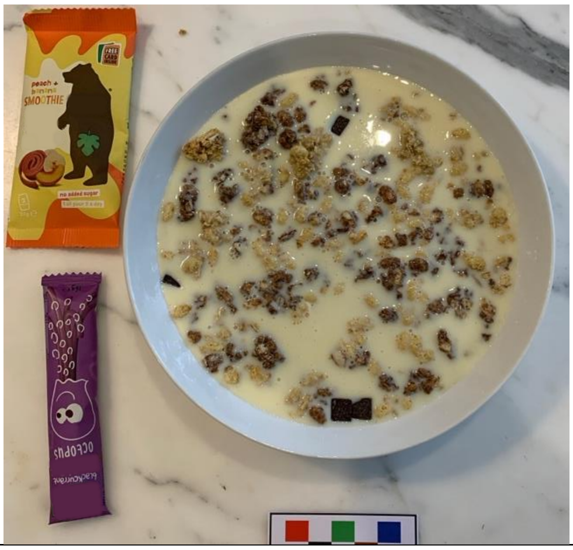
Lunch BBQ beef noodles<br>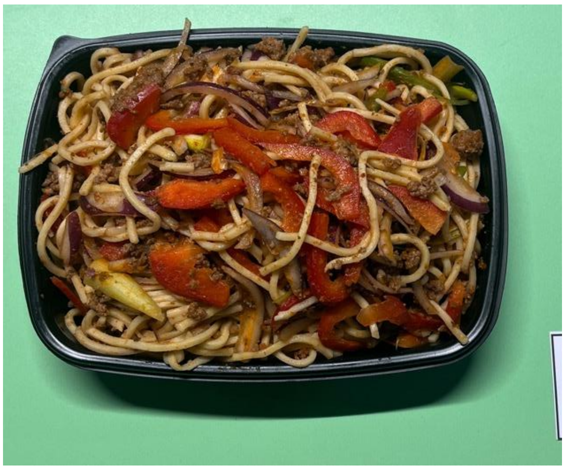	Lunch BBQ Beef Noodles, Reduced salt crisps<br>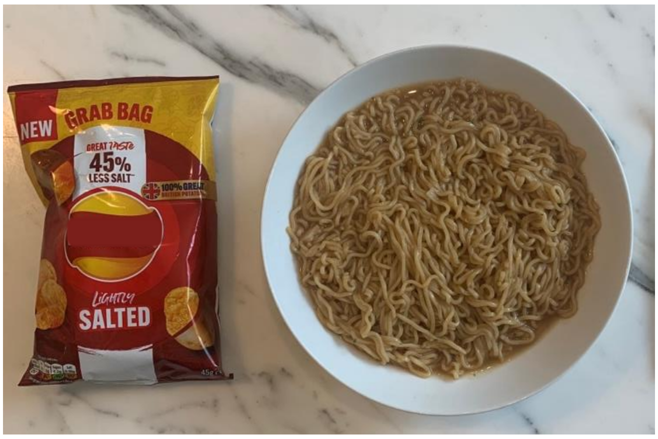
Dinner Chicken and veg Thai curry with jasmine rice<br>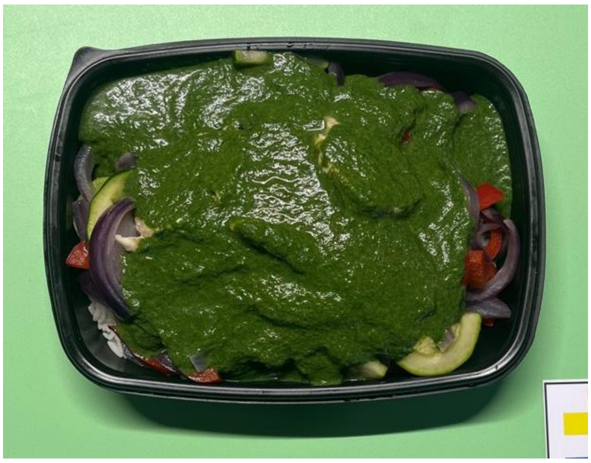	Dinner Tikka masala curry, vegetables and salad<br>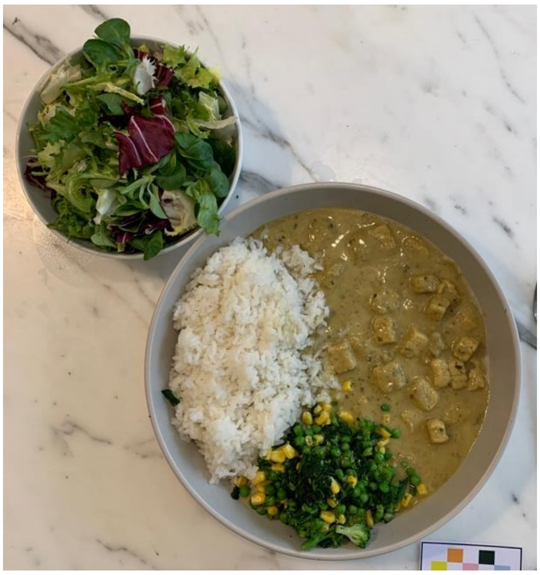
Snacks Dark chocolate and nut snack pot, Flaked almond and cinnamon rice pudding	Snacks Protein bars, Rice cakes, Nuts

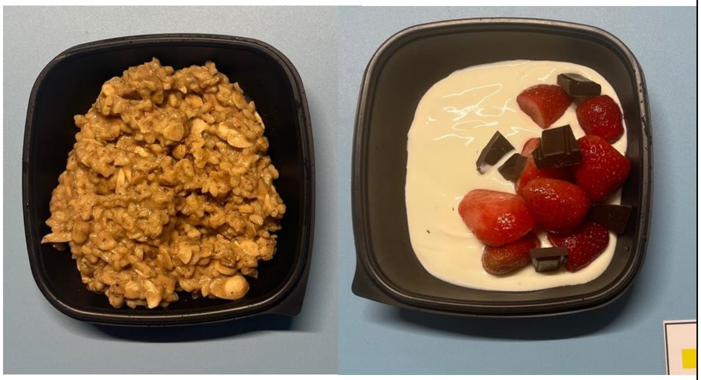	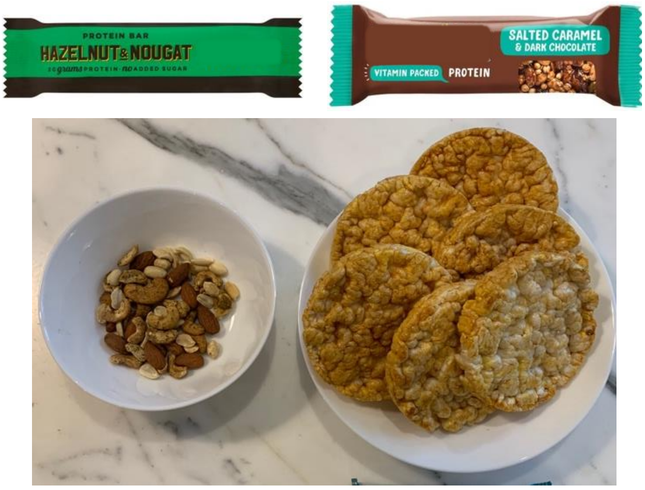
Breakfast Blueberry oat pot, Fruit<br>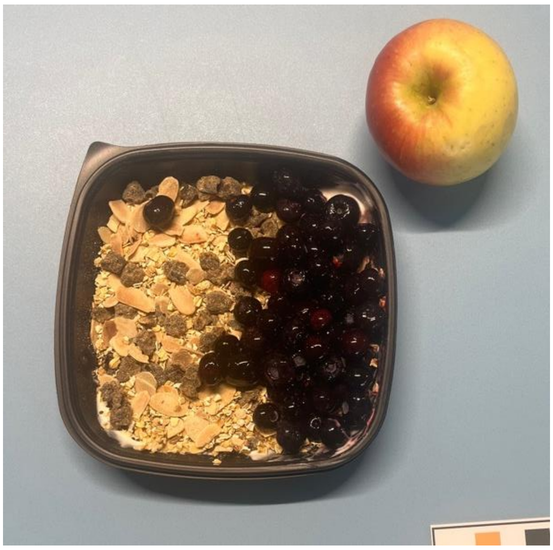	Breakfast Cereal with plant-based milk<br>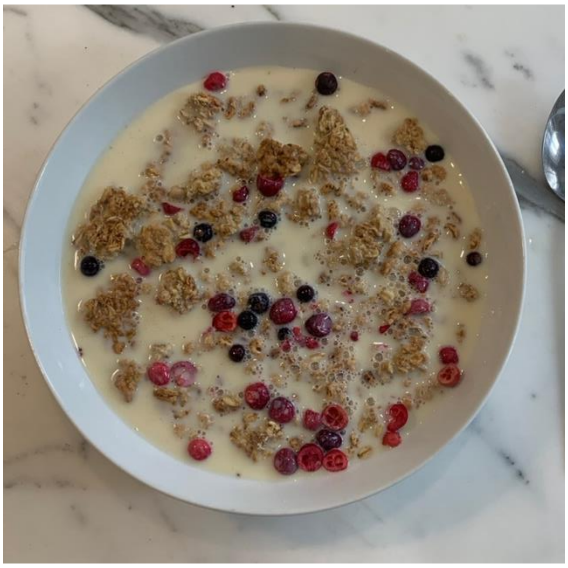
Lunch Pesto, Bean and Mediterranean vegetables with flatbread, Fruit and nut bar<br>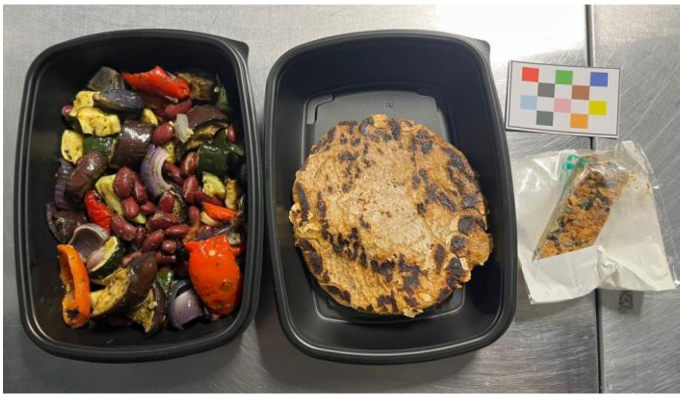	Lunch Caesar salad and ham, Fruit snacks<br>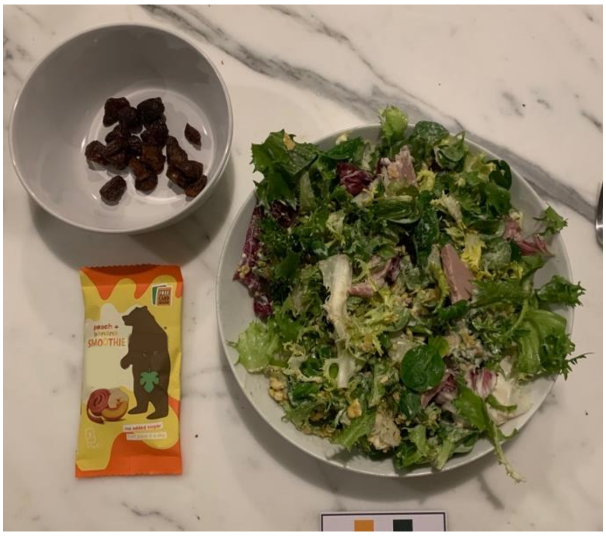

<table>
<tr>
<td>Dinner Chicken stir fry, rice and vegetables
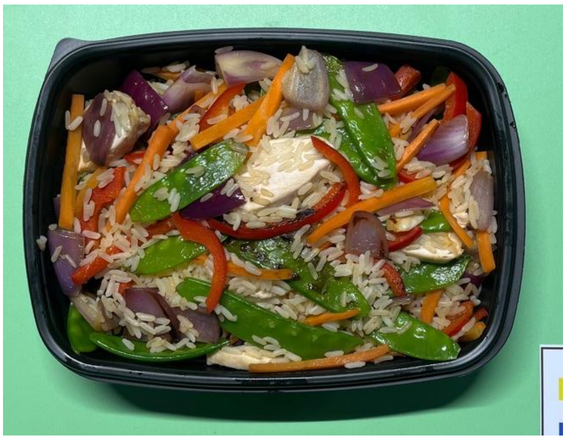</td>
<td>Dinner Sweet and sour chicken, rice and vegetables
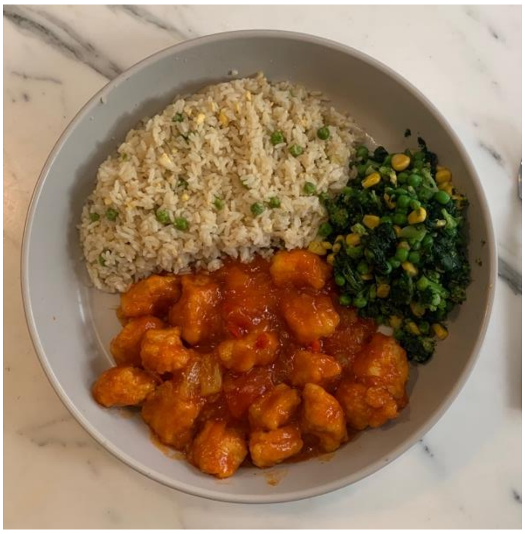</td>
</tr>
<tr>
<td>Snacks Mustard dressed potato salad, Strawberry, yoghurt and toasted oat pot
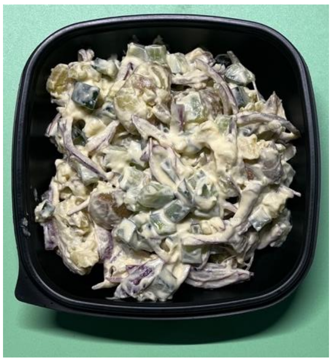 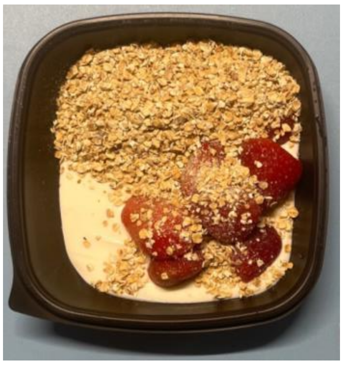</td>
<td>Snacks Meal-replacement drink, Plant-based yoghurt, Oat bar
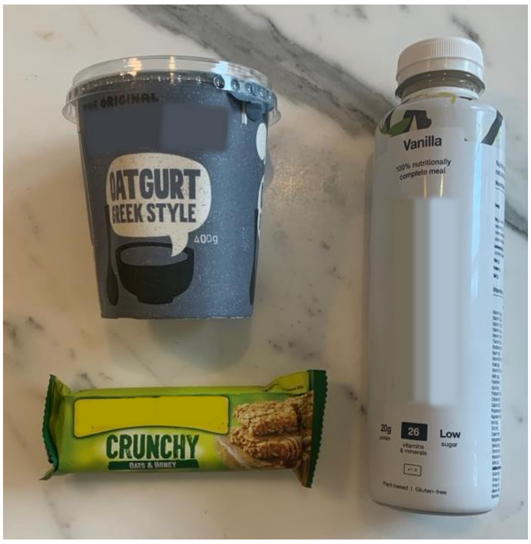</td>
</tr>
</table>

Abbreviations: MPF: minimally processed food; UPF: ultra-processed food.

Supplementary Table 27: Descriptors of post-hoc diet ratings.

Questions were asked about the diets at the end of the trial. Each question was rated on a 0-10 Likert scale, where 0 indicates a negative, poor, or bland experience, or the least intensity of the attribute being evaluated, and 10 indicates a positive, excellent, or flavourful experience, or the greatest intensity of the attribute being evaluated. Questions were asked for both the ultra-processed diet and minimally processed diets.

1. Diet overall	A rating of the overarching experience of the 8 weeks of the diet.
2. Meals and snacks	An overall rating of the food/drinks/snacks provided on the diet.
3. Flavours and taste	A rating of the flavours and taste of the foods/snacks in the diet.
4. Textures	A rating of the textures of the foods/snacks in the diet.
5. Portion sizes	A rating of the amount of food provided on the diet.
6. Delivery and preparation	A rating of the ease of delivery and required preparation of the diet.
7. Hunger level	A rating of how hungry participants felt whilst on the diet (0 being extremely hungry and 10 being extremely full).
8. Contentment level	A rating of how happy/content participants felt whilst on the diet.
9. Diet sustainability	A rating of the likelihood that participants could adopt the diet in the future (with 0 being very unlikely and 10 being very likely).
