# Supplementary material for: Ultraprocessed or minimally processed diets following healthy dietary guidelines on weight and cardiometabolic health: a randomized, crossover trial
Source: Nat Med. 2025 Aug 4;31(10):3297–308. doi: 10.1038/s41591-025-03842-0 (PMC12532614; doi:10.1038/s41591-025-03842-0)
Supplement: Supplementary file 2 — Reporting Summary [file 41591_2025_3842_MOESM2_ESM.pdf]

nature portfolio

Corresponding author(s): Dr Samuel Dicken

Last updated by author(s): Jun 6, 2025

Reporting Summary

Nature Portfolio wishes to improve the reproducibility of the work that we publish. This form provides structure for consistency and transparency in reporting. For further information on Nature Portfolio policies, see our Editorial Policies and the Editorial Policy Checklist.

Statistics

For all statistical analyses, confirm that the following items are present in the figure legend, table legend, main text, or Methods section.

n/a	Confirmed	
☐	☒	The exact sample size (*n*) for each experimental group/condition, given as a discrete number and unit of measurement
☐	☒	A statement on whether measurements were taken from distinct samples or whether the same sample was measured repeatedly
☐	☒	The statistical test(s) used AND whether they are one- or two-sided<br>*Only common tests should be described solely by name; describe more complex techniques in the Methods section.*
☐	☒	A description of all covariates tested
☐	☒	A description of any assumptions or corrections, such as tests of normality and adjustment for multiple comparisons
☐	☒	A full description of the statistical parameters including central tendency (e.g. means) or other basic estimates (e.g. regression coefficient) AND variation (e.g. standard deviation) or associated estimates of uncertainty (e.g. confidence intervals)
☐	☒	For null hypothesis testing, the test statistic (e.g. F, t, r) with confidence intervals, effect sizes, degrees of freedom and P value noted<br>*Give P values as exact values whenever suitable.*
☒	☐	For Bayesian analysis, information on the choice of priors and Markov chain Monte Carlo settings
☒	☐	For hierarchical and complex designs, identification of the appropriate level for tests and full reporting of outcomes
☐	☒	Estimates of effect sizes (e.g. Cohen's *d*, Pearson's *r*), indicating how they were calculated

Our web collection on statistics for biologists contains articles on many of the points above.

Software and code

Policy information about availability of computer code

Data collection	Data for this trial was first stored on paper case report forms (CRF) and then recorded on a purpose-built electronic trial database (eCRF) using REDCap (https://www.project-redcap.org). Questionnaires were provided electronically and stored directly in REDCap. The REDCap database contains range validity checks with warnings for erroneous or missing values, and initial data entry from the paper CRF was verified by a second team member for quality assurance. The REDCap application also provides a comprehensive audit trail showing all changes to the data.
Data analysis	Analyses were conducted in R version 2024.04.1+748 using standard freely-available packages. Data was presented in tables using Microsoft Excel Version 16.91 (24111020) and Figures presented using Prism 10 Version 10.2.3. Physical activity data were analysed using ActiGraph ActiLife software (v. 6.13.6). Code for the analysis is publicly available and available without restriction at: https://github.com/SamuelJDicken/UPDATE.

For manuscripts utilizing custom algorithms or software that are central to the research but not yet described in published literature, software must be made available to editors and reviewers. We strongly encourage code deposition in a community repository (e.g. GitHub). See the Nature Portfolio guidelines for submitting code & software for further information.

Data

Policy information about availability of data

All manuscripts must include a data availability statement. This statement should provide the following information, where applicable:

- Accession codes, unique identifiers, or web links for publicly available datasets
- A description of any restrictions on data availability
- For clinical datasets or third party data, please ensure that the statement adheres to our policy

As the data in this study involves clinical data from humans, the dataset is under controlled access according to General Data Protection Regulation requirements and participant written informed consent. UCL is the data controller for the data in this study. Data access requests should first contact the corresponding author (SD: samuel.dicken.20@ucl.ac.uk) to discuss data of interest and to obtain approval. Data for all outcomes in this paper can be requested. Data will be anonymised and provided in summary format (not individual-level data) before sharing to meet UK General Data Protection Regulation requirements. The timeline between requesting data and approval of data requests is three months. Data will be provided within three months of approval.

Research involving human participants, their data, or biological material

Policy information about studies with human participants or human data. See also policy information about sex, gender (identity/presentation), and sexual orientation and race, ethnicity and racism.

Reporting on sex and gender	Sex was assessed at screening (as Male or Female) and checked in medical records. Sex was required as part of the randomisation strata and used to assess generalisability of results. 50 participants (90.9%) were female. Sex by randomisation arm is reported in Table 1.
Reporting on race, ethnicity, or other socially relevant groupings	Participants were asked what their ethnicity was at screening, and they were provided a list of options to select from: White (English / Welsh / Scottish / Northern Irish, British, Irish, Romani or Irish Traveller, Any other White background); Mixed / Multiple ethnic groups (White & Black Caribbean, White & Black African, White & Asian, Any other Mixed / Multiple ethnic background); Asian / Asian British (Indian, Pakistani, Bangladeshi, Chinese, Any other Asian background); Black / African / Caribbean / Black British (African, Caribbean, Any other Black / African / Caribbean background); Other ethnic groups (Arab, Any other ethnic group (please specify)). Ethnicity was required as part of the randomisation strata and used to assess generalisability of results.
Population characteristics	Mean age was 43.2 years (standard deviation (SD): 11.4), 36 (65.5%) were of white ethnicity, 50 (90.9%) were female, and nine (16.4%) were night-shift workers. Mean weight was 89.4 kg (SD: 12.9), and body mass index (BMI) 32.7 kg/m2 (SD: 3.9). Mean habitual UPF intake was 67.4% kcal/day (SD: 8.0), with mean macronutrient and food group intakes not adherent to EWG recommendations, except for red meat intake.
Recruitment	Potential participants were identified through advertising at University College London and University College London Hospital (eg, websites, Trust email, posters, internal communications) and on social media (Twitter).<br>Interested individuals received a participant information sheet (PIS) and were offered a phone call with the research team. Researchers explained the screening procedure and the aims, methods, anticipated benefits and potential hazards of the trial, and then invited interested individuals to attend an in-person screening visit Written informed consent was obtained a minimum of 24 hours after individuals received the PIS, and was collected at the in-person screening visit. Participants could withdraw at any time without giving a reason.<br><br>Potential selection biases include that individuals with dietary restrictions (e.g., vegan, halal, kosher) were excluded due to financial and logistical constraints, limiting generalisability. However, participants with minor dietary restrictions/intolerances were eligible if such foods were not on menus. Participants were required to have a habitual UPF intake of at least 50% of daily calories to be eligible, therefore thee results also may not generalise to individuals with low-UPF intakes.
Ethics oversight	The Yorkshire & The Humber - Sheffield Research Ethics Committee approved the trial on 22nd December 2022 (22/YH/0281). The study was prospectively registered on ClinicalTrials.gov (NCT05627570).

Note that full information on the approval of the study protocol must also be provided in the manuscript.

Field-specific reporting

Please select the one below that is the best fit for your research. If you are not sure, read the appropriate sections before making your selection.

☒ Life sciences ☐ Behavioural & social sciences ☐ Ecological, evolutionary & environmental sciences

For a reference copy of the document with all sections, see nature.com/documents/nr-reporting-summary-flat.pdf

Life sciences study design

All studies must disclose on these points even when the disclosure is negative.

Sample size	The sample size is based on estimated within-participant variation. Briefly, the expected weight loss trajectory over 8 weeks was modelled using the National Institutes of Health (NIH) bodyweight planner (https://www.niddk.nih.gov/bwp) and based on data from Hall et al. showing 0.9 kg weight loss following a 2-week MPF diet, with a standard deviation of the mean difference in weight change between MPF and UPF

	diets of 1.98 kg (mean: 1.85 kg). In total, 44 participants were required to detect a mean difference of 2.7% weight change between groups, assuming weight loss on the MPF diet and no weight change on the UPF diet, with a standard deviation of the mean difference of 5.4% (power = 0.9, alpha = 0.05, two-sided paired t-test, SPSS version 27.0). The final sample size was 55, factoring for a 20% dropout rate.
Data exclusions	All data on participants providing complete primary outcome data for at least one diet of the intervention were included in the study. All data was analysed as randomised. The main intention-to-treat (ITT) analysis included participants with primary outcome values for at least one diet.
Replication	As a clinical trial, the results were not replicated in another sample. Sensitivity analyses were conducted to check robustness of results. Unadjusted analyses of primary and secondary outcomes at 8 weeks were compared with baseline, and differences in changes from baseline to 8 weeks between diets were assessed using paired t-tests. Analyses were repeated for changes in outcomes at week 4 from baseline between diets, for changes at week 4 and week 8 from baseline between diets using repeated-measures mixed-effects models, for the PP sample, and for results using data from the first period of each randomisation arm only. No interim analysis was planned or conducted.
Randomization	Participants were block randomised by the research team using Sealed Envelope (https://www.sealedenvelope.com) to either: (1) the MPF diet then UPF diet (n = 28), or (2) the UPF diet then MPF diet (n = 27). Sealed Envelope generated the random allocation sequence. Randomisation was stratified by night-shift status, sex and ethnicity.
Blinding	Researchers were not blind to assignment and enrolled participants. An independent statistician verified the primary outcome analysis whilst blind to allocation assignment. Participants were not informed of the processing groups of the diets. All participant communications omitted the terms MPF or UPF, with diets being referred to as Diet A or Diet B.

Reporting for specific materials, systems and methods

We require information from authors about some types of materials, experimental systems and methods used in many studies. Here, indicate whether each material, system or method listed is relevant to your study. If you are not sure if a list item applies to your research, read the appropriate section before selecting a response.

Materials & experimental systems

n/a	Involved in the study	
☒	☐	Antibodies
☒	☐	Eukaryotic cell lines
☒	☐	Palaeontology and archaeology
☒	☐	Animals and other organisms
☐	☒	Clinical data
☒	☐	Dual use research of concern
☒	☐	Plants

Methods

n/a	Involved in the study	
☒	☐	ChIP-seq
☒	☐	Flow cytometry
☒	☐	MRI-based neuroimaging

Clinical data

Policy information about clinical studies

All manuscripts should comply with the ICMJE guidelines for publication of clinical research and a completed CONSORT checklist must be included with all submissions.

Clinical trial registration	NCT05627570
Study protocol	The full study protocol has been provided with the submission of the manuscript to Nature Medicine.
Data collection	Fifty-five adults who were staff at University College London Hospital were recruited from Southeast England and London. Between April 2023 and May 2024, 135 adults underwent screening. The last participant last visit was 13 October 2024.
Outcomes	The primary outcome was the within-participant difference in percent weight change (%WC) between MPF and UPF diets at 8 weeks from baseline. This is being used clinically in weight management clinics and used for all NHS weight management programmes. Weight is an efficient and more accurate measurement to collect than other energy balance measures, such as energy intake. The hypothesised weight change in the current growing obesity pandemic would have clinically relevant impacts on minimising the rising rates of obesity and adverse impacts of adiposity-related disease.<br><br>Secondary outcomes were chosen for their relevance to changes in body composition, and wider links between ultra-processed food and poor health, and assessing mechanisms linking ultra-processed food and poor health (craving, appetite).<br><br>Primary and secondary outcome measure assessments are outlined below:<br><br>Weight was measured using an electronic scale to the nearest 0.1 kg (Tanita DC-430MAS; Tanita, Tokyo, Japan). Body composition including fat mass, body fat percentage, visceral fat rating, fat-free mass, muscle mass, bone mass, total body water mass, and total body water percentage were assessed using bioelectrical impedance analysis (BIA) (Tanita) at each visit. BIA at baseline and week 8 was conducted following an overnight fast with no alcohol intake or strenuous activity in the preceding 24 hours. Assessments at week 4 were not fasted. Participants were provided with standardised wording in the week before their baseline and week 8 visits to maintain a consistent hydration status: “Please make sure that for the visit, you eat your usual diet for the 24 hours prior to the visit day and to avoid alcohol and strenuous exercise. Please fast from 20:00pm on the night before the study visit, and drink only water.

Please do try to drink some water before the visit as this helps with the cannulation". Upon arrival, participants were asked to confirm that they had fasted and given the opportunity to drink water to thirst prior to measurements to ensure consistency. Basal metabolic rate was estimated by the Tanita BIA scanner based on fat-free mass and participant age. Height was assessed using a stadiometer to the nearest 0.5 cm. Waist circumference was measured in cm using an inelastic tape measure at the iliac crest48. BMI was derived from weight and height (in kg/m2), and waist-to-height ratio from WC and height. Estimated daily energy imbalance was assessed using the energy densities of fat mass and fat-free mass of ~9300 kcal/kg and 1100 kcal/kg, respectively49. The mean daily energy imbalance (kcal/day) for each participant for each diet was calculated as (9300*change in fat mass (kg) from baseline to week 8 + 1100*change in fat-free mass (kg) from baseline to week 8) / exact number of days from the start of the diet to the week 8 BIA assessment date. Blood pressure was recorded in triplicate, seated, alongside heart rate with an automated sphygmomanometer and oximeter. Blood pressure was recorded as the average of the second and third recordings. Venous blood samples were collected after an overnight fast and included glucose, HbA1c, liver function tests (bilirubin, alkaline phosphatase, alanine transaminase and albumin), lipids (total cholesterol, HDL-C, LDL-C, total cholesterol-to-HDL ratio, non-HDL-C and triglycerides) and CRP.

CoEQ is a 21-item validated measure of the severity and type of food cravings that an individual experiences, as well as of their inhibitory control of eating and subjective sensation of appetite and mood50. The CoEQ contains four domains: overall craving control, craving for sweet, craving for savoury and positive mood, and one question on perceived control over resisting a self-nominated craved food. PFS is a 15-item validated measure of hedonic appetite, food reward sensitivity and the psychological impact of living in food-abundant environments51. PFS assesses the appetite for and motivation to consume palatable foods at three levels: food available (but not physically present), food present (but not tasted), and food tasted (but not yet consumed)52. An overall PFS score is then computed from the mean of the three sub-scores. PFS and CoEQ were collected at baseline and at 4- and 8-week visits.

A 30-minute meal test was used to assess acute changes in subjective appetite levels in the fasted and fed state at baseline and at 8-weeks. A 5-item subjective appetite VAS was completed following an overnight fast. The questions capture aspects of hunger and the desire to eat: "How hungry do you feel right now?", "How sick do you feel right now?", "How much do you think you could eat right now?", "How full do you feel right now?" and "How pleasant would it be to eat right now?", on a 10-point 100-mm scale, with words anchored at either end marking the extremes ("Not at all", and "Extremely")53. A liquid meal (187.5ml Abbott Ensure (450kcal, 17.5g fat, 54.0g carbohydrate, 19.1g protein) was then consumed, and the subjective appetite VAS assessments were repeated at 15 and 30 minutes after starting the liquid meal.

Baseline habitual dietary intake was assessed using Intake2454, a validated, online, self-reported 24-hour recall system, based on a multiple-pass recall suitable for the general population (https://intake24.co.uk)55,56. Two non-consecutive 24-hour recalls were completed at screening, baseline, and at week 4 and week 8 on each diet. Food diaries were provided to record adherence to the diets and report any foods consumed off diet. Non-adherence was pre-specified as consuming more than one meal per week off the provided intervention diet. Participants were encouraged to report any deviations from the provided diets and to be as honest as possible, with no repercussions. All completed and returned food diaries were analysed. The research team provided multiple options and opportunities for participants to return food diaries to maximise collection, including drop-off at follow-up visits or at the research centre at participants' convenience during the trial. For any unreturned food diaries participants were followed up multiple times to drop off food diaries at the research centre at their convenience, post them at no cost, or email their food diary.

MVPA was objectively measured using wGT3X-BT (ActiGraph), an accelerometer-based activity monitor providing information on body movement using a motion sensor. The device is a reliable tool and has been widely used in clinical research given its practicality, non-invasiveness, and accuracy in measuring PA levels in free-living adults57. Participants were instructed to wear the device on their dominant hip continuously for seven days, and only to be removed for water-based activities. Average daily MVPA is a validated measure obtained from hip-worn accelerometers58. For data to be valid, participants must wear the device for at least four days with at least 10 hours of daily wear time. Wear time was validated in ActiGraph ActiLife software (v. 6.13.6), based on criteria by Troiano et al.59. Thereafter, the cut points proposed by Freedson et al.58 were applied to each participant's counts per minute data to derive the length of time spent in sedentary, light, moderate, vigorous, and very vigorous physical activity to calculate average daily MVPA.

Following completion of the RCT, participants were asked to rate both diets on a scale of 0-10, with 0 indicating a negative, poor, or bland experience, or the least intensity of the attribute being evaluated, and 10 indicating a positive, excellent, or flavourful experience, or the greatest intensity of the attribute being evaluated. Ratings were of the overall experience, of meals and snacks, of flavours and taste, of textures, of portion sizes, of delivery and required preparation, of hunger level, of happiness/contentment, and of diet sustainability. Further details on the ratings are provided in Supplementary Table 27.

Primary and secondary outcomes were assessed using mixed-effects models in an ITT analysis to assess the difference in %WC at 8-weeks and secondary outcomes, with a random effect for participants, and adjusting for randomisation arm (including interaction with diet) and night-shift status. The primary outcome effect size with 95% confidence intervals was then computed from the mixed-effects model using Cohen's d.

Plants

Seed stocks	*Report on the source of all seed stocks or other plant material used. If applicable, state the seed stock centre and catalogue number. If plant specimens were collected from the field, describe the collection location, date and sampling procedures.*
Novel plant genotypes	*Describe the methods by which all novel plant genotypes were produced. This includes those generated by transgenic approaches, gene editing, chemical/radiation-based mutagenesis and hybridization. For transgenic lines, describe the transformation method, the number of independent lines analyzed and the generation upon which experiments were performed. For gene-edited lines, describe the editor used, the endogenous sequence targeted for editing, the targeting guide RNA sequence (if applicable) and how the editor was applied.*
Authentication	*Describe any authentication procedures for each seed stock used or novel genotype generated. Describe any experiments used to assess the effect of a mutation and, where applicable, how potential secondary effects (e.g. second site T-DNA insertions, mosiacism, off-target gene editing) were examined.*
